# Adherence to the test, trace, and isolate system in the UK: results from 37 nationally representative surveys

**DOI:** 10.1136/bmj.n608

**Published:** 2021-03-31

**Authors:** Louise E Smith, Henry W W Potts, Richard Amlôt, Nicola T Fear, Susan Michie, G James Rubin

**Affiliations:** 1 Department of Psychological Medicine, Institute of Psychiatry, Psychology and Neuroscience, Weston Education Centre, King’s College London, London SE5 9RJ, UK; 2 NIHR Health Protection Research Unit in Emergency Preparedness and Response, King’s College London, London, UK; 3 Institute of Health Informatics, University College London, London, UK; 4 Public Health England, Behavioural Science Team, Emergency Response Department Science and Technology, Porton Down, Salisbury, UK; 5 King’s Centre for Military Health Research and Academic Department of Military Mental Health, King’s College London, London, UK; 6 Centre for Behaviour Change, University College London, London, UK

## Abstract

**Objective:**

To investigate rates of adherence to the UK’s test, trace, and isolate system over the initial 11 months of the covid-19 pandemic.

**Design:**

Series of cross sectional online surveys.

**Setting:**

37 nationally representative surveys in the UK, 2 March 2020 to 27 January 2021.

**Participants:**

74 699 responses from 45 957 people living in the UK, aged 16 years or older (37 survey waves, about 2000 participants in each wave).

**Main outcome measures:**

Identification of the main symptoms of covid-19 (cough, high temperature or fever, and loss of sense of smell or taste), self-reported adherence to self-isolation if symptoms were present and intention to self-isolate if symptoms were to develop, requesting a test for covid-19 if symptoms were present and intention to request a test if symptoms were to develop, and intention to share details of close contacts.

**Results:**

Only 51.5% of participants (95% confidence interval 51.0% to 51.9%, n=26 030/50  570) identified the main symptoms of covid-19; the corresponding values in the most recent wave of data collection (25-27 January 2021) were 50.8% (48.6% to 53.0%, n=1019/2007). Across all waves, duration adjusted adherence to full self-isolation was 42.5% (95% confidence interval 39.7% to 45.2%, n=515/1213); in the most recent wave of data collection (25-27 January 2021), it was 51.8% (40.8% to 62.8%, n=43/83). Across all waves, requesting a test for covid-19 was 18.0% (95% confidence interval 16.6% to 19.3%, n=552/3068), increasing to 22.2% (14.6% to 29.9%, n=26/117) from 25 to 27 January. Across all waves, intention to share details of close contacts was 79.1% (95% confidence interval 78.8% to 79.5%, n=36 145/45 680), increasing to 81.9% (80.1% to 83.6%, n=1547/1890) from 25 to 27 January. Non-adherence was associated with being male, younger age, having a dependent child in the household, lower socioeconomic status, greater financial hardship during the pandemic, and working in a key sector.

**Conclusions:**

Levels of adherence to test, trace, and isolate are low, although some improvement has occurred over time. Practical support and financial reimbursement are likely to improve adherence. Targeting messaging and policies to men, younger age groups, and key workers might also be necessary.

## Introduction

Governments around the world have relied on test, trace, and isolate strategies to separate infected people from non-infected people and prevent the spread of covid-19.^1^ Test, trace, and isolate is a less disruptive measure than alternative population-wide restrictions in activity. Within the UK, guidance for people who might have covid-19 has evolved over time but has focused on the need for people with a persistent new onset cough, fever, or loss of sense of taste or smell to remain at home for at least seven days from the onset of symptoms (self-isolate), request a test to confirm whether they have covid-19, and, if the test result is positive, provide details of close contacts to a dedicated service. These principles are the same in each of the four UK nations (England, Wales, Scotland, and Northern Ireland), although each nation has its own test, trace, and isolate system.^2^ ^3^ ^4^ ^5^

The ability of the test, trace, and isolate system to keep rates of infection under control relies on how well people adhere to guidance on testing, provide details of contacts, and self-isolate, which in turn depends on their knowledge, motivation, and opportunity to do so.^6^ ^7^ From when an infected person develops symptoms to when their contacts are allowed to come out of quarantine, adherence might break down at multiple stages.^8^ In the UK, knowledge of the symptoms of covid-19 has been shown to be poor.^9^ ^10^ Financial constraints and cramped accommodation have been identified as factors that affect whether people will remain at home during the pandemic.^10^ ^11^ ^12^ Some evidence suggests that men and younger age groups are less adherent to covid-19 restrictions,^13^ as are those who think they have been infected with SARS-CoV-2.^14^

Identifying key factors that increase or decrease adherence can be used to inform policies to improve the functioning of the test, trace, and isolate system. Since the start of the covid-19 pandemic, we have worked with England’s Department of Health and Social Care to develop and analyse a series of regular cross sectional surveys tracking relevant behaviours and their potential predictors in the UK public. We report data from 37 of these surveys that tracked adherence to the key components of the system over time and investigate personal and clinical characteristics that might be related to adherence to full self-isolation when someone has symptoms, requesting a test if symptoms are present, and intending to share details of close contacts if symptomatic. We also investigated variables associated with correctly identifying the main symptoms of covid-19.

## Methods

### Design

BMG Research, a Market Research Society company partner, conducted a series of cross sectional online surveys on behalf of the Department of Health and Social Care starting on 28 January 2020, which we analysed as part of the CORSAIR (the COVID-19 Rapid Survey of Adherence to Interventions and Responses) study. Surveys were conducted weekly until 1 July (wave 23), after which survey waves were fortnightly; the weekly survey was resumed between 9 November 2020 and 13 January 2021. No data were collected in mid-August 2020. We used data from surveys conducted between 2 March 2020 (wave 6) and 27 January 2021 (wave 42). Data were collected over a three day period (Monday to Wednesday) for each survey wave, except for wave 6 (collected Monday to Thursday) and waves 12, 18, and 27 (collected Tuesday to Wednesday). As prompt turnaround of data collection is essential during a rapidly evolving crisis,^15^ the surveys used standard opinion polling methods using non-probability sampling, an approach common within market research, political polling, and social science.^16^ Quota samples aim to minimise response bias by filling predetermined targets so that the social and personal characteristics of the participants match those of the national population. As such, participants who belong to a quota that has already been met are prevented from completing the survey. Therefore, response rates are not useful indicators of response bias in quota samples and are not usually reported.

### Participants

This study reports on 74 699 responses from 45 957 participants across the four UK nations. Participants (about 2000 in each wave) were recruited from two specialist research panel providers, Respondi (n=50 000) and Savanta (n=31 500).^17^ ^18^ Participants in the first seven waves were recruited from Respondi only; subsequent waves included roughly equal numbers from each panel. Participants were eligible for the study if they were aged 16 years or older and lived in the UK. Respondents who completed the survey were unable to participate in the following three waves. Owing to an error, a few people completed waves more often than others; 28 people (0.1% of our sample) completed 10 waves or more. Quotas were applied based on age and sex (combined) and government office region and reflected targets based on data from the Office for National Statistics.^19^ Therefore, the sociodemographic characteristics of participants in each survey wave were broadly similar to those in the UK general population. Participants were reimbursed in points, which could be redeemed in cash, gift vouchers, or charitable donations (up to £0.70 ($0.98; €0.81) for each survey).

### Outcome measures

*Identification of covid-19 symptoms*—One question asked participants to identify the most common symptoms of covid-19, with multiple response options allowed (up to four initially, up to five from 25 May 2020, wave 18). We coded participants as having identified symptoms of covid-19 if they selected cough, high temperature or fever, and, from 18 May 2020 (wave 17), either loss of sense of smell or loss of sense of taste. In government guidance these symptoms are actively promoted to members of the UK public as the “main” symptoms of covid-19.^20^

*Fully self-isolating*—We measured self-reported self-isolation in participants who indicated that they had experienced symptoms of covid-19 (high temperature or fever, cough, or loss of sense of smell or taste) in the past seven days. Participants were asked for what reason, if any, they had left home since the development of symptoms. We categorised people as non-adherent if they reported leaving home for any reason since symptoms developed. From 26 October 2020 (wave 31) we also asked participants how soon (in days) they had first left home after symptoms developed. We used this to create a second outcome variable (duration adjusted adherence) and categorised people as non-adherent if they reported leaving home for any reason in the first 10 days after symptoms developed. This adjustment allowed for the fact that, during that period, self-isolation was only required for 10 days after symptom onset. We measured intended full self-isolation in participants who had not experienced covid-19 symptoms in the past week. Participants were asked to imagine they developed symptoms of covid-19 (high temperature or fever, new continuous cough, or loss of sense of taste or smell) the next morning and what would cause them to leave home, if anything.

*Requesting a test*—Participants who reported covid-19 symptoms were asked what actions they had taken when symptoms developed. Response options included “I requested a test to confirm whether I have coronavirus.” In data collected between 1 June and 5 August 2020 (waves 19 to 26), participants who reported requesting a test after symptoms had developed were asked whether the test indicated they had or did not have covid-19 or if they were still waiting for the test results. Participants who reported no covid-19 symptoms were asked what actions they would take if they were to develop symptoms.

*Sharing details of close contacts*—Participants who had not experienced covid-19 symptoms in the past seven days were asked to imagine they had tested positive for covid-19 and had been prompted by the National Health Service contact tracing service. We asked participants how likely they would then be to share details of people they had been in close contact with on a 5 point scale from “definitely would” to “definitely would not.” We recoded intention to share details of close contacts into a binary variable (probably or definitely would share details versus not sure, probably, or definitely would not). Too few participants indicated that they had tested positive to analyse separately.

### Personal and clinical characteristics

We asked participants to report their age, sex, employment status, highest educational or professional qualification, ethnicity, and marital status, and the number of people living in their household. Participants also reported the occupation of the highest earner in the household, whether a dependent child lived in the household, they or a household member had a chronic illness, they worked in a key sector, or they were self-employed. Participants were asked for their full postcode, from which we determined region and indices of multiple deprivation.^21^

We coded participants as having a chronic illness that made them clinically vulnerable to covid-19 using guidance from the NHS website.^22^ Participants were categorised as working in a key sector if they worked in one of several sectors specified in government guidance.^23^

Participants were asked if they thought they “had, or currently have, coronavirus.” Those who reported having experienced symptoms of covid-19 in the past week were asked what they thought had caused their symptoms (symptom attribution). We measured financial hardship by asking participants to what extent in the past seven days they had been struggling to make ends meet, skipping meals, and finding their current living situation difficult (Cronbach’s α=0.75).

### Power calculation

We determined that a sample size of 2000 in each survey would allow a 95% confidence interval of plus or minus 2% for the prevalence estimate for a survey item with a prevalence of around 50%. In practice, power was considerably better as we pooled data from survey waves.

### Statistical analysis

Owing to an error in collecting data about chronic illness on 26 to 28 October 2020 (wave 31), these data were excluded from analyses investigating factors associated with outcome variables. Responder IDs were not assigned for 4.3% of participants (n=6381/149 640). These responses were also excluded from analyses. We used generalised estimating equations (with an exchangeable correlation structure) to correct for some participants being in more than one wave. Generalised estimating equations were used to investigate factors associated with identifying cough, high temperature or fever, and loss of sense of smell or taste (25 May 2020 to 27 January 2021, excluding data collected 26-38 October 2020; wave 31), full self-isolation (14 April 2020 to 27 January 2021, excluding data collected 26-38 October; wave 31), duration adjusted adherence to full self-isolation (9 November 2020 to 27 January 2021), requesting a test (25 May 2020 to 27 January 2021, excluding data collected 26-38 October; wave 31), and intention to share details of close contacts if a covid-19 test result was positive (1 June 2020 to 27 January 2021, excluding data collected 26-38 October 2020; wave 31). 

Between 14 April 2020 and 27 January 2021, excluding data collected from 26 to 28 October 2020 (wave 31) and those for whom a unique response ID was not assigned (ie, data points included in generalised estimating equations analyses), there were 59 237 responses from 40 112 participants. Overall, 30 257 participants (75.4%) answered one survey and 9855 participants (24.6%) answered more than one survey (see supplementary file for numbers of responses and participants included in each analysis). 

For each set of analyses, we ran univariable and multivariable analyses. Multivariable regressions adjusted for survey wave, region (with East Midlands arbitrarily allocated as reference category), sex, age (raw and quadratic term), a dependent child in the household, being clinically vulnerable to covid-19, having a household member with a chronic illness, employment status (working *v* not working), highest earner works in a manual occupation (no *v* yes),^24^ index of multiple deprivation (fourths), highest educational or professional qualification (degree or higher *v* less than degree), ethnicity (white British (reference category), white other, mixed, Asian or Asian British, black or black British, Arab or other, don’t know or prefer not to say), and living alone. Loess plots of age effects suggested quadratic relations would be appropriate.

Only participants who reported covid-19 symptoms in the past week were included in analyses of full self-isolation (n=3397 responses; see supplementary file); duration adjusted self-isolation (n=1102 responses) and requesting a test (n=2920 responses).

It was permissible to leave home during the self-isolation period to get tested or if a covid-19 test result was negative.^25^ For self-isolation analyses, we excluded those who reported a negative test result in one of several closed questions or in free text since their symptoms developed or in the past week (see supplementary file).

In analyses of factors associated with self-reported self-isolation and requesting a test, we recoded ethnicity into three categories owing to small numbers of cases: white British (reference category); white other; and black, Asian, mixed, or other (people who preferred not to say were excluded). For analyses of factors associated with self-reported self-isolation accounting for duration of isolation, we also merged participants in the north east and north west and participants in Scotland, Wales, and Northern Ireland into single groups.

Many analyses were conducted on each outcome variable (about 47). Uncorrected P values are given shown in the table; we only report narratively on results that remained statistically significant after applying a conservative Bonferroni correction (P<0.001).

Before the analyses reported here, we analysed survey results at multiple time points. Results were reported regularly to the Department of Health and Social Care and the UK Scientific Advisory Group for Emergencies (SAGE).

### Sensitivity analysis

Socioeconomic grade can be derived from the question asking the occupation of the highest earner in the household (see supplementary file). We conducted a sensitivity analysis for adjusted generalised estimating equations, including socioeconomic grade as an explanatory variable and removing highest earner working in a manual occupation. 

### Patient and public involvement

Lay members served on the advisory group for the project that developed our prototype survey material; this included three rounds of qualitative testing.^26^ Owing to the rapid nature of this research during the covid-19 pandemic, the public was not involved in further developments of the materials.

## Results

### Identification of covid-19 symptoms

**Fig 1:**
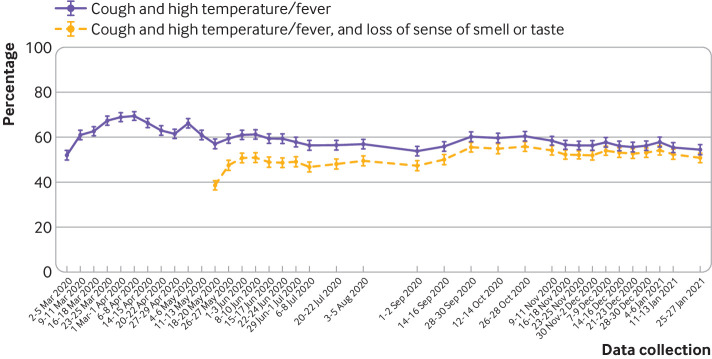
Percentage of people who correctly identified the most common symptoms of covid-19. Error bars are 95% confidence intervals

When data from 26 May 2020 to 27 January 2021 (waves 18-42) were combined, 51.5% of participants (95% confidence interval 51.0% to 51.9%, n= 26 030/50 570) identified cough, high temperature or fever, and loss of sense of smell or taste as symptoms of covid-19. Recognition initially increased at the start of data collection and when loss of sense of smell or taste was introduced into government guidance,^20^ after which it remained relatively stable (fig 1). In the latest available wave of data collection (wave 42, 25-27 January 2021), 50.8% (48.6% to 53.0%, n=1019/2007) of participants identified the symptoms of cough, high temperature or fever, and loss of sense of smell or taste. When analysis was restricted to recognition of cough and high temperature or fever alone, the results were similar. The supplementary file presents rates of recognition for individual symptoms.

Correct identification of covid-19 symptoms was associated with being female, older (see supplementary file), identifying as white British, a belief of not having had covid-19, lesser financial hardship, highest earner not working in a manual occupation, living in less deprived areas, no dependent child in the household, not living alone, and not working in key sectors (table 1). Those who lived in London were less likely to identify symptoms of covid-19 (adjusted odds ratio 0.76, 95% confidence interval 0.69 to 0.84, compared with the baseline region, East Midlands; see supplementary file). Variation by survey wave was significant, although no individual wave reached our significance level.

**Table 1:** Associations between personal and clinical characteristics and correctly identifying high temperature or fever, cough, and loss of sense of smell or taste as main symptoms of covid-19

Characteristics	Identification of covid-19 symptoms		Odds ratio (95% CI) for correct identification	P value	Adjusted odds ratio (95% CI) for correct identification*	P value	Adjusted odds ratio (95% CI) for correct identification†	P value
	Not correctly identified (n=23 440)	Correctly identified (n=24 728)						
Survey wave overall	-	-	χ^2^(23)=104.0	<0.001	χ^2^(23)=79.6	<0.001	χ^2^(23)=80.5	<.001
Region overall	-	-	χ^2^(11)=261.8	<0.001	χ^2^(11)=58.1	<0.001	χ^2^(11)=61.8	<.001
Male	12 099 (55.0)	9897 (45.0)	Reference	-	Reference	-	Reference	-
Female	11 267 (43.3)	14 766 (56.7)	1.62 (1.56 to 1.69)	<0.001	1.76 (1.69 to 1.84)	<0.001	1.77 (1.69 to 1.84)	<0.001
Raw age (per decade) (years)	Mean 45.4 (SD 17.7)	Mean 50.9 (SD 17.0)	1.20 (1.18 to 1.21)	<0.001	1.17 (1.15 to 1.19)	<0.001	1.17 (1.15 to 1.18)	<0.001
Age: quadratic (age−mean)^2^	-	-	-	-	0.9997 (0.9996 to 0.9998)	<0.001	0.99965 (0.99958 to 0.99973)	<0.001
Dependent child in household:								
No	15 299 (46.3)	17 732 (53.7)	Reference	-	Reference	-	Reference	-
Yes	8141 (53.8)	6996 (46.2)	0.76 (0.72 to 0.79)	<0.001	0.90 (0.86 to 0.95)	<0.001	0.90 (0.86 to 0.95)	<0.001
Clinically vulnerable to covid-19:								
No	17 941 (48.6)	18 983 (51.4)	Reference	-	Reference	-	Reference	-
Yes	4325 (46.2)	5043 (53.8)	1.08 (1.03 to 1.14)	0.001	0.96 (0.92 to 1.01)	0.15	0.98 (0.93 to 1.03)	0.42
Household member has chronic illness:								
No	19 024 (48.6)	20 148 (51.4)	Reference	-	Reference	-	Reference	-
Yes	3767 (47.7)	4123 (52.3)	1.03 (0.98 to 1.08)	0.26	0.99 (0.93 to 1.04)	0.60	1.00 (0.95 to 1.06)	1.00
Employed:								
No	9988 (46.3)	11 600 (53.7)	Reference	-	Reference	-	Reference	-
Yes	13 048 (50.3)	12 890 (49.7)	0.88 (0.85 to 0.92)	<0.001	1.07 (1.02 to 1.13)	0.003	1.00 (0.96 to 1.05)	0.91
Highest earner works in a manual occupation‡:								
No	15 541 (46.4)	17 967 (53.6)	Reference	-	Reference	-	-	-
Yes	7389 (54.1)	6273 (45.9)	0.73 (0.70 to 0.76)	<0.001	0.82 (0.78 to 0.86)	<0.001	-	-
Socioeconomic grade:								
ABC1 (high)	11 995 (45.4)	14 400 (54.6)	Reference	-	-	-	Reference	-
C2DE	10 935 (52.6)	9840 (47.4)	0.74 (0.72 to 0.77)	<0.001	-	-	0.76 (0.73 to 0.80)	<0.001
Index of multiple deprivation fourth:								
1st (least deprived)	4518 (43.4)	5883 (56.6)	1.57 (1.49 to 1.66)	<0.001	1.26 (1.18 to 1.34)	<0.001	1.22 (1.14 to 1.29)	<0.001
2nd	5256 (45.4)	6313 (54.6)	1.44 (1.36 to 1.52)	<0.001	1.19 (1.12 to 1.27)	<0.001	1.16 (1.10 to 1.24)	<0.001
3rd	6373 (49.7)	6445 (50.3)	1.21 (1.15 to 1.28)	<0.001	1.10 (1.04 to 1.16)	0.001	1.09 (1.03 to 1.15)	0.005
4th (most deprived)	7293 (54.5)	6087 (45.5)	Reference	-	Reference	-	Reference	-
Overall	-	-	χ^2^(3)=302.5	<0.001	χ^2^(3)=59.0	<0.001	χ^2^(3)=43.0	<.001
Highest educational or professional qualification:								
GCSE, vocational, A level, or no formal qualifications	15 395 (48.5)	16 337 (51.5)	Reference	-	Reference	-	Reference	-
Degree or higher (bachelors, masters, or PhD)	8045 (48.9)	8391 (51.1)	0.98 (0.95 to 1.03)	0.45	1.06 (1.02 to 1.11)	0.009	1.03 (0.99 to 1.08)	0.19
Ethnicity:								
White British	18 616 (46.2)	21 722 (53.8)	Reference	-	Reference	-	Reference	-
White other	1949 (59.8)	1310 (40.2)	0.56 (0.52 to 0.61)	<0.001	0.69 (0.63 to 0.75)	<0.001	0.69 (0.63 to 0.75)	<0.001
Mixed	656 (63.1)	384 (36.9)	0.51 (0.45 to 0.58)	<0.001	0.63 (0.54 to 0.72)	<0.001	0.63 (0.55 to 0.73)	<0.001
Asian or Asian British	1327 (61.4)	836 (38.6)	0.55 (0.50 to 0.61)	<0.001	0.74 (0.66 to 0.82)	<0.001	0.73 (0.66 to 0.81)	<0.001
Black or black British	618 (68.3)	287 (31.7)	0.40 (0.34 to 0.47)	<0.001	0.54 (0.46 to 0.64)	<0.001	0.54 (0.45 to 0.64)	<0.001
Arab or other	104 (55.9)	82 (44.1)	0.67 (0.50 to 0.91)	0.01	0.78 (0.57 to 1.09)	0.15	0.77 (0.56 to 1.07)	0.12
Don’t know or prefer not to say	170 (61.4)	107 (38.6)	0.57 (0.44 to 0.74)	<0.001	0.85 (0.60 to 1.20)	0.36	0.86 (0.61 to 1.20)	0.37
Overall	-	-	χ^2^(6)=534.0	<0.001	χ^2^(6)=161.0	<0.001	χ^2^(6)=165.6	<.001
Live alone:								
No	18 830 (48.8)	19 740 (51.2)	Reference	-	Reference	-	Reference	-
Yes	4610 (48.0)	4988 (52.0)	1.01 (0.96 to 1.06)	0.69	0.87 (0.82 to 0.92)	<0.001	0.89 (0.85 to 0.95)	<0.001
Work in key sector:								
No	6224 (49.7)	6312 (50.3)	Reference	-	Reference	-	Reference	-
Yes	8424 (52.4)	7657 (47.6)	0.89 (0.85 to 0.93)	<0.001	0.91 (0.86 to 0.96)	<0.001	0.91 (0.86 to 0.96)	<0.001
Self-employed§:								
No	12 147 (50.3)	11 981 (49.7)	Reference	-	Reference	-	Reference	-
Yes	901 (49.8)	909 (50.2)	0.99 (0.90 to 1.10)	0.85	0.92 (0.83 to 1.03)	0.15	0.93 (0.83 to 1.04)	0.20
Marital status:								
Single, separated, divorced, or widowed	9757 (51.3)	9271 (48.7)	Reference	-	Reference	-	Reference	-
Married or partnered	13 286 (46.4)	15 318 (53.6)	1.23 (1.18 to 1.28)	<0.001	1.07 (1.02 to 1.13)	0.01	1.06 (1.01 to 1.12)	0.03
Ever had covid-19:								
Think not	19 242 (46.6)	22 019 (53.4)	Reference	-	Reference	-	Reference	-
Think so, or confirmed	4198 (60.8)	2709 (39.2)	0.60 (0.57 to 0.63)	<0.001	0.71 (0.67 to 0.75)	<0.001	0.71 (0.67 to 0.75)	<0.001
Hardship¶	n=22 323; mean 8.4 (SD 3.0)	n=23 932; mean 7.4 (SD 2.8)	0.896 (0.890 to 0.902)	<0.001	0.927 (0.920 to 0.934)	<0.001	0.93 (0.92 to 0.94)	<0.001

SD=standard deviation; CI=confidence interval. For continuous variables, odds ratios represent a one unit increase in the explanatory variable, apart from age, when odds ratios represent a 10 year increase in age.

* Adjusted for survey wave, region, sex, age (raw and quadratic term), dependent child in household, clinically vulnerable to covid-19, household member has chronic illness, employment status, highest earner works in a manual occupation, index of multiple deprivation, highest educational or professional qualification, ethnicity, and living alone.

† Adjusting for survey wave, region, sex, age (raw and quadratic term), dependent child in the household, clinically vulnerable to covid-19, household member has chronic illness, employment status, socioeconomic grade, index of multiple deprivation, highest educational or professional qualification, ethnicity, and living alone.

‡ For most analyses, an exchangeable correlation structure was used—this failed to converge for the univariable analysis for region, so an unstructured correlation structure was used.

§ Not adjusted for employment status as by definition all people who were asked whether they were self-employed were working.

¶ From 3 (least hardship) to 15 (most hardship).

Results did not differ in a sensitivity analysis adjusting for socioeconomic grade rather than the highest earner being a manual worker (table 1 and supplementary file).

### Fully self-isolating when symptomatic

**Fig 2:**
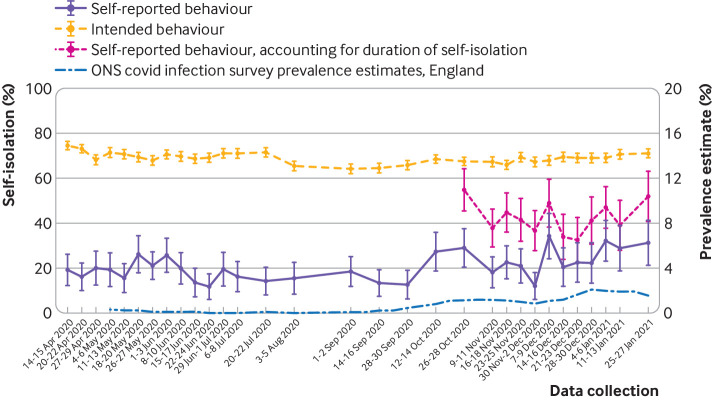
Percentage of people who reported not leaving home since developing symptoms of covid-19 (in those who had experienced covid-19 symptoms in the past seven days, excluding those who had received a negative covid-19 test result since developing symptoms) and who reported no intention to leave home if they were to develop covid-19 symptoms (in people who had not had covid-19 symptoms in the past seven days). Prevalence estimates, using the Office for National Statistics (ONS) covid-19 survey are also included. Error bars are 95% confidence intervals

Combining data from 14 April 2020 to 27 January 2021 (waves 12 to 42), of those who reported having experienced symptoms of covid-19 in the past seven days (excluding those who reported receiving a negative covid-19 test result since having developed symptoms), only 20.2% (95% confidence interval 18.8% to 21.5%, n=720/3567) said they had not left home since developing symptoms. The percentage of people who reported full self-isolation was largely stable until October 2020 and then increased (fig 2). In the latest wave of data collection (wave 42, 25-27 January 2021), the percentage of people who reported not leaving home after symptoms developed was 31.3% (21.1% to 41.5%, n=26/83). From 26 October 2020 to 27 January 2021 (waves 31 to 42), duration adjusted adherence to full self-isolation was 42.5% (39.7% to 45.2%, n=515/1213). In the latest wave of data collection (wave 42, 25-27 January 2021), duration adjusted adherence was 51.8% (40.8% to 62.8%, n=43/83). Intention to fully self-isolate if symptoms of covid-19 were to develop was much higher, at around 70%, and was 71.0% (68.9% to 73.0%, n=1341/1890) in the latest wave of data collection (wave 42).

No associations between duration adjusted self-isolation and any personal or clinical characteristic were significant after applying a conservative Bonferroni correction (table 2).

**Table 2:** Associations between personal and clinical characteristics and full self-isolation adjusted for duration of isolation after developing symptoms of covid-19

Characteristics	Self-isolating status		Odds ratio (95% CI) for fully self-isolating	P value	Adjusted odds ratio (95% CI) for fully self-isolating*	P value	Adjusted odds ratio (95% CI) for fully self-isolating†	P value
	Not fully self-isolating (n=648)	Fully self-isolating (n=454)						
Survey wave overall	-	-	χ^2^(10)=14.0	0.17	χ^2^(10)=16.8	0.08	χ^2^(10)=16.8	0.08
Region overall	-	-	χ^2^(8)=2.5	0.96	χ^2^(8)=2.9	0.94	χ^2^(8)=2.9	0.94
Male	376 (62.9)	222 (37.1)	Reference	-	Reference	-	Reference	-
Female	269 (54.3)	226 (45.7)	1.41 (1.10 to 1.80)	0.006	1.50 (1.14 to 1.97)	0.004	1.49 (1.13 to 1.97)	0.004
Raw age (per decade) (years)	Mean 36.6 (SD 14.4)	Mean 39.7 (SD 16.1)	1.14 (1.05 to 1.24)	0.002	1.16 (1.04 to 1.30)	0.007	1.16 (1.04 to 1.30)	0.007
Age: quadratic (age−mean)^2^	-	-	-	-	0.9999 (0.9994 to 1.0004)	0.67	0.9999 (0.9994 to 1.0004)	0.66
Dependent child in household:								
No	281 (54.4)	236 (45.6)	Reference	-	Reference	-	Reference	-
Yes	367 (62.7)	218 (37.3)	0.71 (0.56 to 0.91)	0.007	0.88 (0.64 to 1.21)	0.43	0.88 (0.64 to 1.21)	0.43
Clinically vulnerable to covid-19:								
No	428 (59.0)	297 (41.0)	Reference	-	Reference	-	Reference	-
Yes	167 (58.2)	120 (41.8)	1.03 (0.78 to 1.37)	0.81	0.92 (0.68 to 1.25)	0.59	0.92 (0.67 to 1.25)	0.58
Household member has chronic illness:								
No	513 (58.3)	367 (41.7)	Reference	-	Reference	-	Reference	-
Yes	118 (60.8)	76 (39.2)	0.88 (0.64 to 1.21)	0.45	0.79 (0.54 to 1.15)	0.21	0.79 (0.54 to 1.14)	0.21
Employed:								
No	196 (54.9)	161 (45.1)	Reference	-	Reference	-	Reference	-
Yes	446 (61.3)	281 (38.7)	0.78 (0.60 to 1.00)	0.05	0.85 (0.61 to 1.17)	0.31	0.84 (0.61 to 1.17)	0.31
Highest earner works in a manual occupation:								
No	343 (57.4)	255 (42.6)	Reference	-	Reference	-	-	-
Yes	295 (61.0)	189 (39.0)	0.86 (0.68 to 1.10)	0.22	0.97 (0.73 to 1.29)	0.85	-	-
Socioeconomic grade:								
ABC1 (high)	286 (59.8)	192 (40.2)	Reference	-	-	-	Reference	-
C2DE	352 (58.3)	252 (41.7)	1.06 (0.83 to 1.35)	0.65	-	-	1.00 (0.76 to 1.33)	0.99
Index of multiple deprivation fourth:								
1st (least deprived)	89 (59.3)	61 (40.7)	1.07 (0.73 to 1.56)	0.75	0.89 (0.56 to 1.41)	0.61	0.89 (0.56 to 1.41)	0.61
2nd	111 (52.1)	102 (47.9)	1.43 (1.02 to 2.01)	0.04	1.39 (0.93 to 2.06)	0.11	1.39 (0.93 to 2.06)	0.11
3rd	189 (60.4)	124 (39.6)	1.02 (0.76 to 1.38)	0.88	0.89 (0.63 to 1.27)	0.53	0.89 (0.63 to 1.27)	0.53
4th (most deprived)	259 (60.8)	167 (39.2)	Reference	-	Reference	-	Reference	-
Overall	-	-	χ^2^(3)=4.9	0.18	χ^2^(3)=5.7	0.13	χ^2^(3)=5.6	0.13
Highest educational or professional qualification:								
GCSE, vocational, A level, or no formal qualifications	357 (57.4)	265 (42.6)	Reference	-	Reference	-	Reference	-
Degree or higher (bachelors, masters, or PhD)	291 (60.6)	189 (39.4)	0.87 (0.69 to 1.12)	0.28	0.97 (0.73 to 1.29)	0.84	0.97 (0.73 to 1.29)	0.85
Ethnicity:								
White British	455 (59.2)	314 (40.8)	Reference	-	Reference	-	Reference	-
White other	90 (65.2)	48 (34.8)	0.78 (0.54 to 1.14)	0.20	0.85 (0.55 to 1.31)	0.45	0.84 (0.55 to 1.30)	0.44
Black and minority ethnicity	101 (53.7)	87 (46.3)	1.26 (0.91 to 1.74)	0.16	1.66 (1.14 to 2.41)	0.008	1.66 (1.14 to 2.41)	0.008
Overall	-	-	χ^2^(2)=4.3	0.12	χ^2^(2)=8.8	0.01	χ^2^(2)=8.8	0.01
Live alone:								
No	549 (59.3)	377 (40.7)	Reference	-	Reference	-	Reference	-
Yes	99 (56.3)	77 (43.8)	1.12 (0.81 to 1.56)	0.48	0.95 (0.63 to 1.44)	0.82	0.95 (0.63 to 1.45)	0.82
Work in key sector:								
No	121 (54.5)	101 (45.5)	Reference	-	Reference	-	Reference	-
Yes	382 (63.2)	222 (36.8)	0.70 (0.51 to 0.95)	0.02	0.83 (0.58 to 1.20)	0.33	0.83 (0.58 to 1.20)	0.32
Self-employed‡:								
No	410 (61.5)	257 (38.5)	Reference	-	Reference	-	Reference	-
Yes	36 (60.0)	24 (40.0)	1.08 (0.63 to 1.85)	0.78	1.31 (0.66 to 2.61)	0.44	1.32 (0.66 to 2.62)	0.43
Marital status:								
Single, separated, divorced, or widowed	261 (56.6)	200 (43.4)	Reference	-	Reference	-	Reference	-
Married or partnered	365 (60.1)	242 (39.9)	0.86 (0.68 to 1.11)	0.25	0.87 (0.64 to 1.19)	0.38	0.87 (0.64 to 1.19)	0.39
Ever had covid-19:								
Think not	371 (57.8)	271 (42.2)	Reference	-	Reference	-	Reference	-
Think so, or confirmed	277 (60.2)	183 (39.8)	0.91 (0.71 to 1.16)	0.44	1.02 (0.77 to 1.34)	0.90	1.02 (0.77 to 1.35)	0.90
Attribute current symptoms to covid-19:								
No	494 (60.7)	320 (39.3)	Reference	-	Reference	-	Reference	-
Yes	154 (53.5)	134 (46.5)	1.33 (1.02 to 1.74)	0.04	1.49 (1.10 to 2.02)	0.01	1.49 (1.10 to 2.02)	0.01
Hardship§	n=622; mean 10.2 (SD 2.6)	n=427; mean 10.0 (SD 2.9)	0.97 (0.93 to 1.01)	0.17	0.99 (0.93 to 1.04)	0.60	0.99 (0.93 to 1.04)	0.60

SD=standard deviation; CI=confidence interval. For continuous variables, odds ratios represent a one unit increase in the explanatory variable, apart from age, when odds ratios represent a 10 year increase in age.

* Adjusted for survey wave, region, sex, age (raw and quadratic term), dependent child in household, clinically vulnerable to covid-19, household member has chronic illness, employment status, highest earner works in a manual occupation, index of multiple deprivation, highest educational or professional qualification, ethnicity, and living alone.

† Adjusting for survey wave, region, sex, age (raw and quadratic term), dependent child in the household, clinically vulnerable to covid-19, household member has chronic illness, employment status, socioeconomic grade, index of multiple deprivation, highest educational or professional qualification, ethnicity, and living alone.

‡ Not adjusted for employment status as by definition all people who were asked whether they were self-employed were working.

§ From 3 (least hardship) to 15 (most hardship).

Results did not differ in a sensitivity analysis adjusting for socioeconomic grade rather than the highest earner being a manual worker (table 2 and supplementary file).

Adherence to full self-isolation was associated with not working in a key sector (working in a key sector: adjusted odds ratio 0.51, 95% confidence interval 0.39 to 0.68), thinking you had not experienced covid-19 (thinking you had experienced covid-19 or covid-19 had been confirmed: 0.59, 0.48 to 0.73), female sex (1.87, 1.53 to 2.29), older age (1.28, 1.19 to 1.38), lower education (degree or higher: 0.61, 0.50 to 0.74), highest earner not working in a manual occupation (highest earner works in a manual occupation: 0.69, 0.56 to 0.84), and lesser financial hardship (0.91, 0.87 to 0.95; see supplementary file).

When including socioeconomic grade as an explanatory variable, rather than highest earner working in a manual occupation, the association between full self-isolation and lower socioeconomic grade (C2DE) did not reach the threshold for significance (supplementary file). However, adjusted odds ratios were similar for highest earner working in a manual occupation and lower socioeconomic grade.

When data from 26 October 2020 to 27 January 2021 (waves 31 to 42) were combined, the most frequently reported reasons for not fully self-isolating were to go to the shops for groceries or to a pharmacy (21.5%), to go to work (15.8%), to go to the shops for things other than groceries or pharmacy goods (15.6%), because symptoms did not persist or were temporary (15.2%), to go out for a medical need other than covid-19 (15.0%), to go for a walk or for some other exercise (14.8%), believing symptoms were only mild (14.5%), because symptoms got better (13.9%), thinking it was not necessary to stay at home (13.2%), being too bored (12.2%), to help or provide care for a vulnerable person (11.9%), to meet up with friends or family, or both (11.3%), and being too depressed or anxious (11.2%; see supplementary file).

### Requesting a test when symptomatic

**Fig 3:**
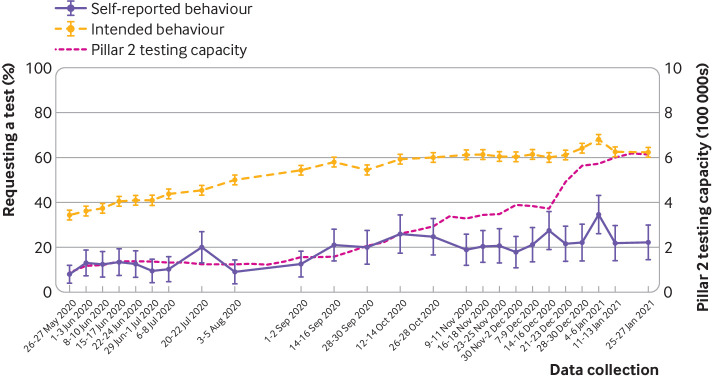
Percentage of people who reported requesting a test after developing covid-19 symptoms (in those who had experienced covid-19 symptoms in the past seven days), and who reported intending to request a test if they were to develop covid-19 symptoms (in people who had not had covid-19 symptoms in the past seven days). Pillar 2 testing capacity is also included.^27^ Error bars are 95% confidence intervals

When data from 26 May 2020 to 27 January 2021 (waves 18 to 42) were combined, of those who reported experiencing covid-19 symptoms in the past seven days, only 18.0% (95% confidence interval 16.6% to 19.3%, n=552/3068) reported requesting a test. In the latest wave of data collection (wave 42, 25-27 January 2021), the percentage of people requesting a test after symptoms developed was 22.2% (14.6% to 29.9%, n=26/117). Self-reported behaviour and intention to request a test when symptomatic increased over time. In the latest wave of data collection (wave 42, 25-27 January 2021), intention to request a test when symptomatic was 62.3% (60.1% to 64.5%, n=1178/1890) (fig 3).

Survey waves varied considerably. Participants in later waves were more likely to report requesting a test when symptomatic compared with those in wave 18 (see supplementary file). Requesting a test for covid-19 was associated with people thinking that their current symptoms could be due to covid-19 (adjusted odds ratio 1.81, 95% confidence interval 1.42 to 2.31) and lesser financial hardship (0.93, 0.89 to 0.97; see supplementary file).

Results did not differ in sensitivity analyses adjusting for socioeconomic grade (see supplementary file).

Self-reported reasons for not requesting a test were included from 8 June 2020 (wave 20). When data from 8 June 2020 to 27 January 2021 (wave 42) were combined, the most common reasons for not requesting a test were thinking the symptoms were not due to covid-19 (20.9%), symptoms had improved (16.9%), symptoms were only mild (16.3%), having no contact with anyone who had covid-19 recently (13.0%), thinking that only self-isolation was needed (11.5%), not wanting to use a test that someone needed more (11.1%), not thinking you were eligible to get a test (11.0%), and being worried about how colleagues or employers would react if a test result was positive (10.0%; see supplementary file).

### Sharing details of close contacts

**Fig 4:**
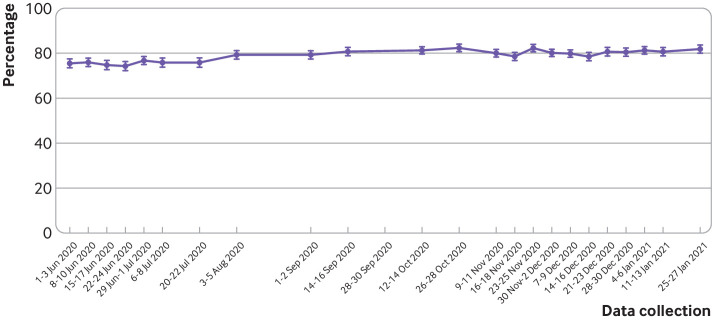
Percentage of people who reported that they probably or definitely would share details of close contacts if contacted by the NHS contact tracing service (in people who had not had covid-19 symptoms in the past seven days). Error bars are 95% confidence intervals

When data from 1 June 2020 to 27 January 2021 (wave 19 to 42) were combined, of those who had not experienced covid-19 symptoms in the past seven days, 79.1% (78.8% to 79.5%, n=36 145/45 680) reported that they probably or definitely would share details of close contacts with the NHS contact tracing service if they tested positive for covid-19 and were prompted by the NHS contact tracing service (fig 4). Intention to share details of close contacts increased slightly over time. In the latest wave of data collection (wave 42, 25-27 January 2021), 81.9% (80.1% to 83.6%, n=1547/1890) intended to share details of close contacts.

Intending to share details of close contacts was associated with being female, older, living in less deprived areas, higher education, highest earner not working in a manual occupation, being clinically vulnerable to covid-19, being married or partnered, working, not living alone, and lesser financial hardship (table 3). Not intending to share details of close contacts was associated with preferring not to disclose ethnicity. Survey waves varied considerably, with participants showing greater intention to share details of close contacts in later waves (see supplementary file).

**Table 3:** Associations between personal and clinical characteristics and intending to share details of close contacts with the NHS contact tracing service

Characteristics	Intention to share details of close contacts		Odds ratio (95% CI) for sharing details	P value	Adjusted odds ratio (95% CI) for sharing details*	P value	Adjusted odds ratio (95% CI) for sharing details†	P value
	Probably or definitely would not or not sure (n=9138)	Probably or definitely would (n=34 299)						
Survey wave overall	-	-	χ^2^(22)=132.7	<0.001	χ^2^(22)=88.4	<0.001	χ^2^(22)=89.4	<0.001
Region overall	-	-	χ^2^(11)=85.9	<0.001	χ^2^(11)=23.5	0.02	χ^2^(11)=24.2	.01
Male	4603 (23.5)	15 013 (76.5)	Reference	-	Reference	-	Reference	-
Female	4491 (19.0)	19 207 (81.0)	1.28 (1.22 to 1.35)	<0.001	1.39 (1.32 to 1.47)	<0.001	1.40 (1.32 to 1.48)	<0.001
Raw age (per decade) (years)	Mean 44.5 (SD 16.2)	Mean 50.3 (SD 17.5)	1.21 (1.19 to 1.23)	<0.001	1.23 (1.21 to 1.26)	<0.001	1.23 (1.20 to 1.25)	<0.001
Age: quadratic (age−mean)^2^	-	-	-	-	1.0006 (1.0005 to 1.0007)	<0.001	1.0006 (1.0005 to 1.0007)	<0.001
Dependent child in household:								
No	6084 (20.0)	24 410 (80.0)	Reference	-	Reference	-	Reference	-
Yes	3054 (23.6)	9889 (76.4)	0.80 (0.76 to 0.84)	<0.001	1.00 (0.94 to 1.07)	1.00	1.00 (0.94 to 1.07)	0.97
Clinically vulnerable to covid-19:								
No	7238 (21.5)	26 363 (78.5)	Reference	-	Reference	-	Reference	-
Yes	1354 (16.4)	6884 (83.6)	1.39 (1.30 to 1.49)	<0.001	1.24 (1.16 to 1.34)	<0.001	1.26 (1.17 to 1.36)	<0.001
Household member has chronic illness:								
No	7413 (20.9)	28 032 (79.1)	Reference	-	Reference	-	Reference	-
Yes	1321 (18.9)	5663 (81.1)	1.09 (1.02 to 1.17)	0.01	1.02 (0.95 to 1.10)	0.58	1.03 (0.96 to 1.11)	0.37
Employed:								
No	3741 (18.9)	16 049 (81.1)	Reference	-	Reference	-	Reference	-
Yes	5157 (22.4)	17 904 (77.6)	0.85 (0.81 to 0.89)	<0.001	1.20 (1.13 to 1.28)	<0.001	1.14 (1.07 to 1.21)	<0.001
Highest earner works in a manual occupation:								
No	6048 (19.7)	24 633 (80.3)	Reference	-	Reference	-	-	-
Yes	2876 (24.3)	8959 (75.7)	0.79 (0.74 to 0.83)	<0.001	0.88 (0.83 to 0.94)	<0.001	-	-
Socioeconomic grade								
ABC1 (high)	4515 (18.7)	19 572 (81.3)	Reference	-	-	-	Reference	-
C2DE	4409 (23.9)	14 020 (76.1)	0.75 (0.71 to 0.79)	<0.001	-	-	0.79 (0.74 to 0.83)	<0.001
Index of multiple deprivation fourth:								
1st (least deprived)	1580 (16.5)	8004 (83.5)	1.69 (1.57 to 1.83)	<0.001	1.39 (1.28 to 1.52)	<0.001	1.35 (1.23 to 1.47)	<0.001
2nd	2042 (19.3)	8544 (80.7)	1.37 (1.28 to 1.47)	<0.001	1.15 (1.06 to 1.24)	<0.001	1.12 (1.04 to 1.21)	0.004
3rd	2600 (22.6)	8926 (77.4)	1.13 (1.05 to 1.21)	<0.001	1.03 (0.96 to 1.11)	0.39	1.02 (0.95 to 1.10)	0.61
4th (most deprived)	2916 (24.8)	8825 (75.2)	Reference	-	Reference	-	Reference	-
Overall	-	-	χ^2^(3)=206.9	<0.001	χ^2^(3)=66.9	<0.001	χ^2^(3)=54.3	<0.001
Highest educational or professional qualification:								
GCSE, vocational, A level, or no formal qualifications	6313 (21.8)	22 670 (78.2)	Reference	-	Reference	-	Reference	-
Degree or higher (bachelors, masters, or PhD)	2825 (19.5)	11 629 (80.5)	1.15 (1.09 to 1.21)	<0.001	1.20 (1.13 to 1.28)	<0.001	1.16 (1.09 to 1.23)	<0.001
Ethnicity:								
White British	7360 (20.0)	29 472 (80.0)	Reference	-	Reference	-	Reference	-
White other	663 (24.4)	2053 (75.6)	0.78 (0.71 to 0.87)	<0.001	0.97 (0.86 to 1.08)	0.54	0.96 (0.86 to 1.08)	0.53
Mixed	227 (26.7)	623 (73.3)	0.66 (0.56 to 0.77)	<0.001	0.81 (0.68 to 0.97)	0.02	0.81 (0.68 to 0.97)	0.02
Asian or Asian British	483 (26.2)	1364 (73.8)	0.75 (0.66 to 0.84)	<0.001	0.98 (0.86 to 1.12)	0.75	0.97 (0.85 to 1.11)	0.64
Black or black British	232 (29.8)	546 (70.2)	0.55 (0.46 to 0.65)	<0.001	0.75 (0.62 to 0.91)	0.003	0.74 (0.61 to 0.90)	0.002
Arab or other	47 (28.8)	116 (71.2)	0.60 (0.41 to 0.87)	0.01	0.67 (0.43 to 1.02)	0.06	0.65 (0.43 to 1.00)	0.05
Don’t know or prefer not to say	126 (50.2)	125 (49.8)	0.33 (0.25 to 0.43)	<0.001	0.31 (0.22 to 0.44)	<0.001	0.31 (0.22 to 0.44)	<0.001
Overall	-	-	χ^2^(6)=166.0	<0.001	χ^2^(6)=56.1	<0.001	χ^2^(6)=56.7	<0.001
Live alone:								
No	7209 (20.8)	27 425 (79.2)	Reference	-	Reference	-	Reference	-
Yes	1929 (21.9)	6874 (78.1)	0.93 (0.88 to 1.00)	0.04	0.81 (0.76 to 0.88)	<0.001	0.83 (0.77 to 0.90)	<0.001
Work in key sector:								
No	2759 (24.0)	8737 (76.0)	Reference	-	Reference	-	Reference	-
Yes	2981 (21.6)	10 846 (78.4)	1.12 (1.06 to 1.20)	<0.001	1.11 (1.04 to 1.19)	0.002	1.11 (1.04 to 1.19)	0.003
Self-employed‡								
No	4778 (22.3)	16 689 (77.7)	Reference	-	Reference	-	Reference	-
Yes	379 (23.8)	1215 (76.2)	0.94 (0.83 to 1.07)	0.34	0.85 (0.74 to 0.97)	0.01	0.85 (0.74 to 0.97)	0.02
Marital status:								
Single, separated, divorced, or widowed	4067 (23.8)	12 990 (76.2)	Reference	-	Reference	-	Reference	-
Married or partnered	4921 (18.9)	21 053 (81.1)	1.31 (1.25 to 1.38)	<0.001	1.19 (1.11 to 1.28)	<0.001	1.18 (1.10 to 1.26)	<0.001
Ever had covid-19:								
Think not	7958 (20.9)	30 056 (79.1)	Reference	-	Reference	-	Reference	-
Think so, or confirmed	1180 (21.8)	4243 (78.2)	0.97 (0.91 to 1.05)	0.47	1.11 (1.02 to 1.20)	0.01	1.11 (1.02 to 1.20)	0.01
Hardship§	n=8660; mean 8.2 (SD 2.7)	n=33 075; mean 7.6 (SD 2.9)	0.936 (0.928 to 0.944)	<0.001	0.97 (0.96 to 0.98)	<0.001	0.97 (0.96 to 0.98)	<0.001

SD=standard deviation; CI=confidence interval. For continuous variables, odds ratios represent a one unit increase in the explanatory variable, apart from for age, when odds ratios represent a 10 year increase in age.

* Adjusted for survey wave, region, sex, age (raw and quadratic term), dependent child in household, clinically vulnerable to covid-19, household member with a chronic illness, employment status, highest earner works in a manual occupation, index of multiple deprivation, highest educational or professional qualification, ethnicity, and living alone.

† Adjusted for survey wave, region, sex, age (raw and quadratic term), dependent child in household, clinically vulnerable to covid-19, household member with a chronic illness, employment status, socioeconomic grade, index of multiple deprivation, highest educational or professional qualification, ethnicity, and living alone.

‡ Not adjusted for employment status as by definition all people who were asked whether they were self-employed were working.

§ From 3 (least hardship) to 15 (most hardship).

In sensitivity analyses adjusting for socioeconomic grade, although the difference by index of multiple deprivation was still significant, the association with the second fourth of deprivation no longer reached our threshold for significance (table 3 and supplementary file). No other differences were observed.

When data from 1 June 2020 to 27 January 2021 (wave 19 to 42) were combined, the most commonly reported reasons for not intending to share details of close contacts were not knowing if data would be secure and confidential (14.6%), thinking that the contact tracing system was not accurate and reliable (13.9%), and not knowing what would happen to the data (13.0%; see supplementary file).

## Discussion

As in other countries, the test, trace, and isolate system should be a cornerstone of the UK’s public health strategy for coping with the covid-19 pandemic.^1^ Its success relies on adherence to multiple behaviours.^8^ Our data suggest that self-reported rates of full adherence to isolating and testing are low, as are rates of recognition of the main symptoms of covid-19. Rates of intended isolation and testing are higher. The percentage of people who intend to report details of close contacts is also high. However, given that the gap between intended and actual behaviour is a general phenomenon,^28^ the percentage of people who do share details of all close contacts after receiving a positive test result is likely to be lower. With such low rates for symptom recognition, testing, and full self-isolation, the effectiveness of the current form of the UK’s test, trace, and isolate system is limited.

### Comparison with other studies

These low rates of symptom recognition are comparable to those found in other UK research.^9^ ^14^ ^29^ Recognising that symptoms might be indicative of covid-19 is a first step in the chain that leads to isolation when required. Greater work to understand why symptom recognition remains low and how to boost it further is important. Further emphasis on specific symptoms might be necessary. In the UK, mass testing of people without symptoms has been introduced in areas with high case prevalence. This removes the need for recognition of symptoms of covid-19, although concerns remain about the effectiveness of mass testing.^30^

Our data suggest that the percentage of people with covid-19 symptoms who request a test has increased over time. Although media attention on testing capacity in the UK was considerable, our data show that increases in capacity were not reflected in the percentage of people with symptoms who requested a test. Despite increasing rates of tests being requested, other research corroborates our finding of a shortfall between national estimates of covid-19 and uptake of antigen tests. Our estimates of the percentage of people requesting a test (eg, 24.5% in late October 2020) is lower than the estimate that can be derived by dividing the number of daily cases identified in the community by NHS Test and Trace^31^ by the estimated daily incidence recorded by the ONS (32-52% for late October).^32^ Estimates of prevalence of covid-19 in England from the REACT 1 study (a large scale national study investigating the prevalence of covid-19)^33^ suggest a greater shortfall. This might be accounted for by different sample biases, the probable inclusion of people in our sample with an obvious, non-covid-19 explanation for their symptoms, and the probable inclusion of people without symptoms in the NHS Test and Trace data.

When we accounted for duration of isolation, the rates of people adhering to self-isolation were about 20 percentage points higher than those when we did not account for duration of isolation. Few associations reached significance after a Bonferroni correction was applied owing to the inclusion of fewer survey waves and the resulting smaller sample sizes, although some variables showed similar effects to the first analysis. When accounting for duration of isolation, our estimates of adherence to self-isolation were higher than previous data found by our team from May 2020, which suggested that only 25% of people with covid-19 symptoms in their household had not left home in the previous 24 hours.^10^ Our rates of self-reported adherence are similar to those referred to in a brief note about a study conducted by the Department of Health and Social Care, which found that 59% of people who were asked to isolate by NHS Test and Trace reported not leaving their home.^34^

In the latest available wave of data collection (25-27 January 2021), 82% of people intended to share details of close contacts if asked to by NHS Test and Trace. According to NHS Test and Trace, 25% of people who test positive for covid-19 do not provide details of any close contacts, suggesting a slight degree of underreporting.^35^

The UK’s implementation of test, trace, and isolate differs from that of other countries.^36^ ^37^ Although our study focused on behaviour in the UK, the associations found might be generalisable to other countries. For example, the percentages of people with symptoms who self-isolated in our study were comparable to those reported by a similar study in the Netherlands.^38^ However, higher rates of testing in the Netherlands means that more people with covid-19 are likely to be identified and therefore the contact tracing system might work more effectively than in the UK. Other factors that might improve the effectiveness of a test, trace, and isolate system include reducing delays between requesting a test and receiving the results.^39^

### Implications of the findings

It has been proposed that better financial and practical support might improve rates of adherence to test, trace and isolate behaviours.^40^ The importance of support is reflected in the associations we observed in the data, with financial hardship, index of multiple deprivation, lower socioeconomic status, and having a dependent child in the household showing a pattern of associations with lower adherence to full self-isolation, not requesting a test, and poorer symptom recognition. Evidence from other countries also suggests an association between greater financial hardship and poorer self-isolation.^41^ The disproportionate impact of the pandemic on people from lower socioeconomic backgrounds and with carer responsibilities has been well documented.^42^ ^43^ Behaviour reflects opportunities and capabilities as well as motivation: people need help to achieve their intentions. While intentions to engage in test, trace, and isolate behaviours are high, a greater focus on financial and practical support is likely to enable more people to translate their intentions into behaviour.^11^

Males and younger people were less likely to engage with testing, self-isolate, and intend to provide details of close contacts. This might reflect poorer health literacy in males, and, among younger people, a greater desire to be active and have contact with peer groups.^44^ People who believed they had already experienced covid-19 were less likely to fully self-isolate when symptomatic. Reduced adherence to social distancing measures has also been reported in this group.^14^ Other research has found an association between higher education and poorer adherence to UK government guidance.^45^ Working in a key sector was also associated with not fully self-isolating. This might be because key workers have a greater financial need to work, feel a greater social pressure to attend work, or are less likely to be able to work from home.^46^ Key workers and people from minority ethnic backgrounds were less likely to identify common symptoms of covid-19. Engagement and tailored communications with these groups is likely to improve knowledge of symptoms.

### Strengths and limitations of this study

Strengths of this study include the large sample sizes, allowing us to investigate uncommon behaviours and to examine uptake of protective behaviours and knowledge over time. We used quota sampling to ensure that participant characteristics were representative of the UK adult population. Although we cannot be sure that survey respondents are representative of the general population,^47^ ^48^ online quota sampling is a pragmatic approach when a large, demographically representative sample needs to be obtained in a short time frame during a crisis.^15^ ^49^ Odds ratios should thus be interpreted with some caution. However, issues about representativeness of participants are unlikely to undermine the interpretation of the study. Data were self-reported and so could have been influenced by social desirability and recall gaps and bias. Social desirability might have become particularly important after September 2020, when adherence to self-isolation became enforceable under law. The anonymity of our surveys should have mitigated this, however. As data are cross sectional, we cannot infer causality.

The nature of an online poll might raise questions as to the level of attention participants pay to their responses. While this is generally no different to any other questionnaire study, the possibility of “professional respondents” is a particular problem in online samples.^50^ Assuming such respondents introduce random error into the data, the impact on most items is limited but could become problematic in small subsamples.

Our study was prone to other specific methodological limitations. For symptom identification, we asked participants about the common symptoms of covid-19 and classified responses as symptoms being correctly identified if they selected symptoms promoted to members of the UK public as the “main” symptoms of covid-19 in government guidance.^20^ This decision was taken to enable measurement of adherence to policy. However, we recognise that other common symptoms of covid-19 exist (eg, fatigue, headache), which we did not include as being correct. For self-isolation, although we asked participants if they had left home at all since developing covid-19 symptoms, technically it is permissible to leave home under some circumstances, including to attend a medical appointment, to get a test, or when a test result is negative. In our sample, 15.0% of people reported leaving home for a medical need other than covid-19. Therefore, low rates of full self-isolation cannot be explained by permitted outings alone. People receiving a positive covid-19 test result might be more likely to adhere to self-isolation guidance,^51^ especially following legal enforcement of self-isolation on 20 September 2020.^52^ However, too few people in the sample reported that their test result indicated they had covid-19 to be able to conduct any meaningful analyses. For intention to share details of close contacts, the survey item did not differentiate between household and external contacts.

Although we had a large overall sample size, numbers of participants included in analyses of full self-isolation and requesting a test were smaller, resulting in small cell counts for some analyses. For these variables (region and ethnicity), we used different groupings. For region, we grouped together participants from Scotland, Wales, and Northern Ireland. Test, trace, and isolate systems in the four UK nations are managed locally and problems with the system in one nation might not be observed in other nations. For ethnicity, we grouped together black people, Asian people, and people of mixed ethnicity. This might have obscured differences between ethnic groups.^53^

### Conclusions

The spread of covid-19 presents many challenges, not least asymptomatic spread.^54^ ^55^ Test, trace, and isolate will never be a complete solution and will be more effective when the reproduction rate of the virus is low.^56^ However, it remains an important component of the UK’s national response. For the test, trace, and isolate system in the UK to succeed, people must recognise the main symptoms of covid-19 and be able and motivated to self-isolate, request a test, and share details of their close contacts when required. Our results indicate that about half of people know the symptoms of covid-19, and that adherence to each stage of test, trace, and isolate is low but improving slowly. Policies that support people financially and practically, and improving communication about the testing system, will be key to increasing uptake both in the UK and internationally.

**What is already known on this topic**

Test, trace, and isolate systems are one of the cornerstones of a national covid-19 recovery strategy
The success of any test, trace, and isolate system relies on people adhering to isolation if they have symptoms, getting a test if symptoms are present, and passing on details of close contacts if infection is confirmed

**What this study adds**

Self-reported adherence to test, trace, and self-isolate behaviours in the UK population is low; intention to carry out these behaviours is higher
In the UK, identification of covid-19 symptoms is low
Continued improvements to support are likely to be crucial in encouraging more people to adhere to test, trace, and self-isolate behaviours

## Data Availability

No additional data available.

## Web extra

Extra material supplied by authors

## Notes

The authors affirm that the manuscript is an honest, accurate, and transparent account of the study being reported; that no important aspects of the study have been omitted; and that any discrepancies from the study as originally planned have been explained.

Dissemination to participants and related patient and public communities: Dissemination of survey results to participants is not possible owing to the anonymous nature of data collection.

Provenance and peer review: Not commissioned; externally peer reviewed.
