# Adherence to the test, trace, and isolate system in the UK: results from 37 nationally representative surveys

**DOI:** 10.1136/bmj.o2008

**Published:** 2022-08-16

Following publication of this paper by Smith and colleagues (*BMJ* 2021;372:n608, doi:10.1136/bmj.n608, published 31 March 2021), and as part of ongoing work with the Department of Health and Social Care, the authors have become aware of two survey deployment and data collection errors in the COVID-19 Rapid Survey of Adherence to Interventions and Responses (CORSAIR) study. Specifically, an error in the assignment of the responder ID prevented correct identification of repeat respondents. In addition, a data management error led to the incorrect categorisation of respondents’ socioeconomic grade.

The authors have revised the manuscript to ensure accuracy and reanalysed the data after correcting these errors to:

• Update numbers of responses and participants included in analyses, using corrected unique participant identifiers. 

• Re-run generalised estimating equation analyses, using corrected unique participant identifiers.

• Conduct a sensitivity analysis for generalised estimating equations, using socioeconomic grade.

More details can be found in the authors’ rapid response.

The authors, handling editor, and head of research at *The BMJ* have reviewed the authors’ explanation, and the corrected work. All parties agree that the main findings of the work remain reliable and that the errors/clarifications have arisen in honest circumstances. Therefore, the authors and publisher have corrected the work.

A clean and corrected version is displayed on bmj.com. For transparency, the previous version with tracked changes is appended.

## Web extra

Web extra as supplied by authors

Supplementary information: pdf of paper showing revisions in tracked changes

