# Supplementary material for: Adherence to the test, trace, and isolate system in the UK: results from 37 nationally representative surveys
Source: BMJ. 2021 Mar 31;372:n608. doi: 10.1136/bmj.n608 (PMC8010268; doi:10.1136/bmj.n608)
Supplement: Supplementary file 1 — Supplementary information: additional tables and figures [file smil063382.ww1.pdf]

Supplementary materials. Details of numbers of individual analyses, and participants and responses included.

Outcome:	Analyses	Waves included	Participant inclusion criteria	Number of responses, from individual participants included in analyses
Identification of COVID-19 symptoms	GEEs using an exchangeable correlation structure	26 May 2020 to 27 January 2021 (waves 18 to 42), excluding data collected 26 to 28 October 2020 (wave 31)*	All participants	48,168 responses, from 34,752 participants
Full-self-isolation when symptomatic, duration adjusted	GEEs using an exchangeable correlation structure	26 October 2020 to 27 January 2021 (waves 31 to 42), excluding data collected 26 to 28 October 2020 (wave 31)*	Only those reporting cardinal COVID-19 symptoms† in the last seven days, excluding those with a negative COVID-19 test	1102 responses, from 1066 participants
Full self-isolation when symptomatic	GEEs using an exchangeable correlation structure	14 April 2020 to 27 January 2021 (waves 12 to 42), excluding data collected 26 to 28 October 2020 (wave 31)*	Only those reporting cardinal COVID-19 symptoms† in the last seven days, excluding those with a negative COVID-19 test	3397 responses, from 2967 participants
Requesting a test when symptomatic	GEEs using an exchangeable correlation structure	26 May 2020 to 27 January 2021 (waves 18 to 42), excluding data collected 26 to 28 October 2020 (wave 31)*	Only those reporting cardinal COVID-19 symptoms† in the last seven days	2920 responses, from 2612 participants
Sharing details of close contacts	GEEs using an exchangeable correlation structure	1 June 2020 to 27 January 2021 (waves 19 to 42), excluding data collected 26 to 28 October 2020 (wave 31)*	Participants who had not reported cardinal COVID-19 symptoms† in the last seven days	43,437 responses, from 31,799 participants

* Due to an error in collecting data about chronic illness on 26 to 28 October 2020 (wave 31), these data were excluded from analyses

† High temperature / fever, cough, or loss or change of sense of smell or taste

Supplementary materials. Excluding people who tested negative for covid-19 from self-isolation analyses

We used data from different questions to identify participants who tested negative for covid-19. In data collected between 1 June and 5 August 2020 (waves 19 to 26), participants who reported they had requested a test after developing covid-19 symptoms were asked to indicate their test result or if they were still waiting for results. In data collected from 7 December 2020 (wave 36 onwards), all participants were asked if they had been tested for covid-19 in the last seven days, and if so, what the results of their test were. In data collected from 11 January 2021 (wave 41 onwards), having received a negative test was included in the list of reasons why you had left home since developing symptoms (only shown to participants who reported having covid-19 symptoms and who reported having requested a test). In all waves, participants with covid-19 symptoms could specify “other” reasons for having left home.

Supplementary materials. Coding of variable indicating that the highest earner in the household is a manual worker and socio-economic grade

**Which of the following best describes the occupation of the member of your household with the largest income (the chief income earner)? Please select one answer**<br>Please indicate to which occupational group the Chief Income Earner in your household belongs, or which group fits best.<br>The Chief Income Earner is the person in your household with the largest income.<br>If the Chief Income Earner is retired and has an occupational pension please answer for their most recent occupation.<br>If the Chief Income Earner is not in paid employment but has been out of work for less than 6 months, please answer for their most recent occupation	Highest earner in the household is a manual worker	Socio-economic grade
**Semi or unskilled manual work** (e.g. Manual workers, all apprentices to skilled trades, Caretaker, Park keeper, non-HGV driver, shop assistant)	Yes	C2DE
**Skilled manual worker** *(e.g. Skilled Bricklayer, Carpenter, Plumber, Painter, Bus/ Ambulance Driver, HGV driver, AA patrolman, pub/bar worker, etc.)*	Yes	C2DE
**Supervisory or clerical/ junior managerial/ professional/administrative** *(e.g. Office worker, Student Doctor, Foreman with 25+ employees, salesperson, etc.)*	No	ABC1
**Intermediate managerial/ professional/ administrative** *(e.g. Newly qualified (under 3 years) doctor, Solicitor, Board director small organisation, middle manager in large organisation, principle officer in civil service/local government)*	No	ABC1
**Higher managerial/ professional/ administrative** *(e.g. Established doctor, Solicitor, Board Director in a large organisation (200+ employees, top level civil servant/public service employee)*	No	ABC1
**Student**	No	ABC1
**Casual worker – not in permanent employment**	No	C2DE
**Housewife/ Homemaker**	No	C2DE
**Retired and living on state pension**	No	C2DE
**Unemployed or not working due to long-term sickness**	No	C2DE
**Full-time carer of another household member**	No	C2DE
Other	Missing	Missing

Supplementary materials. Endorsement of common symptoms of COVID-19

Can you tell us what you think the most common symptoms of coronavirus are? Please select up to 5	26 May 2020 to 27 January 2021, n†	%	25 to 27 January 2021, n‡	%
Cough	37,336	73.8	1516	75.5
High temperature / fever	36,560	72.2	1383	68.9
Loss of sense of smell	33,280	65.7	1424	71.0
Loss of taste	32,211	63.6	1422	70.9
Shortness of breath / difficulty breathing	27,064	53.5	1142	56.9
Flu-like symptoms	14,351	28.4	483	24.1
Sore throat	8,126	16.1	275	13.7
Feeling tired or having low energy	7,986	15.8	325	16.2
Headaches	5,028	9.9	214	10.7
Loss of appetite	4,086	8.1	164	8.2
Aches and pains	2,855	5.6	123	6.1
Chest pain	2,516	5.0	86	4.3
Sneezing	2,382	4.7	53	2.6
Chills / shivering	2,225	4.4	98	4.9
Nausea / feeling sick	1,550	3.1	73	3.6
Diarrhoea	1,482	2.9	51	2.5
Runny nose	1,449	2.9	45	2.2
Pain in your arms, legs or joints	1,126	2.2	42	2.1
Blocked nose	870	1.7	39	1.9
Dizziness	879	1.7	38	1.9
Vomiting	787	1.6	36	1.8
Feeling your heart pound or race	598	1.2	23	1.1
Stomach ache	538	1.1	21	1.0
Trouble sleeping	516	1.0	16	0.8
Back pain	511	1.0	21	1.0
Fainting spells	440	0.9	23	1.1
Don’t know (single code)	1,189	2.3	33	1.6

† Baseline n=50,617.
‡ Baseline n=2,007.

Supplementary materials. Lowess plots showing associations between outcomes and age.

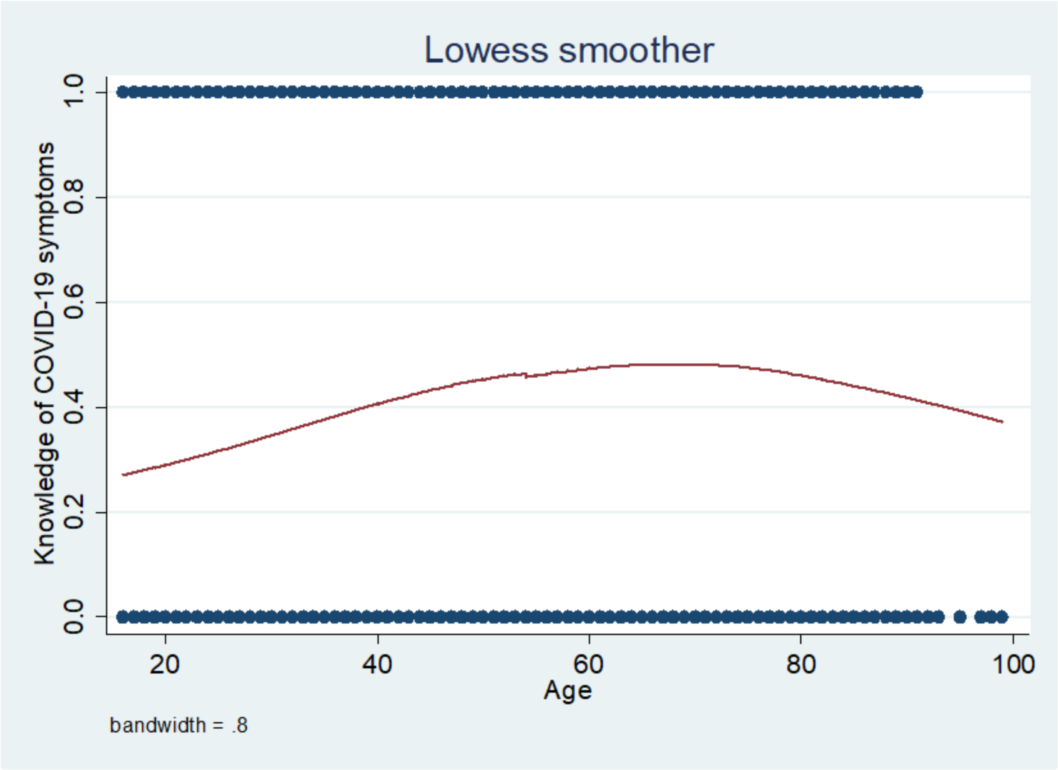

Figure 1. Symptom identification and age.

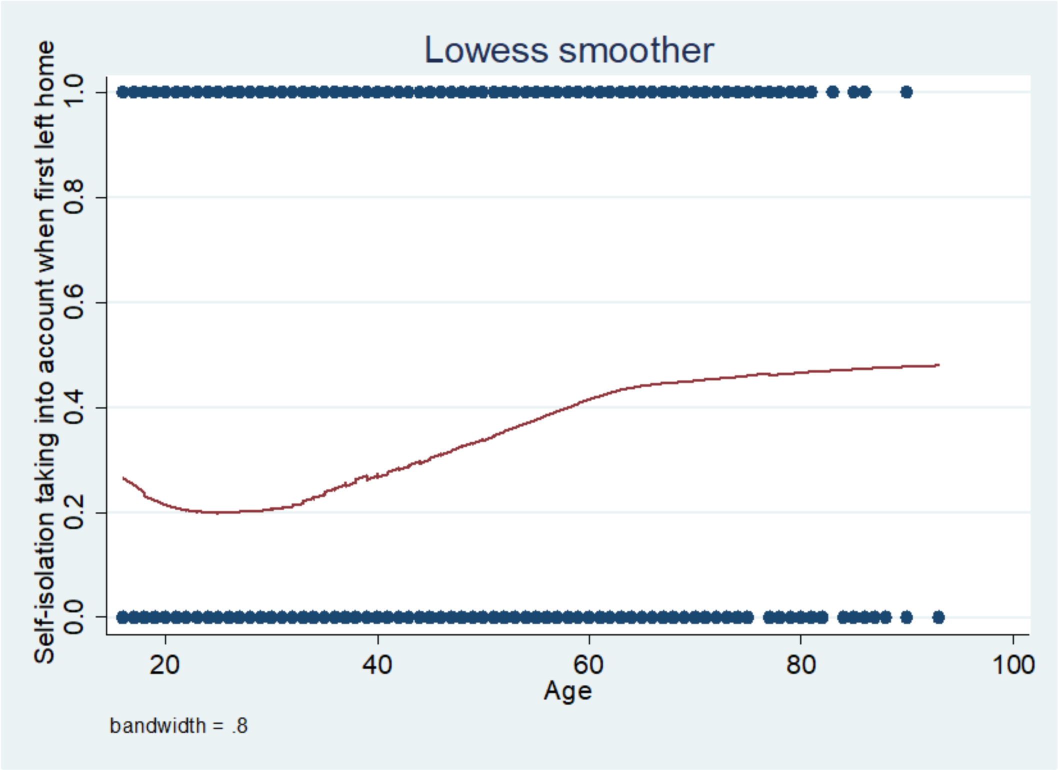

Figure 2. Full self-isolation accounting for duration of isolation and age.

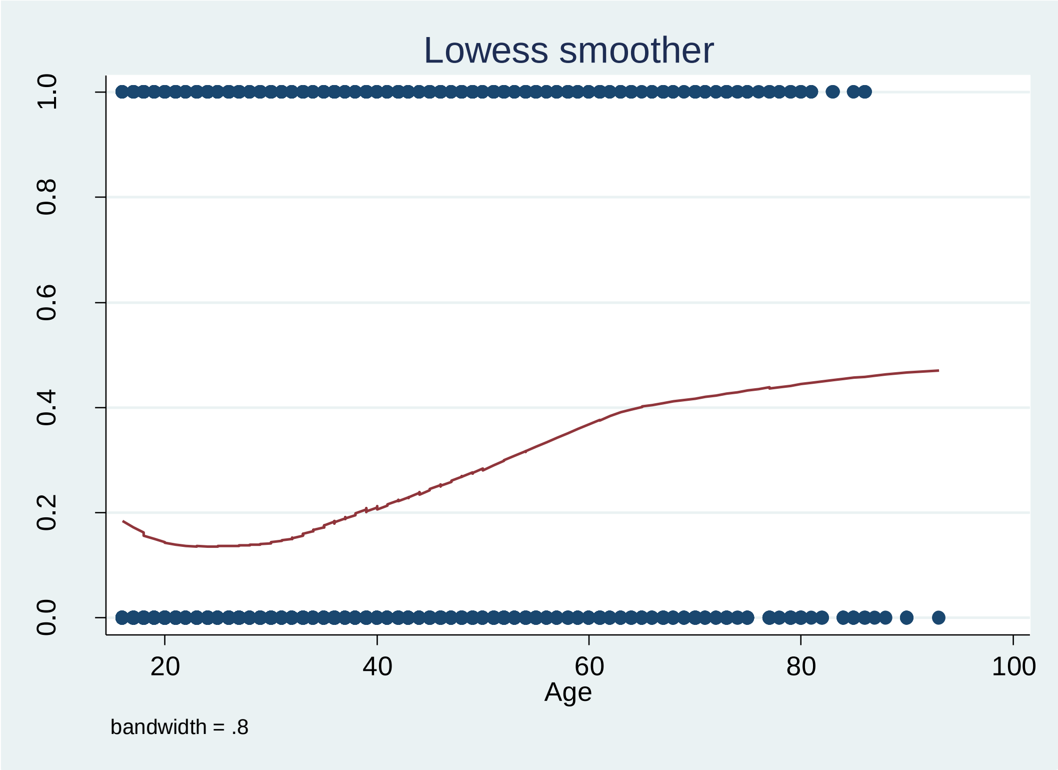

Figure 3. Full self-isolation and age.

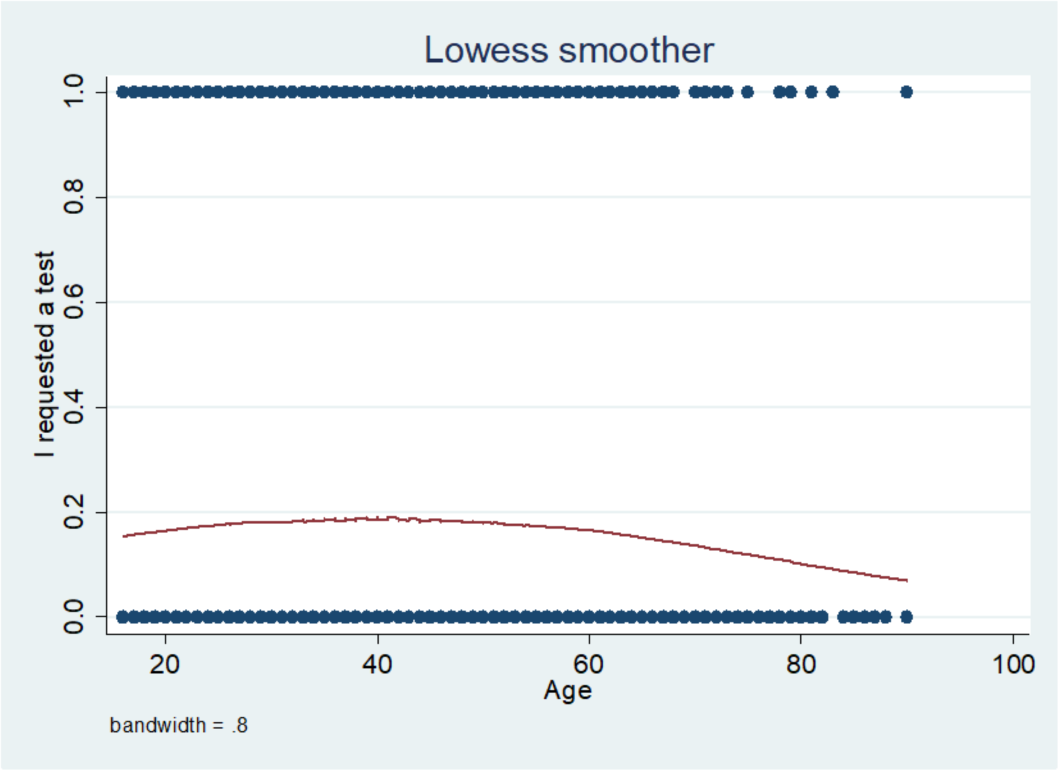

Figure 4. Requesting a test after developing symptoms of covid-19 and age.

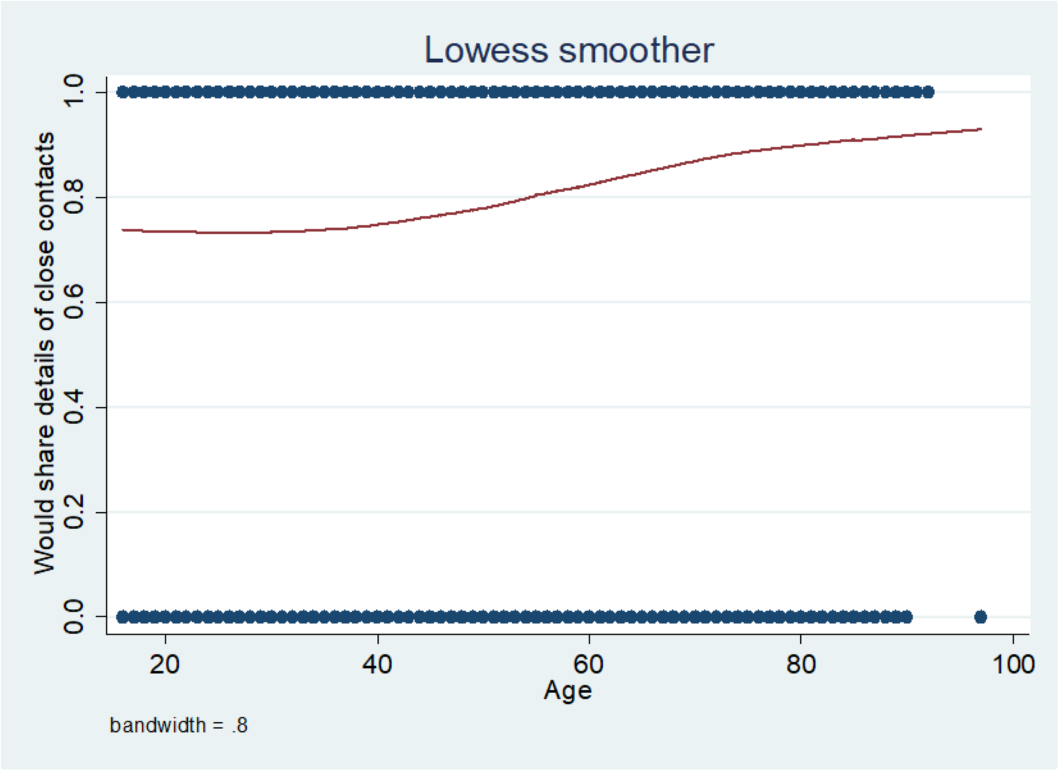

Figure 5. Intention to share details of your close contacts with the NHS contact tracing service and age.

Supplementary materials. Associations between outcomes and survey wave and region.

Table 1. Symptom identification and associations with survey wave and region. Bolding indicates findings significant at a $p<0.001$.

Participant characteristics	Level	Did not identify cough, high temperature / fever, and loss of sense of smell or taste n=23,440	Identified cough, high temperature / fever, and loss of sense of smell or taste n=24,728	Odds ratio (95% CI) for correctly identifying symptoms	p-value	Adjusted odds ratio (95% CI) for correctly identifying symptoms*	p-value	Adjusted odds ratio (95% CI) for correctly identifying symptoms†	p-value
Survey wave	26 to 27 May 2020 (wave 18)	1033 (52.2)	947 (47.8)	Reference	-	Reference	-	Reference	-
	1 to 3 June 2020 (wave 19)	970 (49.0)	1009 (51.0)	1.06 (0.94 to 1.19)	0.33	1.05 (0.93 to 1.19)	0.44	1.05 (0.93 to 1.19)	0.39
	8 to 10 June 2020 (wave 20)	968 (49.0)	1009 (51.0)	1.03 (0.92 to 1.16)	0.57	1.02 (0.90 to 1.16)	0.75	1.02 (0.90 to 1.16)	0.74
	15 to 17 June 2020 (wave 21)	1014 (51.0)	974 (49.0)	0.92 (0.82 to 1.03)	0.16	0.92 (0.81 to 1.04)	0.19	0.93 (0.82 to 1.05)	0.22
	22 to 24 June 2020 (wave 22)	1016 (51.2)	970 (48.8)	0.94 (0.83 to 1.06)	0.28	0.92 (0.81 to 1.05)	0.22	0.93 (0.81 to 1.05)	0.24
	29 June to 1 July 2020 (wave 23)	1009 (50.9)	972 (49.1)	0.95 (0.85 to 1.07)	0.40	0.92 (0.81 to 1.04)	0.16	0.92 (0.81 to 1.04)	0.18
	6 to 8 July 2020 (wave 24)	1060 (53.2)	934 (46.8)	0.86 (0.77 to 0.97)	0.01	0.84 (0.74 to 0.95)	0.01	0.84 (0.75 to 0.95)	0.01
	20 to 22 July 2020 (wave 25)	1035 (51.7)	967 (48.3)	0.93 (0.83 to 1.05)	0.24	0.94 (0.83 to 1.07)	0.36	0.95 (0.84 to 1.07)	0.39
	3 to 5 August 2020 (wave 26)	1004 (50.5)	987 (49.5)	0.93 (0.83 to 1.05)	0.24	0.95 (0.84 to 1.08)	0.42	0.95 (0.84 to 1.08)	0.45
	1 to 2 September 2020 (wave 27)	1058 (52.6)	954 (47.4)	0.89 (0.79 to 1.01)	0.06	0.88 (0.77 to 1.00)	0.05	0.89 (0.78 to 1.01)	0.06
	14 to 16 September 2020 (wave 28)	975 (49.2)	1005 (50.8)	1.00 (0.89 to 1.12)	0.96	0.98 (0.87 to 1.12)	0.81	0.99 (0.87 to 1.12)	0.86
	28 to 30 September 2020 (wave 29)	903 (44.4)	1130 (55.6)	1.19 (1.06 to 1.34)	0.004	1.17 (1.03 to 1.33)	0.01	1.18 (1.04 to 1.34)	0.01
	12 to 14 October 2020 (wave 30)	932 (45.2)	1131 (54.8)	1.16 (1.03 to 1.30)	0.02	1.11 (0.98 to 1.26)	0.11	1.12 (0.99 to 1.27)	0.08
	9 to 11 November 2020 (wave 32)	925 (45.8)	1096 (54.2)	1.13 (1.00 to 1.27)	0.05	1.12 (0.98 to 1.27)	0.09	1.12 (0.99 to 1.27)	0.08
	16 to 18 November 2020 (wave 33)	972 (47.5)	1074 (52.5)	1.08 (0.96 to 1.21)	0.22	1.06 (0.94 to 1.21)	0.35	1.07 (0.94 to 1.22)	0.29
	23 to 25 November 2020 (wave 34)	957 (47.6)	1055 (52.4)	1.03 (0.92 to 1.16)	0.57	0.99 (0.87 to 1.12)	0.85	0.99 (0.87 to 1.13)	0.90
	30 November to 2 December 2020 (wave 35)	979 (47.9)	1063 (52.1)	1.05 (0.94 to 1.19)	0.37	1.08 (0.95 to 1.22)	0.23	1.08 (0.95 to 1.23)	0.22
	7 to 9 December 2020 (wave 36)	963 (46.0)	1130 (54.0)	1.12 (1.00 to 1.26)	0.05	1.08 (0.95 to 1.22)	0.25	1.08 (0.96 to 1.23)	0.21
	14 to 16 December 2020 (wave 37)	945 (46.9)	1071 (53.1)	1.08 (0.96 to 1.21)	0.21	1.05 (0.92 to 1.18)	0.49	1.05 (0.93 to 1.19)	0.45
	21 to 23 December 2020 (wave 38)	950 (47.2)	1061 (52.8)	1.05 (0.94 to 1.18)	0.40	1.03 (0.91 to 1.17)	0.61	1.04 (0.92 to 1.18)	0.56
	28 to 30 December 2020 (wave 39)	914 (46.8)	1037 (53.2)	1.10 (0.98 to 1.24)	0.12	1.03 (0.91 to 1.17)	0.62	1.04 (0.91 to 1.18)	0.58
	4 to 6 January 2021 (wave 40)	918 (45.6)	1094 (54.4)	1.13 (1.00 to 1.27)	0.05	1.14 (1.01 to 1.30)	0.04	1.15 (1.01 to 1.31)	0.04
	11 to 13 January 2021 (wave 41)	953 (47.7)	1044 (52.3)	1.05 (0.93 to 1.18)	0.42	1.01 (0.89 to 1.15)	0.87	1.01 (0.89 to 1.15)	0.83
	25 to 27 January 2021 (wave 42)	987 (49.3)	1014 (50.7)	0.96 (0.86 to 1.09)	0.55	0.95 (0.84 to 1.08)	0.42	0.95 (0.84 to 1.08)	0.47
Region	East Midlands	1774 (46.6)	2036 (53.4)	Reference	-	Reference	-		
	East of England	2095 (45.0)	2556 (55.0)	1.03 (0.94 to 1.13)	0.55	1.00 (0.91 to 1.11)	0.94	1.01 (0.91 to 1.12)	0.84
	London	3440 (58.2)	2467 (41.8)	**0.60 (0.55 to 0.65)**	**<0.001**	**0.77 (0.70 to 0.85)**	**<0.001**	**0.76 (0.69 to 0.84)**	**<0.001**
	North East	1028 (47.2)	1151 (52.8)	0.95 (0.85 to 1.07)	0.41	0.97 (0.86 to 1.10)	0.63	0.97 (0.86 to 1.10)	0.65
	North West	2757 (50.8)	2669 (49.2)	**0.83 (0.76 to 0.91)**	**<0.001**	0.86 (0.78 to 0.95)	0.003	0.86 (0.78 to 0.95)	0.002
	Northern Ireland	394 (45.4)	474 (54.6)	1.05 (0.89 to 1.23)	0.58	1.06 (0.89 to 1.25)	0.53	1.05 (0.89 to 1.25)	0.54

	Scotland	1752 (46.9)	1985 (53.1)	0.96 (0.87 to 1.06)	0.38	0.95 (0.85 to 1.05)	0.32	0.95 (0.85 to 1.05)	0.31
	South East	2929 (45.7)	3480 (54.3)	1.01 (0.92 to 1.10)	0.83	0.98 (0.90 to 1.08)	0.71	0.98 (0.90 to 1.08)	0.71
	South West	1928 (46.3)	2232 (53.7)	0.97 (0.88 to 1.07)	0.59	0.94 (0.85 to 1.04)	0.26	0.94 (0.85 to 1.04)	0.25
	Wales	1079 (45.6)	1286 (54.4)	0.99 (0.89 to 1.11)	0.93	0.95 (0.84 to 1.07)	0.41	0.95 (0.85 to 1.07)	0.43
	West Midlands	2122 (49.7)	2147 (50.3)	0.86 (0.78 to 0.95)	0.002	0.95 (0.86 to 1.05)	0.32	0.95 (0.86 to 1.05)	0.31
	Yorkshire and the Humber	2142 (48.8)	2245 (51.2)	0.88 (0.80 to 0.97)	0.009	0.90 (0.81 to 0.99)	0.03	0.89 (0.81 to 0.99)	0.03

* Adjusting for survey wave, region, sex, age (raw and quadratic term), dependent child in the household, being clinically vulnerable to COVID-19, having a household member with a chronic illness, employment status, highest earner works in a manual occupation, index of multiple deprivation, highest educational or professional qualification, ethnicity, and living alone.

† Adjusting for survey wave, region, sex, age (raw and quadratic term), dependent child in the household, being clinically vulnerable to COVID-19, having a household member with a chronic illness, employment status, socioeconomic grade, index of multiple deprivation, highest educational or professional qualification, ethnicity, and living alone.

Table 2. Full self-isolation after developing symptoms of COVID-19 accounting for duration of isolation and associations with survey wave and region. Bolding indicates findings significant at a $p<0.001$.

Participant characteristics	Level	Did not fully self-isolate n=648	Fully self-isolated n=454	Odds ratio (95% CI) for fully self-isolating	p-value	Adjusted odds ratio (95% CI) for fully self-isolating *	p-value	Adjusted odds ratio (95% CI) for fully self-isolating †	p-value
Survey wave	9 to 11 November 2020 (wave 32)	78 (61.9)	48 (38.1)	Reference	-	Reference	-	Reference	-
	16 to 18 November 2020 (wave 33)	71 (55.5)	57 (44.5)	1.35 (0.82 to 2.23)	0.24	1.29 (0.74 to 2.24)	0.37	1.28 (0.74 to 2.24)	0.38
	23 to 25 November 2020 (wave 34)	66 (58.9)	46 (41.1)	1.16 (0.69 to 1.96)	0.57	1.07 (0.60 to 1.90)	0.82	1.07 (0.60 to 1.91)	0.82
	30 November to 2 December 2020 (wave 35)	76 (63.9)	43 (36.1)	0.94 (0.56 to 1.59)	0.83	0.77 (0.42 to 1.40)	0.39	0.77 (0.43 to 1.40)	0.40
	7 to 9 December 2020 (wave 36)	45 (51.1)	43 (48.9)	1.59 (0.91 to 2.76)	0.10	1.34 (0.73 to 2.44)	0.35	1.33 (0.73 to 2.43)	0.35
	14 to 16 December 2020 (wave 37)	59 (66.3)	30 (33.7)	0.90 (0.51 to 1.59)	0.72	0.74 (0.40 to 1.34)	0.32	0.74 (0.40 to 1.34)	0.32
	21 to 23 December 2020 (wave 38)	62 (67.4)	30 (32.6)	0.82 (0.47 to 1.44)	0.49	0.69 (0.37 to 1.29)	0.25	0.69 (0.37 to 1.29)	0.24
	28 to 30 December 2020 (wave 39)	48 (58.5)	34 (41.5)	1.19 (0.67 to 2.11)	0.55	1.05 (0.56 to 1.98)	0.87	1.06 (0.56 to 1.98)	0.87
	4 to 6 January 2021 (wave 40)	55 (52.9)	49 (47.1)	1.50 (0.88 to 2.53)	0.13	1.44 (0.80 to 2.57)	0.22	1.43 (0.80 to 2.57)	0.23
	11 to 13 January 2021 (wave 41)	48 (60.8)	31 (39.2)	1.12 (0.63 to 2.00)	0.71	0.87 (0.47 to 1.61)	0.66	0.87 (0.47 to 1.62)	0.66
	25 to 27 January 2021 (wave 42)	40 (48.2)	43 (51.8)	1.77 (1.02 to 3.06)	0.04	1.76 (0.96 to 3.21)	0.07	1.76 (0.97 to 3.21)	0.06
Region	East Midlands	41 (53.2)	36 (46.8)	Reference	-	Reference	-	Reference	-
	East of England	53 (58.2)	38 (41.8)	0.84 (0.45 to 1.56)	0.58	0.86 (0.44 to 1.70)	0.67	0.87 (0.44 to 1.71)	0.68
	London	134 (62.0)	82 (38.0)	0.71 (0.42 to 1.22)	0.22	0.79 (0.43 to 1.45)	0.45	0.79 (0.43 to 1.45)	0.45
	North (East and West)	103 (57.5)	76 (42.5)	0.87 (0.51 to 1.51)	0.63	1.03 (0.56 to 1.87)	0.93	1.03 (0.56 to 1.87)	0.93
	Northern Ireland / Scotland / Wales	72 (57.1)	54 (42.9)	0.91 (0.51 to 1.62)	0.75	0.93 (0.49 to 1.77)	0.82	0.93 (0.49 to 1.77)	0.82
	South East	72 (58.5)	51 (41.5)	0.83 (0.46 to 1.49)	0.53	0.90 (0.47 to 1.75)	0.76	0.90 (0.47 to 1.75)	0.76
	South West	52 (59.8)	35 (40.2)	0.82 (0.44 to 1.55)	0.54	0.69 (0.34 to 1.37)	0.29	0.68 (0.34 to 1.37)	0.29
	West Midlands	59 (57.8)	43 (42.2)	0.86 (0.47 to 1.57)	0.63	0.86 (0.45 to 1.66)	0.66	0.86 (0.45 to 1.66)	0.66
	Yorkshire and the Humber	62 (61.4)	39 (38.6)	0.74 (0.40 to 1.36)	0.33	0.77 (0.39 to 1.51)	0.44	0.77 (0.39 to 1.51)	0.44

* Adjusting for survey wave, region, sex, age (raw and quadratic), dependent child in the household, being clinically vulnerable to COVID-19, having a household member with a chronic illness, employment status, highest earner is a manual worker, index of multiple deprivation, highest educational or professional qualification, ethnicity, and living alone.

† Adjusting for survey wave, region, sex, age (raw and quadratic), dependent child in the household, being clinically vulnerable to COVID-19, having a household member with a chronic illness, employment status, socioeconomic grade, index of multiple deprivation, highest educational or professional qualification, ethnicity, and living alone.

Table 3. Full self-isolation after developing symptoms of COVID-19 and associations with survey wave and region. Bolding indicates findings significant at a *p*<0.001.

Participant characteristics	Level	Did not fully self-isolate n=2,717	Fully self-isolated n=680	Odds ratio (95% CI) for fully self-isolating	p-value	Adjusted odds ratio (95% CI) for fully self-isolating *	p-value	Adjusted odds ratio (95% CI) for fully self-isolating †	p-value
Survey wave	14 to 15 April 2020 (wave 12)	101 (80.8)	24 (19.2)	Reference	-	Reference	-	Reference	-
	20 to 22 April 2020 (wave 13)	112 (83.0)	23 (17.0)	0.89 (0.50 to 1.56)	0.68	1.34 (0.73 to 2.46)	0.35	1.34 (0.73 to 2.45)	0.34
	27 to 29 April (wave 14)	82 (80.4)	20 (19.6)	1.00 (0.54 to 1.83)	1.00	1.40 (0.70 to 2.78)	0.34	1.39 (0.70 to 2.75)	0.35
	4 to 6 May 2020 (wave 15)	88 (81.5)	20 (18.5)	0.94 (0.51 to 1.74)	0.85	0.96 (0.48 to 1.93)	0.90	0.96 (0.48 to 1.93)	0.91
	11 to 13 May (wave 16)	102 (84.3)	19 (15.7)	0.85 (0.48 to 1.52)	0.59	1.05 (0.56 to 1.96)	0.89	1.06 (0.57 to 1.98)	0.85
	18 to 20 May 2020 (wave 17)	79 (72.5)	30 (27.5)	1.42 (0.81 to 2.49)	0.22	1.17 (0.61 to 2.22)	0.64	1.18 (0.62 to 2.23)	0.62
	26 to 27 May 2020 (wave 18)	133 (78.7)	36 (21.3)	1.13 (0.67 to 1.90)	0.65	1.11 (0.62 to 2.01)	0.72	1.11 (0.62 to 2.00)	0.73
	1 to 3 June 2020 (wave 19)	97 (74.0)	34 (26.0)	1.37 (0.79 to 2.36)	0.26	1.53 (0.84 to 2.80)	0.17	1.52 (0.83 to 2.78)	0.17
	8 to 10 June 2020 (wave 20)	103 (79.8)	26 (20.2)	0.98 (0.55 to 1.72)	0.93	0.77 (0.40 to 1.46)	0.42	0.77 (0.40 to 1.45)	0.41
	15 to 17 June 2020 (wave 21)	101 (86.3)	16 (13.7)	0.64 (0.35 to 1.17)	0.15	0.64 (0.32 to 1.29)	0.21	0.64 (0.32 to 1.28)	0.21
	22 to 24 June 2020 (wave 22)	112 (88.2)	15 (11.8)	0.53 (0.27 to 1.02)	0.06	0.51 (0.25 to 1.04)	0.07	0.51 (0.25 to 1.04)	0.06
	29 June to 1 July 2020 (wave 23)	90 (80.4)	22 (19.6)	1.03 (0.58 to 1.82)	0.93	1.05 (0.56 to 1.97)	0.89	1.05 (0.56 to 1.98)	0.87
	6 to 8 July 2020 (wave 24)	92 (83.6)	18 (16.4)	0.94 (0.53 to 1.68)	0.84	1.07 (0.55 to 2.06)	0.85	1.10 (0.58 to 2.11)	0.77
	20 to 22 July 2020 (wave 25)	105 (85.4)	18 (14.6)	0.70 (0.38 to 1.27)	0.24	0.61 (0.31 to 1.19)	0.15	0.61 (0.31 to 1.18)	0.14
	3 to 5 August 2020 (wave 26)	85 (84.2)	16 (15.8)	0.69 (0.35 to 1.35)	0.28	0.70 (0.33 to 1.49)	0.36	0.70 (0.33 to 1.50)	0.36
	1 to 2 September 2020 (wave 27)	109 (81.3)	25 (18.7)	0.91 (0.50 to 1.64)	0.75	0.79 (0.40 to 1.58)	0.51	0.81 (0.40 to 1.61)	0.54
	14 to 16 September 2020 (wave 28)	102 (85.7)	17 (14.3)	0.64 (0.34 to 1.23)	0.18	0.47 (0.23 to 0.96)	0.04	0.49 (0.24 to 0.99)	0.05
	28 to 30 September 2020 (wave 29)	101 (87.8)	14 (12.2)	0.55 (0.28 to 1.07)	0.08	0.51 (0.24 to 1.09)	0.08	0.52 (0.25 to 1.11)	0.09
	12 to 14 October 2020 (wave 30)	79 (73.1)	29 (26.9)	1.29 (0.71 to 2.33)	0.40	1.12 (0.59 to 2.12)	0.74	1.13 (0.59 to 2.14)	0.72
	9 to 11 November 2020 (wave 32)	103 (81.7)	23 (18.3)	0.88 (0.48 to 1.60)	0.67	0.76 (0.39 to 1.48)	0.42	0.76 (0.39 to 1.48)	0.42
	16 to 18 November 2020 (wave 33)	99 (77.3)	29 (22.7)	1.18 (0.68 to 2.06)	0.55	1.14 (0.60 to 2.18)	0.69	1.15 (0.60 to 2.18)	0.67
	23 to 25 November 2020 (wave 34)	89 (79.5)	23 (20.5)	1.03 (0.57 to 1.86)	0.93	0.81 (0.42 to 1.57)	0.53	0.81 (0.42 to 1.59)	0.55
	30 November to 2 December 2020 (wave 35)	105 (88.2)	14 (11.8)	0.57 (0.30 to 1.09)	0.09	0.48 (0.23 to 1.00)	0.05	0.49 (0.23 to 1.02)	0.06
	7 to 9 December 2020 (wave 36)	58 (65.9)	30 (34.1)	1.97 (1.10 to 3.53)	0.02	1.58 (0.81 to 3.07)	0.18	1.55 (0.80 to 3.01)	0.19
	14 to 16 December 2020 (wave 37)	71 (79.8)	18 (20.2)	1.11 (0.59 to 2.08)	0.75	0.93 (0.47 to 1.83)	0.83	0.93 (0.47 to 1.82)	0.83
	21 to 23 December 2020 (wave 38)	72 (78.3)	20 (21.7)	1.06 (0.56 to 2.01)	0.87	1.03 (0.49 to 2.15)	0.94	1.03 (0.49 to 2.16)	0.94
	28 to 30 December 2020 (wave 39)	63 (76.8)	19 (23.2)	1.19 (0.62 to 2.31)	0.60	0.90 (0.43 to 1.88)	0.78	0.90 (0.43 to 1.89)	0.79
	4 to 6 January 2021 (wave 40)	71 (68.3)	33 (31.7)	1.86 (1.06 to 3.27)	0.03	1.77 (0.93 to 3.37)	0.08	1.76 (0.93 to 3.35)	0.08
	11 to 13 January 2021 (wave 41)	56 (70.9)	23 (29.1)	1.60 (0.84 to 3.04)	0.15	1.20 (0.58 to 2.51)	0.62	1.21 (0.58 to 2.52)	0.62

	25 to 27 January 2021 (wave 42)	57 (68.7)	26 (31.3)	1.70 (0.91 to 3.16)	0.10	1.85 (0.92 to 3.70)	0.08	1.87 (0.94 to 3.73)	0.08
Region	East Midlands	187 (79.6)	48 (20.4)	Reference	-	Reference	-	Reference	-
	East of England	219 (80.5)	53 (19.5)	0.97 (0.62 to 1.52)	0.90	1.04 (0.62 to 1.73)	0.88	1.07 (0.64 to 1.77)	0.81
	London	606 (86.9)	91 (13.1)	0.61 (0.41 to 0.91)	0.01	0.93 (0.59 to 1.48)	0.78	0.93 (0.59 to 1.48)	0.77
	North East	107 (74.8)	36 (25.2)	1.43 (0.86 to 2.37)	0.17	1.52 (0.84 to 2.76)	0.16	1.54 (0.85 to 2.78)	0.16
	North West	329 (81.8)	73 (18.2)	0.91 (0.60 to 1.37)	0.64	1.11 (0.70 to 1.76)	0.64	1.12 (0.71 to 1.77)	0.63
	Northern Ireland	37 (90.2)	4 (9.8)	0.46 (0.16 to 1.38)	0.17	0.33 (0.09 to 1.25)	0.10	0.32 (0.09 to 1.22)	0.09
	Scotland	166 (74.1)	58 (25.9)	1.39 (0.89 to 2.18)	0.15	1.32 (0.78 to 2.23)	0.30	1.33 (0.79 to 2.24)	0.29
	South East	273 (73.4)	99 (26.6)	1.46 (0.98 to 2.19)	0.06	1.61 (1.00 to 2.57)	0.05	1.61 (1.01 to 2.58)	0.04
	South West	181 (77.0)	54 (23.0)	1.19 (0.76 to 1.87)	0.45	1.30 (0.77 to 2.18)	0.32	1.30 (0.77 to 2.18)	0.32
	Wales	95 (72.0)	37 (28.0)	1.72 (1.03 to 2.88)	0.04	1.94 (1.09 to 3.43)	0.02	1.95 (1.11 to 3.45)	0.02
	West Midlands	268 (80.5)	65 (19.5)	0.99 (0.65 to 1.51)	0.96	1.24 (0.76 to 2.02)	0.39	1.24 (0.76 to 2.01)	0.39
	Yorkshire and the Humber	249 (80.1)	62 (19.9)	0.94 (0.61 to 1.46)	0.79	1.00 (0.61 to 1.64)	0.99	1.01 (0.61 to 1.66)	0.97

* Adjusting for survey wave, region, sex, age (raw and quadratic), dependent child in the household, being clinically vulnerable to COVID-19, having a household member with a chronic illness, employment status, highest earner is a manual worker, index of multiple deprivation, highest educational or professional qualification, ethnicity and living alone.

† Adjusting for survey wave, region, sex, age (raw and quadratic), dependent child in the household, being clinically vulnerable to COVID-19, having a household member with a chronic illness, employment status, socioeconomic grade, index of multiple deprivation, highest educational or professional qualification, ethnicity and living alone.

Table 4. Requesting a test after developing symptoms of COVID-19 and associations with survey wave and region. Bolding indicates findings significant at a *p*<0.001.

Participant characteristics	Level	Did not request a test n=2,402	Requested a test n=518	Odds ratio (95% CI) for requesting a test	p-value	Adjusted odds ratio (95% CI) for requesting a test *	p-value	Adjusted odds ratio (95% CI) for requesting a test †	p-value
Survey wave	26 to 27 May 2020 (wave 18)	155 (91.7)	14 (8.3)	Reference	-	Reference	-	Reference	-
	1 to 3 June 2020 (wave 19)	125 (86.8)	19 (13.2)	1.69 (0.82 to 3.49)	0.15	1.47 (0.69 to 3.12)	0.32	1.47 (0.69 to 3.13)	0.32
	8 to 10 June 2020 (wave 20)	117 (87.3)	17 (12.7)	1.61 (0.77 to 3.38)	0.21	1.28 (0.58 to 2.82)	0.55	1.28 (0.58 to 2.83)	0.54
	15 to 17 June 2020 (wave 21)	111 (87.4)	16 (12.6)	1.62 (0.77 to 3.42)	0.20	1.75 (0.81 to 3.75)	0.15	1.75 (0.81 to 3.76)	0.15
	22 to 24 June 2020 (wave 22)	116 (87.2)	17 (12.8)	1.61 (0.76 to 3.40)	0.21	1.66 (0.78 to 3.55)	0.19	1.66 (0.78 to 3.55)	0.19
	29 June to 1 July 2020 (wave 23)	107 (91.5)	10 (8.5)	1.06 (0.46 to 2.43)	0.90	0.85 (0.35 to 2.08)	0.72	0.85 (0.35 to 2.09)	0.73
	6 to 8 July 2020 (wave 24)	105 (89.7)	12 (10.3)	1.28 (0.57 to 2.86)	0.55	1.32 (0.57 to 3.03)	0.51	1.33 (0.58 to 3.05)	0.51
	20 to 22 July 2020 (wave 25)	105 (80.8)	25 (19.2)	2.63 (1.31 to 5.29)	0.007	2.42 (1.19 to 4.94)	0.02	2.42 (1.19 to 4.94)	0.01
	3 to 5 August 2020 (wave 26)	97 (90.7)	10 (9.3)	1.15 (0.49 to 2.68)	0.75	1.00 (0.40 to 2.50)	0.99	1.01 (0.40 to 2.51)	0.99
	1 to 2 September 2020 (wave 27)	117 (87.3)	17 (12.7)	1.59 (0.76 to 3.36)	0.22	1.34 (0.60 to 2.99)	0.48	1.34 (0.60 to 3.01)	0.47
	14 to 16 September 2020 (wave 28)	92 (77.3)	27 (22.7)	3.24 (1.62 to 6.48)	0.001	3.27 (1.63 to 6.57)	0.001	3.28 (1.63 to 6.61)	0.001
	28 to 30 September 2020 (wave 29)	92 (80.0)	23 (20.0)	2.75 (1.35 to 5.60)	0.005	2.65 (1.28 to 5.50)	0.009	2.67 (1.28 to 5.55)	0.009
	12 to 14 October 2020 (wave 30)	80 (74.1)	28 (25.9)	**3.90 (1.94 to 7.80)**	**<0.001**	3.35 (1.62 to 6.90)	0.001	3.36 (1.63 to 6.93)	0.001
	9 to 11 November 2020 (wave 32)	103 (81.7)	23 (18.3)	2.49 (1.23 to 5.04)	0.01	2.40 (1.17 to 4.92)	0.02	2.40 (1.17 to 4.93)	0.02
	16 to 18 November 2020 (wave 33)	103 (80.5)	25 (19.5)	2.70 (1.34 to 5.42)	0.005	2.74 (1.34 to 5.64)	0.006	2.76 (1.34 to 5.68)	0.006
	23 to 25 November 2020 (wave 34)	89 (79.5)	23 (20.5)	2.85 (1.40 to 5.80)	0.004	2.90 (1.39 to 6.02)	0.004	2.91 (1.40 to 6.05)	0.004
	30 November to 2 December 2020 (wave 35)	98 (82.4)	21 (17.6)	2.34 (1.14 to 4.82)	0.02	2.39 (1.14 to 5.01)	0.02	2.40 (1.14 to 5.03)	0.02
	7 to 9 December 2020 (wave 36)	88 (78.6)	24 (21.4)	3.01 (1.49 to 6.11)	0.002	3.04 (1.49 to 6.18)	0.002	3.05 (1.50 to 6.21)	0.002
	14 to 16 December 2020 (wave 37)	80 (72.7)	30 (27.3)	**4.12 (2.06 to 8.25)**	**<0.001**	**3.69 (1.80 to 7.53)**	**<0.001**	**3.69 (1.81 to 7.56)**	**<0.001**
	21 to 23 December 2020 (wave 38)	90 (78.3)	25 (21.7)	3.06 (1.52 to 6.17)	0.002	3.01 (1.45 to 6.27)	0.003	3.02 (1.45 to 6.31)	0.003
	28 to 30 December 2020 (wave 39)	78 (78.0)	22 (22.0)	3.14 (1.53 to 6.44)	0.002	2.83 (1.32 to 6.07)	0.008	2.83 (1.32 to 6.09)	0.008
	4 to 6 January 2021 (wave 40)	78 (66.1)	40 (33.9)	**5.67 (2.92 to 11.01)**	**<0.001**	**5.15 (2.57 to 10.35)**	**<0.001**	**5.16 (2.57 to 10.37)**	**<0.001**
	11 to 13 January 2021 (wave 41)	85 (78.0)	24 (22.0)	3.14 (1.55 to 6.36)	0.001	3.01 (1.47 to 6.17)	0.003	3.01 (1.47 to 6.18)	0.003
	25 to 27 January 2021 (wave 42)	91 (77.8)	26 (22.2)	3.18 (1.58 to 6.41)	0.001	2.96 (1.43 to 6.12)	0.003	2.97 (1.43 to 6.15)	0.003
Region	East Midlands	164 (79.6)	42 (20.4)	Reference	-	Reference	-	Reference	-
	East of England	191 (80.9)	45 (19.1)	0.94 (0.58 to 1.51)	0.79	0.82 (0.50 to 1.35)	0.44	0.82 (0.50 to 1.36)	0.45
	London	500 (83.6)	98 (16.4)	0.76 (0.51 to 1.15)	0.19	0.83 (0.53 to 1.28)	0.39	0.83 (0.53 to 1.28)	0.40

	North East	103 (79.8)	26 (20.2)	0.97 (0.56 to 1.69)	0.92	0.91 (0.51 to 1.62)	0.75	0.92 (0.52 to 1.62)	0.76
	North West	264 (80.0)	66 (20.0)	0.98 (0.63 to 1.51)	0.92	1.03 (0.64 to 1.65)	0.89	1.04 (0.65 to 1.66)	0.88
	Northern Ireland	33 (91.7)	3 (8.3)	0.36 (0.10 to 1.24)	0.11	0.41 (0.12 to 1.45)	0.17	0.41 (0.12 to 1.44)	0.16
	Scotland	171 (85.5)	29 (14.5)	0.66 (0.39 to 1.10)	0.11	0.69 (0.39 to 1.21)	0.20	0.69 (0.40 to 1.22)	0.20
	South East	259 (79.4)	67 (20.6)	1.00 (0.65 to 1.55)	0.98	0.94 (0.58 to 1.51)	0.79	0.94 (0.58 to 1.52)	0.80
	South West	173 (84.0)	33 (16.0)	0.75 (0.45 to 1.24)	0.26	0.75 (0.44 to 1.28)	0.29	0.75 (0.44 to 1.28)	0.29
	Wales	89 (79.5)	23 (20.5)	1.00 (0.57 to 1.77)	0.99	0.95 (0.52 to 1.74)	0.86	0.95 (0.52 to 1.75)	0.87
	West Midlands	231 (83.7)	45 (16.3)	0.75 (0.47 to 1.20)	0.23	0.80 (0.49 to 1.31)	0.37	0.80 (0.49 to 1.31)	0.37
	Yorkshire and the Humber	224 (84.5)	41 (15.5)	0.72 (0.44 to 1.16)	0.17	0.71 (0.42 to 1.19)	0.19	0.71 (0.42 to 1.19)	0.19

* Adjusting for survey wave, region, sex, age (raw and quadratic term), dependent child in the household, being clinically vulnerable to COVID-19, having a household member with a chronic illness, employment status, highest earner is a manual worker, index of multiple deprivation, highest educational or professional qualification, ethnicity, and living alone.

† Adjusting for survey wave, region, sex, age (raw and quadratic term), dependent child in the household, being clinically vulnerable to COVID-19, having a household member with a chronic illness, employment status, socioeconomic grade, index of multiple deprivation, highest educational or professional qualification, ethnicity, and living alone.

Table 5. Intention to share details of your close contacts with the NHS contact tracing service and associations with survey wave and region. Bolding indicates findings significant at a $p<0.001$.

Participant characteristics	Level	Probably or definitely would not share details of close contacts or not sure n=9,138	Probably or definitely would share details of close contacts n=34,299	Odds ratio (95% CI) for sharing details of close contacts	p-value	Adjusted odds ratio (95% CI) for sharing details of close contacts *	p-value	Adjusted odds ratio (95% CI) for sharing details of close contacts †	p-value
Survey wave‡	1 to 3 June 2020 (wave 19)	453 (24.7)	1382 (75.3)	Reference	-	Reference	-	Reference	-
	8 to 10 June 2020 (wave 20)	441 (23.9)	1402 (76.1)	1.04 (0.90 to 1.20)	0.57	1.02 (0.88 to 1.19)	0.78	1.02 (0.87 to 1.19)	0.82
	15 to 17 June 2020 (wave 21)	471 (25.3)	1390 (74.7)	0.98 (0.86 to 1.13)	0.80	0.96 (0.83 to 1.11)	0.58	0.96 (0.83 to 1.11)	0.57
	22 to 24 June 2020 (wave 22)	471 (25.4)	1382 (74.6)	0.94 (0.81 to 1.09)	0.42	0.88 (0.75 to 1.03)	0.10	0.88 (0.75 to 1.02)	0.10
	29 June to 1 July 2020 (wave 23)	433 (23.2)	1431 (76.8)	1.09 (0.95 to 1.26)	0.21	1.06 (0.91 to 1.24)	0.42	1.06 (0.91 to 1.23)	0.44
	6 to 8 July 2020 (wave 24)	451 (24.0)	1426 (76.0)	1.04 (0.90 to 1.19)	0.62	0.99 (0.85 to 1.15)	0.89	0.99 (0.85 to 1.14)	0.86
	20 to 22 July 2020 (wave 25)	449 (24.0)	1423 (76.0)	1.04 (0.90 to 1.19)	0.59	1.04 (0.90 to 1.21)	0.57	1.04 (0.90 to 1.21)	0.59
	3 to 5 August 2020 (wave 26)	391 (20.8)	1493 (79.2)	1.22 (1.06 to 1.41)	0.007	1.18 (1.01 to 1.38)	0.03	1.18 (1.01 to 1.38)	0.04
	1 to 2 September 2020 (wave 27)	385 (20.5)	1493 (79.5)	1.26 (1.09 to 1.47)	0.002	1.29 (1.10 to 1.52)	0.002	1.30 (1.10 to 1.52)	0.002
	14 to 16 September 2020 (wave 28)	361 (19.4)	1500 (80.6)	**1.33 (1.15 to 1.55)**	**<0.001**	1.30 (1.10 to 1.52)	0.001	1.29 (1.10 to 1.52)	0.002
	28 to 30 September 2020 (wave 29)	387 (20.2)	1531 (79.8)	1.26 (1.09 to 1.46)	0.002	1.18 (1.02 to 1.38)	0.03	1.19 (1.02 to 1.38)	0.03
	12 to 14 October 2020 (wave 30)	363 (18.6)	1592 (81.4)	**1.41 (1.22 to 1.64)**	**<0.001**	1.29 (1.10 to 1.50)	0.002	1.29 (1.10 to 1.51)	0.001
	9 to 11 November 2020 (wave 32)	373 (19.7)	1522 (80.3)	**1.31 (1.13 to 1.51)**	**<0.001**	1.19 (1.02 to 1.38)	0.03	1.19 (1.02 to 1.38)	0.03
	16 to 18 November 2020 (wave 33)	408 (21.3)	1510 (78.7)	1.19 (1.03 to 1.38)	0.02	1.15 (0.98 to 1.34)	0.08	1.15 (0.99 to 1.34)	0.07
	23 to 25 November 2020 (wave 34)	334 (17.6)	1566 (82.4)	**1.50 (1.29 to 1.74)**	**<0.001**	**1.37 (1.17 to 1.61)**	**<0.001**	**1.37 (1.17 to 1.61)**	**<0.001**
	30 November to 2 December 2020 (wave 35)	379 (19.7)	1544 (80.3)	**1.32 (1.14 to 1.53)**	**<0.001**	1.28 (1.09 to 1.50)	0.002	1.28 (1.09 to 1.50)	0.002
	7 to 9 December 2020 (wave 36)	397 (20.0)	1584 (80.0)	1.27 (1.10 to 1.47)	0.001	1.18 (1.01 to 1.37)	0.04	1.18 (1.01 to 1.37)	0.04
	14 to 16 December 2020 (wave 37)	405 (21.2)	1501 (78.8)	1.19 (1.03 to 1.38)	0.02	1.14 (0.98 to 1.33)	0.09	1.14 (0.98 to 1.33)	0.09
	21 to 23 December 2020 (wave 38)	363 (19.1)	1533 (80.9)	**1.34 (1.16 to 1.56)**	**<0.001**	1.26 (1.08 to 1.48)	0.003	1.26 (1.08 to 1.48)	0.003
	28 to 30 December 2020 (wave 39)	365 (19.7)	1486 (80.3)	1.30 (1.12 to 1.51)	0.001	1.25 (1.07 to 1.46)	0.006	1.25 (1.06 to 1.46)	0.006
	4 to 6 January 2021 (wave 40)	350 (18.5)	1544 (81.5)	**1.41 (1.22 to 1.64)**	**<0.001**	1.27 (1.09 to 1.49)	0.003	1.27 (1.08 to 1.48)	0.003
	11 to 13 January 2021 (wave 41)	366 (19.4)	1522 (80.6)	**1.33 (1.15 to 1.55)**	**<0.001**	1.22 (1.04 to 1.42)	0.01	1.21 (1.04 to 1.42)	0.02
	25 to 27 January 2021 (wave 42)	342 (18.2)	1542 (81.8)	**1.44 (1.24 to 1.67)**	**<0.001**	1.29 (1.10 to 1.51)	0.002	1.29 (1.10 to 1.52)	0.002
Region	East Midlands	717 (20.7)	2744 (79.3)	Reference	-	Reference	-	Reference	-
	East of England	865 (20.4)	3371 (79.6)	0.97 (0.85 to 1.09)	0.57	0.97 (0.85 to 1.11)	0.70	0.98 (0.86 to 1.12)	0.75

	London	1271 (25.0)	3805 (75.0)	**0.73 (0.65 to 0.82)**	**<0.001**	0.93 (0.82 to 1.06)	0.29	0.92 (0.81 to 1.05)	0.22
	North East	376 (19.1)	1589 (80.9)	1.04 (0.89 to 1.22)	0.61	1.14 (0.96 to 1.34)	0.13	1.14 (0.97 to 1.34)	0.12
	North West	1081 (22.1)	3810 (77.9)	0.87 (0.78 to 0.98)	0.03	0.96 (0.84 to 1.08)	0.48	0.95 (0.84 to 1.08)	0.44
	Northern Ireland	181 (22.5)	622 (77.5)	0.88 (0.72 to 1.09)	0.25	0.98 (0.79 to 1.22)	0.86	0.98 (0.78 to 1.22)	0.84
	Scotland	629 (18.5)	2778 (81.5)	1.10 (0.97 to 1.26)	0.15	1.15 (1.00 to 1.32)	0.05	1.15 (.001 to 1.32)	0.05
	South East	1167 (20.0)	4659 (80.0)	0.99 (0.88 to 1.11)	0.82	0.99 (0.88 to 1.12)	0.90	0.99 (0.87 to 1.12)	0.87
	South West	720 (19.0)	3071 (81.0)	1.04 (0.91 to 1.18)	0.57	1.06 (0.92 to 1.21)	0.44	1.06 (0.92 to 1.21)	0.43
	Wales	390 (18.1)	1770 (81.9)	1.10 (0.95 to 1.28)	0.22	1.15 (0.97 to 1.35)	0.10	1.14 (0.97 to 1.34)	0.10
	West Midlands	898 (23.4)	2934 (76.6)	0.81 (0.72 to 0.92)	0.001	0.93 (0.81 to 1.06)	0.25	0.93 (0.81 to 1.06)	0.25
	Yorkshire and the Humber	843 (21.1)	3146 (78.9)	0.93 (0.82 to 1.05)	0.25	1.03 (0.90 to 1.17)	0.70	1.02 (0.89 to 1.17)	0.75

* Adjusting for survey wave, region, sex, age (raw and quadratic term), dependent child in the household, being clinically vulnerable to COVID-19, having a household member with a chronic illness, employment status, highest earner is a manual worker, index of multiple deprivation, highest educational or professional qualification, ethnicity, and living alone.

† Adjusting for survey wave, region, sex, age (raw and quadratic term), dependent child in the household, being clinically vulnerable to COVID-19, having a household member with a chronic illness, employment status, socio-economic grade, index of multiple deprivation, highest educational or professional qualification, ethnicity, and living alone.

‡ For most GEE analyses, we used an exchangeable correlation structure. This failed to converge for the univariable analysis for survey wave, so we used an unstructured correlation structure.

Supplementary materials. Associations between personal and clinical characteristics and fully self-isolating after developing symptoms of COVID-19. Bolding indicates findings significant at a *p*<0.001.

Participant characteristics	Level	Did not fully self-isolate n=2,717	Fully self-isolated n=680	Odds ratio (95% CI) for fully self-isolating	p-value	Adjusted odds ratio (95% CI) for fully self-isolating *	p-value	Adjusted odds ratio (95% CI) for fully self-isolating †	p-value
Survey wave	Overall	-	-	**χ^2(29)=64.0**	**<0.001**	χ^2(29)=57.3	0.001	χ^2(29)=56.3	0.002
Region	Overall	-	-	**χ^2(11)=45.5**	**<0.001**	χ^2(11)=20.3	0.04	χ^2(11)=20.6	0.04
Gender	Male	1562 (83.5)	308 (16.5)	Reference	-	Reference	-	Reference	-
	Female	1148 (75.8)	366 (24.2)	**1.66 (1.39 to 1.97)**	**<0.001**	**1.87 (1.53 to 2.29)**	**<0.001**	**1.89 (1.55 to 2.31)**	**<0.001**
Age (per decade)	Raw age	N=2,717, M=34.8, SD=13.6	N=680, M=42.4, SD=17.0	**1.37 (1.30 to 1.46)**	**<0.001**	**1.28 (1.19 to 1.38)**	**<0.001**	**1.29 (1.20 to 1.39)**	**<0.001**
Age: quadratic (age-mean)^2	-	-	-	-	-	0.9998 (0.9995 to 1.0002)	0.33	0.9998 (0.9994 to 1.0001)	0.23
Dependent child in household	None	1139 (74.2)	397 (25.8)	Reference	-	Reference	-	Reference	-
	Child present	1578 (84.8)	283 (15.2)	**0.54 (0.46 to 0.65)**	**<0.001**	0.69 (0.55 to 0.87)	0.002	0.69 (0.54 to 0.87)	0.001
Clinically vulnerable to COVID-19	None	1746 (79.6)	448 (20.4)	Reference	-	Reference	-	Reference	-
	Present	765 (79.6)	196 (20.4)	1.00 (0.83 to 1.20)	0.97	0.81 (0.66 to 1.00)	0.05	0.82 (0.67 to 1.01)	0.06
Household member has chronic illness	None	2077 (78.9)	554 (21.1)	Reference	-	Reference	-	Reference	-
	Present	583 (84.1)	110 (15.9)	0.68 (0.54 to 0.85)	0.001	0.69 (0.54 to 0.90)	0.005	0.69 (0.53 to 0.88)	0.004
Employment status	Not working	810 (73.9)	286 (26.1)	Reference	-	Reference	-	Reference	-
	Working	1873 (83.2)	379 (16.8)	**0.60 (0.51 to 0.72)**	**<0.001**	0.76 (0.61 to 0.94)	0.01	0.70 (0.56 to 0.86)	0.001
Highest earner works in a manual occupation	No	1353 (75.9)	429 (24.1)	Reference	-	Reference	-	-	-
	Yes	1333 (85.0)	235 (15.0)	**0.58 (0.49 to 0.69)**	**<0.001**	**0.69 (0.56 to 0.84)**	**<0.001**	-	-
Socioeconomic grade	ABC1 (high)	1134 (78.5)	310 (21.5)	Reference	-	-	-	Reference	-
	C2DE	1552 (81.4)	354 (18.6)	0.83 (0.70 to 0.98)	0.03	-	-	0.75 (0.62 to 0.91)	0.003
Index of multiple deprivation	1st quartile (least deprived)	369 (78.5)	101 (21.5)	1.24 (0.94 to 1.62)	0.12	0.96 (0.70 to 1.33)	0.82	0.94 (0.68 to 1.31)	0.72
	2nd quartile	479 (75.8)	153 (24.2)	1.40 (1.10 to 1.77)	0.006	1.12 (0.85 to 1.47)	0.43	1.10 (0.83 to 1.45)	0.50
	3rd quartile	791 (81.2)	183 (18.8)	1.06 (0.85 to 1.32)	0.59	0.94 (0.73 to 1.22)	0.66	0.94 (0.73 to 1.21)	0.61
	4th quartile (most deprived)	1078 (81.6)	243 (18.4)	Reference	-	Reference	-	Reference	-
	Overall	-	-	χ^2(3)=8.7	0.03	χ^2(3)=1.5	0.69	χ^2(3)=1.4	0.71
Highest educational or professional qualification	GCSE/vocational/A-level/No formal qualifications	1364 (75.5)	443 (24.5)	Reference	-	Reference	-	Reference	-

	Degree or higher (Bachelors, Masters, PhD)	1353 (85.1)	237 (14.9)	**0.56 (0.47 to 0.67)**	**<0.001**	**0.61 (0.50 to 0.74)**	**<0.001**	**0.60 (0.49 to 0.73)**	**<0.001**
Ethnicity	White British	1799 (77.8)	512 (22.2)	Reference	-	Reference	-	Reference	-
	White other	461 (88.1)	62 (11.9)	**0.46 (0.35 to 0.60)**	**<0.001**	0.60 (0.43 to 0.84)	0.003	0.58 (0.41 to 0.81)	0.001
	Black and minority ethnicity	447 (81.9)	99 (18.1)	0.78 (0.61 to 0.99)	0.04	1.11 (0.83 to 1.48)	0.47	1.12 (0.84 to 1.49)	0.46
	Overall	-	-	**$\chi^2(2)=31.4$**	**<0.001**	$\chi^2(2)=10.7$	0.005	$\chi^2(2)=2.3$	0.002
Living alone	Not living alone	2287 (80.6)	551 (19.4)	Reference	-	Reference	-	Reference	-
	Living alone	430 (76.9)	129 (23.1)	1.19 (0.96 to 1.48)	0.12	0.85 (0.64 to 1.13)	0.27	0.87 (0.65 to 1.15)	0.32
Work in key sectors	No	403 (70.8)	166 (29.2)	Reference	-	Reference	-	Reference	-
	Yes	1718 (86.5)	268 (13.5)	**0.39 (0.31 to 0.49)**	**<0.001**	**0.51 (0.39 to 0.68)**	**<0.001**	**0.51 (0.39 to 0.67)**	**<0.001**
Self-employed‡	No	1683 (82.5)	358 (17.5)	Reference	-	Reference	-	Reference	-
	Yes	190 (90.0)	21 (10.0)	0.52 (0.33 to 0.81)	0.004	0.67 (0.40 to 1.14)	0.14	0.69 (0.41 to 1.17)	0.17
Marital status	Single/separated/divorced /widowed	1138 (79.2)	299 (20.8)	Reference	-	Reference	-	Reference	-
	Married/partnered	1462 (79.8)	369 (20.2)	0.98 (0.83 to 1.17)	0.85	0.95 (0.76 to 1.17)	0.62	0.93 (0.75 to 1.16)	0.53
Ever had COVID-19	Think have not had COVID-19	1420 (74.8)	478 (25.2)	Reference	-	Reference	-	Reference	-
	Think or had COVID-19 confirmed	1297 (86.5)	202 (13.5)	**0.51 (0.42 to 0.60)**	**<0.001**	**0.59 (0.48 to 0.73)**	**<0.001**	**0.59 (0.48 to 0.72)**	**<0.001**
Attribute current symptoms to COVID-19§	No	1397 (80.5)	339 (19.5)	Reference	-	Reference	-	Reference	-
	Yes	423 (79.5)	109 (20.5)	1.11 (0.88 to 1.41)	0.38	1.29 (0.97 to 1.71)	0.08	1.28 (0.96 to 1.71)	0.09
Hardship‖	Range 3 (least hardship) to 15 (most hardship)	N=2,392, M=10.4, SD=2.6	N=590, M=9.4, SD=2.9	**0.88 (0.85 to 0.91)**	**<0.001**	**0.91 (0.87 to 0.95)**	**<0.001**	**0.91 (0.87 to 0.95)**	**<0.001**

Abbreviations: N=number; M=mean; SD=standard deviation; CI=confidence intervals.

For continuous variables, odds ratios represent a one-unit increase in the explanatory variable, apart from for age, where odds ratios represent a ten-year increase in age.

* Adjusting for survey wave, region, sex, age (raw and quadratic), dependent child in the household, clinically vulnerable to covid-19, household member has chronic illness, employment status, highest earner works in a manual occupation, index of multiple deprivation, highest educational or professional qualification, ethnicity, and living alone.

† Adjusting for survey wave, region, sex, age (raw and quadratic term), dependent child in the household, clinically vulnerable to covid-19, household member has chronic illness, employment status, socio-economic grade, index of multiple deprivation, highest educational or professional qualification, ethnicity, and living alone.

‡ Not adjusting for employment status as by definition all people asked whether they were self-employed were working.

§ Item added to survey in wave 21 (15 June 2020)

‖ Items added to survey in wave 14 (27 April 2020)

Supplementary materials. Self-reported reasons for not adhering to test, trace and isolate behaviours.

Self-isolation in those not adherent to duration-adjusted self-isolation, excluding those who tested negative (n=698)

Self-reported reasons for having left home since having developed symptoms (participants could select multiple response options)	n	%
To go to the shops, for groceries/pharmacy	150	21.5
To go out to work	110	15.8
To go to the shops, for things other than groceries/pharmacy	109	15.6
My symptoms did not persist / were temporary	106	15.2
For a medical need (other than coronavirus)	105	15.0
To go for a walk or some other exercise	103	14.8
My symptoms were only mild	101	14.5
My symptoms got better	97	13.9
I don't think it is necessary for me to stay at home	92	13.2
I was too bored	85	12.2
To help or provide care for a vulnerable person	83	11.9
To meet up with friends and/or family	79	11.3
I was too depressed or anxious	78	11.2
I didn’t think it was that risky*	49	9.8
I was too lonely	68	9.7
My symptoms got worse	59	8.5
To get or return a test for coronavirus†	32	7.6

* Item added on 23 November 2020 (wave 34); baseline n=498.
† Item added on 23 November 2020 (wave 34); baseline n=421. Different baseline n due to exclusion of those who tested negative.

Requesting a test (n=2,230).

Self-reported reasons for not requesting a test to confirm whether you had coronavirus (participants could select multiple response options)	n	%
I didn't think my symptoms were due to coronavirus	467	20.9
My symptoms improved*	196	16.9
My symptoms were only mild*	189	16.3
My symptoms were only mild or improved §	144	13.5
I hadn't been in contact with anyone who had coronavirus recently	291	13.0
I thought I only needed to self-isolate (not leaving the home at all)	256	11.5
I didn't want to use a test that could have gone to someone else who needed it more	247	11.1
I didn't think I was eligible to get a test	245	11.0
I was worried about how colleagues/my employer would react if I tested positive †	188	10.0
I didn't know how to request a test	208	9.3
I was sure I had coronavirus, so I didn't see a reason to take a test	206	9.2
I have already had coronavirus, so didn't see a reason to take a test	198	8.9
I was worried what friends or family would think about me if I tested positive†	158	8.4
I thought it would take too long to get a test result §	86	8.0
I thought the test would be uncomfortable or painful	171	7.7
I didn’t think tests were available near me ‡	96	7.7
I didn't think the test would be accurate	169	7.6
I didn't want to know the results of a test	168	7.5
I didn’t want to have a coronavirus test result on my medical record §	77	7.2
I didn’t want to have to ask the people who I live or who I have met with to self-isolate §	74	6.9
I didn't know what the test involved	151	6.8
I didn’t want to have to self-isolate §	70	6.5
I didn’t want to increase the case numbers in my local area because this leads to tighter restrictions §	70	6.5
I didn’t want the police or other public bodies to be notified §	64	6.0
I was worried that I might get fined §	59	5.5

* Items last included on 14 October 2020 (wave 30); baseline n=1,160.

† Items added on 29 June 2020 (wave 23); baseline n=1,882.

‡ Item added on 28 September (wave 29); baseline n=1,242.

§ Items added on 26 October 2020 (wave 31); baseline n=1,070.

Intention to share details of close contacts (n=45,720)

Self-reported reasons that would stop you passing details of your recent close contacts to the NHS (participants could select multiple response options)	n	%
I don't know if the data will be secure and confidential	6,674	14.6
I don't think the contact tracing system is accurate and reliable	6,355	13.9
I don't know what will happen to the data	5,929	13.0
It would cause stress to my contacts	3,409	7.5
It would cause inconvenience to my contacts	2,766	6.0
It would cause loss of income to my contacts	2,431	5.3
I don't want to be responsible of other people being told to self-isolate	2,163	4.7
My contacts would be angry	2,083	4.6
I am worried about being found out by my contacts	1,571	3.4
None of the above, I would share details of recent close contacts with the NHS if asked to do so (single code)	29,102	63.6

Supplementary materials. Associations between personal and clinical characteristics and requesting a test after developing symptoms of COVID-19. Bolding indicates findings significant at a *p*<0.001.

Participant characteristics	Level	Did not request a test n=2,402	Requested a test n=518	Odds ratio (95% CI) for requesting a test	p-value	Adjusted odds ratio (95% CI) for requesting a test *	p-value	Adjusted odds ratio (95% CI) for requesting a test †	p-value
Survey wave	Overall	-	-	**χ^2(23)=75.9**	**<0.001**	**χ^2(23)=65.9**	**<0.001**	**χ^2(23)=66.0**	**<0.001**
Region	Overall	-	-	χ^2(11)=11.2	0.43	χ^2(11)=6.9	0.81	χ^2(11)=7.0	0.80
Gender	Male	1354 (85.1)	237 (14.9)	Reference	-	Reference	-	Reference	-
	Female	1037 (78.8)	279 (21.2)	**1.54 (1.27 to 1.87)**	**<0.001**	1.41 (1.14 to 1.74)	0.002	1.41 (1.14 to 1.74)	0.001
Age (per decade)	Raw age	N=2,402, M=36.9, SD=15.0	N=518, M=36.5, SD=13.7	0.99 (0.93 to 1.05)	0.67	0.93 (0.84 to 1.03)	0.17	0.93 (0.84 to 1.03)	0.18
Age: quadratic (age-mean)^2	-	-	-	-	-	0.9997 (0.9992 to 1.0001)	0.15	0.9997 (0.9992 to 1.0001)	0.14
Dependent child in household	None	1093 (82.9)	226 (17.1)	Reference	-	Reference	-	Reference	-
	Child present	1309 (81.8)	292 (18.2)	1.07 (0.88 to 1.29)	0.52	1.03 (0.80 to 1.32)	0.82	1.03 (0.80 to 1.31)	0.84
Clinically vulnerable to COVID-19	None	1558 (82.3)	336 (17.7)	Reference	-	Reference	-	Reference	-
	Present	668 (82.2)	145 (17.8)	1.01 (0.81 to 1.25)	0.96	1.06 (0.84 to 1.33)	0.64	1.06 (0.84 to 1.34)	0.62
Household member has chronic illness	None	1859 (82.1)	404 (17.9)	Reference	-	Reference	-	Reference	-
	Present	494 (82.6)	104 (17.4)	0.97 (0.76 to 1.23)	0.79	1.12 (0.86 to 1.46)	0.38	1.12 (0.86 to 1.46)	0.38
Employment status	Not working	828 (84.3)	154 (15.7)	Reference	-	Reference	-	Reference	-
	Working	1537 (81.2)	356 (18.8)	1.25 (1.01 to 1.53)	0.04	1.21 (0.94 to 1.57)	0.14	1.19 (0.92 to 1.54)	0.19
Highest earner works in a manual occupation	No	1272 (81.6)	286 (18.4)	Reference	-	Reference	-	-	-
	Yes	1093 (83.2)	221 (16.8)	0.90 (0.74 to 1.09)	0.27	0.93 (0.75 to 1.16)	0.51	-	-
Socio-economic grade	ABC1 (high)	1027 (81.5)	233 (18.5)	Reference	-	-	-	Reference	-
	C2DE	1338 (83.0)	274 (17.0)	0.90 (0.74 to 1.10)	0.30	-	-	0.92 (0.74 to 1.15)	0.47
Index of multiple deprivation	1st quartile (least deprived)	334 (81.7)	75 (18.3)	1.14 (0.84 to 1.53)	0.39	1.16 (0.82 to 1.63)	0.40	1.15 (0.82 to 1.62)	0.42
	2nd quartile	430 (80.1)	107 (19.9)	1.26 (0.96 to 1.64)	0.09	1.22 (0.91 to 1.65)	0.18	1.22 (0.90 to 1.64)	0.20
	3rd quartile	703 (82.4)	150 (17.6)	1.08 (0.85 to 1.37)	0.54	1.08 (0.83 to 1.41)	0.56	1.08 (0.83 to 1.41)	0.57
	4th quartile (most deprived)	935 (83.4)	186 (16.6)	Reference	-	Reference	-	Reference	-
	Overall	-	-	χ^2(3)=3.0	0.39	χ^2(3)=1.9	0.59	χ^2(3)=1.8	0.62
Highest educational or	GCSE/vocational/A-level/No formal qualifications	1303 (81.7)	291 (18.3)	Reference	-	Reference	-	Reference	-

professional qualification	Degree or higher (Bachelors, Masters, PhD)	1099 (82.9)	227 (17.1)	0.92 (0.76 to 1.11)	0.39	1.02 (0.82 to 1.27)	0.84	1.02 (0.82 to 1.27)	0.87
Ethnicity	White British	1638 (81.7)	368 (18.3)	Reference	-	Reference	-	Reference	-
	White other	368 (87.0)	55 (13.0)	0.66 (0.48 to 0.90)	0.009	0.73 (0.51 to 1.03)	0.07	0.72 (0.51 to 1.02)	0.06
	Black and minority ethnicity	381 (80.2)	94 (19.8)	1.09 (0.85 to 1.41)	0.49	1.01 (0.75 to 1.36)	0.94	1.01 (0.75 to 1.36)	0.94
	Overall	-	-	$\chi^2(2)=8.2$	0.02	$\chi^2(2)=3.5$	0.17	$\chi^2(2)=3.6$	0.16
Living alone	Not living alone	2015 (82.1)	439 (17.9)	Reference	-	Reference	-	Reference	-
	Living alone	387 (83.0)	79 (17.0)	0.94 (0.72 to 1.23)	0.66	1.10 (0.78 to 1.54)	0.59	1.10 (0.79 to 1.54)	0.57
Work in key sectors	No	416 (82.4)	89 (17.6)	Reference	-	Reference	-	Reference	-
	Yes	1348 (81.3)	310 (18.7)	1.08 (0.83 to 1.40)	0.56	1.35 (1.01 to 1.81)	0.04	1.34 (1.00 to 1.80)	0.05
Self-employed	No	1400 (81.3)	321 (18.7)	Reference	-	Reference	-	Reference	-
	Yes	137 (79.7)	35 (20.3)	1.10 (0.74 to 1.64)	0.63	1.23 (0.78 to 1.94)	0.37	1.24 (0.78 to 1.95)	0.36
Marital status	Single/separated/divorced/widowed	1023 (84.1)	194 (15.9)	Reference	-	Reference	-	Reference	-
	Married/partnered	1283 (80.7)	306 (19.3)	1.26 (1.03 to 1.53)	0.02	1.17 (0.92 to 1.50)	0.20	1.17 (0.91 to 1.50)	0.22
Ever had COVID-19	Think have not had COVID-19	1403 (82.9)	290 (17.1)	Reference	-	Reference	-	Reference	-
	Think or had COVID-19 confirmed	999 (81.4)	228 (18.6)	1.10 (0.90 to 1.33)	0.35	1.08 (0.87 to 1.35)	0.47	1.08 (0.87 to 1.34)	0.49
Attribute current symptoms to COVID-19§	No	1581 (83.4)	315 (16.6)	Reference	-	Reference	-	Reference	-
	Yes	424 (73.5)	153 (26.5)	**1.80 (1.44 to 2.24)**	**<0.001**	**1.81 (1.42 to 2.31)**	**<0.001**	**1.80 (1.41 to 2.30)**	**<0.001**
Hardship	Range 3 (least hardship) to 15 (most hardship)	N=2,281, M=10.3, SD=2.7	N=500, M=9.9, SD=2.8	0.94 (0.91 to 0.98)	0.002	**0.93 (0.89 to 0.97)**	**<0.001**	**0.93 (0.89 to 0.97)**	**<0.001**

Abbreviations: N=number; M=mean; SD=standard deviation; CI=confidence intervals.

For continuous variables, odds ratios represent a one-unit increase in the explanatory variable, apart from for age, where odds ratios represent a ten-year increase in age.

* Adjusting for survey wave, region, sex, age (raw and quadratic term), dependent child in the household, clinically vulnerable to covid-19, household member has chronic illness, employment status, highest earner works in a manual occupation, index of multiple deprivation, highest educational or professional qualification, ethnicity, and living alone.

† Adjusting for survey wave, region, sex, age (raw and quadratic term), dependent child in the household, clinically vulnerable to covid-19, household member has chronic illness, employment status, socio-economic grade, index of multiple deprivation, highest educational or professional qualification, ethnicity, and living alone.

§This item was introduced to the questionnaire in wave 21 (15 June 2020).
