# Supplementary material for: Adherence to the test, trace, and isolate system in the UK: results from 37 nationally representative surveys
Source: BMJ. 2022 Aug 16;378:o2008. doi: 10.1136/bmj.o2008 (PMC9379914; doi:10.1136/bmj.o2008)
Supplement: Supplementary file 1 — Supplementary information: pdf of paper showing revisions in tracked changes [file correction2064.ww.pdf]

Adherence to the test, trace, and isolate system in the UK: results from 37 nationally representative surveys

Louise E Smith(https://orcid.org/0000-0002-1277-2564),[1,2] postdoctoral researcher, Henry W W Potts(https://orcid.org/0000-0002-6200-8804),[3] professor of health informatics, Richard Amlôt,[2,4] professor of practice in the psychology of health protection , Nicola T Fear(https://orcid.org/0000-0002-5792-2925),[1,5] professor of epidemiology, Susan Michie(https://orcid.org/0000-0003-0063-6378),[6] professor of health psychology, G James Rubin,[1,2] professor of psychology and emerging health risks

[1] Department of Psychological Medicine, Institute of Psychiatry, Psychology and Neuroscience, Weston Education Centre, King's College London, London SE5 9RJ, UK

[2] NIHR Health Protection Research Unit in Emergency Preparedness and Response, King's College London, London, UK

[3] Institute of Health Informatics, University College London, London, UK

[4] Public Health England, Behavioural Science Team, Emergency Response Department Science and Technology, Porton Down, Salisbury, UK

[5] King's Centre for Military Health Research and Academic Department of Military Mental Health, King's College London, London, UK

[6] Centre for Behaviour Change, University College London, London, UK

Correspondence to: L E Smith louise.e.smith@kcl.ac.uk (or @louisesmith142 on Twitter)

Accepted: 2 March 2021

Abstract

Objective To investigate rates of adherence to the UK's test, trace, and isolate system over the initial 11 months of the covid-19 pandemic.
Design Series of cross sectional online surveys.
Setting 37 nationally representative surveys in the UK, 2 March 2020 to 27 January 2021.
Participants 74 699~~7~~ responses from 45~~3~~ 957~~880~~ people living in the UK, aged 16 years or older (37 survey waves, about 2000 participants in each wave).
Main outcome measures Identification of the main symptoms of covid-19 (cough, high temperature or fever, and loss of sense of smell or taste), self-reported adherence to self-isolation if symptoms were present and intention to self-isolate if symptoms were to develop, requesting a test for covid-19 if symptoms were present and intention to request a test if symptoms were to develop, and intention to share details of close contacts.

Results Only 51.5% of participants (95% confidence interval 51.0% to 51.9%, n=26 030/50 570) identified the main symptoms of covid-19; the corresponding values in the most recent wave of data collection (25-27 January 2021) were 50.8% (48.6% to 53.0%, n=1019/2007). Across all waves, duration adjusted adherence to full self-isolation was 42.5% (95% confidence interval 39.7% to 45.2%, n=515/1213); in the most recent wave of data collection (25-27 January 2021), it was 51.8% (40.8% to 62.8%, n=43/83). Across all waves, requesting a test for covid-19 was 18.0% (95% confidence interval 16.6% to 19.3%, n=552/3068), increasing to 22.2% (14.6% to 29.9%, n=26/117) from 25 to 27 January. Across all waves, intention to share details of close contacts was 79.1% (95% confidence interval 78.8% to 79.5%, n=36 145/45 680), increasing to 81.9% (80.1% to 83.6%, n=1547/1890) from 25 to 27 January. Non-adherence was associated with being male, younger age, having a dependent child in the household, lower socioeconomic ~~grade~~status, greater financial hardship during the pandemic, and working in a key sector.
Conclusions Levels of adherence to test, trace, and isolate are low, although some improvement has occurred over time. Practical support and financial reimbursement are likely to improve adherence. Targeting messaging and policies to men, younger age groups, and key workers might also be necessary.

Introduction

Governments around the world have relied on test, trace, and isolate strategies to separate infected people from non-infected people and prevent the spread of covid-19.[1] Test, trace, and isolate is a less disruptive measure than alternative population-wide restrictions in activity. Within the UK, guidance for people who might have covid-19 has evolved over time but has focused on the need for people with a persistent new onset cough, fever, or loss of sense of taste or smell to remain at home for at least seven days from the onset of symptoms (self-isolate), request a test to confirm whether they have covid-19, and, if the test result is positive, provide details of close contacts to a dedicated service. These principles are the same in each of the four UK nations (England, Wales, Scotland, and Northern Ireland), although each nation has its own test, trace, and isolate system.[2-5]

The ability of the test, trace, and isolate system to keep rates of infection under control relies on how well people adhere to guidance on testing, provide details of contacts, and self-isolate, which in turn depends on their knowledge, motivation, and opportunity to do so.[6 7] From when an infected person develops symptoms to when their contacts are allowed to come out of quarantine, adherence might break down at multiple stages.[8] In the UK, knowledge of the symptoms of covid-19 has been shown to be poor.[9 10] Financial constraints and cramped accommodation have been identified as factors that affect whether people will remain at home during the pandemic.[10 11 12] Some evidence

suggests that men and younger age groups are less adherent to covid-19 restrictions,[13] as are those who think they have been infected with SARS-CoV-2.[14]

Identifying key factors that increase or decrease adherence can be used to inform policies to improve the functioning of the test, trace, and isolate system. Since the start of the covid-19 pandemic, we have worked with England's Department of Health and Social Care to develop and analyse a series of regular cross sectional surveys tracking relevant behaviours and their potential predictors in the UK public. We report data from 37 of these surveys that tracked adherence to the key components of the system over time and investigate personal and clinical characteristics that might be related to adherence to full self-isolation when someone has symptoms, requesting a test if symptoms are present, and intending to share details of close contacts if symptomatic. We also investigated variables associated with correctly identifying the main symptoms of covid-19.

Methods

Design

BMG Research, a Market Research Society company partner, conducted a series of cross sectional online surveys on behalf of the Department of Health and Social Care starting on 28 January 2020, which we analysed as part of the CORSAIR (the COVID-19 Rapid Survey of Adherence to Interventions and Responses) study. Surveys were conducted weekly until 1 July (wave 23), after which survey waves were fortnightly; the weekly survey was resumed between 9 November 2020 and 13 January 2021. No data were collected in mid-August 2020. We used data from surveys conducted between 2 March 2020 (wave 6) and 27 January 2021 (wave 42). Data were collected over a three day period (Monday to Wednesday) for each survey wave, except for wave 6 (collected Monday to Thursday) and waves 12, 18, and 27 (collected Tuesday to Wednesday). As prompt turnaround of data collection is essential during a rapidly evolving crisis,[15] the surveys used standard opinion polling methods using non-probability sampling, an approach common within market research, political polling, and social science.[16] Quota samples aim to minimise response bias by filling predetermined targets so that the social and personal characteristics of the participants match those of the

national population. As such, participants who belong to a quota that has already been met are prevented from completing the survey. Therefore, response rates are not useful indicators of response bias in quota samples and are not usually reported.

Participants

This study reports on 74 699~~7~~ responses from 45~~3~~ 957~~880~~ participants across the four UK nations. Participants (about 2000 in each wave) were recruited from two specialist research panel providers, Respondi (n=50 000) and Savanta (n=31 500).[17 18] Participants in the first seven waves were recruited from Respondi only; subsequent waves included roughly equal numbers from each panel. Participants were eligible for the study if they were aged 16 years or older and lived in the UK. Respondents who completed the survey were unable to participate in the following three waves. Owing to an error, a few people completed waves more often than others; 28~~55~~ people (0.1% of our sample) completed 10 waves or more. Quotas were applied based on age and sex (combined) and government office region and reflected targets based on data from the Office for National Statistics.[19] Therefore, the sociodemographic characteristics of participants in each survey wave were broadly similar to those in the UK general population. Participants were reimbursed in points, which could be redeemed in cash, gift vouchers, or charitable donations (up to £0.70 ($0.98; €0.81) for each survey).

Outcome measures

Identification of covid-19 symptoms—One question asked participants to identify the most common symptoms of covid-19, with multiple response options allowed (up to four initially, up to five from 25 May 2020, wave 18). We coded participants as having identified symptoms of covid-19 if they selected cough, high temperature or fever, and, from 18 May 2020 (wave 17), either loss of sense of smell or loss of sense of taste. In government guidance these symptoms are actively promoted to members of the UK public as the “main” symptoms of covid-19.[20]

Fully self-isolating—We measured self-reported self-isolation in participants who indicated that they had experienced symptoms of covid-19 (high temperature or fever, cough, or loss of sense of smell or taste) in the past seven days. Participants were asked for what reason, if any, they had left home since the development of symptoms. We categorised people as non-adherent if they reported leaving home for any reason since symptoms developed. From 26 October 2020 (wave 31) we also asked participants how soon (in days) they had first left home after symptoms

developed. We used this to create a second outcome variable (duration adjusted adherence) and categorised people as non-adherent if they reported leaving home for any reason in the first 10 days after symptoms developed. This adjustment allowed for the fact that, during that period, self-isolation was only required for 10 days after symptom onset. We measured intended full self-isolation in participants who had not experienced covid-19 symptoms in the past week. Participants were asked to imagine they developed symptoms of covid-19 (high temperature or fever, new continuous cough, or loss of sense of taste or smell) the next morning and what would cause them to leave home, if anything.

Requesting a test—Participants who reported covid-19 symptoms were asked what actions they had taken when symptoms developed. Response options included "I requested a test to confirm whether I have coronavirus." In data collected between 1 June and 5 August 2020 (waves 19 to 26), participants who reported requesting a test after symptoms had developed were asked whether the test indicated they had or did not have covid-19 or if they were still waiting for the test results. Participants who reported no covid-19 symptoms were asked what actions they would take if they were to develop symptoms.

Sharing details of close contacts—Participants who had not experienced covid-19 symptoms in the past seven days were asked to imagine they had tested positive for covid-19 and had been prompted by the National Health Service contact tracing service. We asked participants how likely they would then be to share details of people they had been in close contact with on a 5 point scale from "definitely would" to "definitely would not." We recoded intention to share details of close contacts into a binary variable (probably or definitely would share details versus not sure, probably, or definitely would not). Too few participants indicated that they had tested positive to analyse separately.

Personal and clinical characteristics

We asked participants to report their age, sex, employment status, ~~socioeconomic grade,~~ highest educational or professional qualification, ethnicity, and marital status, and the number of people living in their household. Participants also reported the occupation of the highest earner in the household, whether a dependent child lived in the household, they or a household member had a chronic illness, they worked in a key sector, or they were self-employed. Participants were asked for their full postcode, from which we determined region and indices of multiple deprivation.[21]

We coded participants as having a chronic illness that made them clinically vulnerable to covid-19 using guidance from the NHS website.[22] Participants were categorised as working in a key sector if they worked in one of several sectors specified in government guidance.[23]

Participants were asked if they thought they "had, or currently have, coronavirus." Those who reported having experienced symptoms of covid-19 in the past week were asked what they thought had caused their symptoms (symptom attribution). We measured financial hardship by asking participants to what extent in the past seven days they had been struggling to make ends meet, skipping meals, and finding their current living situation difficult (Cronbach's α=0.75).

Power calculation

We determined that a sample size of 2000 in each survey would allow a 95% confidence interval of plus or minus 2% for the prevalence estimate for a survey item with a prevalence of around 50%. In practice, power was considerably better as we pooled data from survey waves.

Statistical analysis

Owing to an error in collecting data about chronic illness on 26 to 28 October 2020 (wave 31), these data were excluded from analyses investigating factors associated with outcome variables. Responder IDs were not assigned for 4.3% of participants (n=6381/149 640). These responses were also excluded from analyses. We used generalised estimating equations (with an exchangeable correlation structure) to correct for some participants being in more than one wave. Generalised estimating equations were used to investigate factors associated with identifying cough, high temperature or fever, and loss of sense of smell or taste (25 May 2020 to 27 January 2021, excluding data collected 26-38 October 2020; wave 31), full self-isolation (14 April 2020 to 27 January 2021, excluding data collected 26-38 October; wave 31), duration adjusted adherence to full self-isolation (9 November 2020 to 27 January 2021), requesting a test (25 May 2020 to 27 January 2021, excluding data collected 26-38 October; wave 31), and intention to share details of close contacts if a covid-19 test result was positive (1 June 2020 to 27 January 2021, excluding data collected 26-38 October 2020; wave 31).

Between 14 April 2020 and 27 January 2021, excluding data collected from 26 to 28 October 2020 (wave 31) and those for whom a unique response ID was not assigned (ie, ~~(waves~~data points included in generalised estimating equations analyses), there were 59 237 responses from

40-112 participants. Overall, 30~~8~~ 25~~66~~7 participants (75.4%) answered one survey and 985~~706~~5 participants (~~15.~~24.6%) answered more than one survey (see supplementary file for numbers of responses and participants included in each analysis).

For each set of analyses, we ran univariable and multivariable analyses. Multivariable regressions adjusted for survey wave, region (with East Midlands arbitrarily allocated as reference category), sex, age (raw and quadratic term), a dependent child in the household, being clinically vulnerable to covid-19, having a household member with a chronic illness, employment status (working *v* not working), highest earner works in a manual occupation ~~socioeconomic grade~~ (~~ABC1 (high)~~no *v* yes~~C2DE~~),[24] index of multiple deprivation (fourths), highest educational or professional qualification (degree or higher *v* less than degree), ethnicity (white British (reference category), white other, mixed, Asian or Asian British, black or black British, Arab or other, don't know or prefer not to say), and living alone. Loess plots of age effects suggested quadratic relations would be appropriate.

Only participants who reported covid-19 symptoms in the past week were included in analyses of full self-isolation (n=339~~45~~7 responses; see supplementary file); duration adjusted self-isolation (n=1102~~3~~ responses) and requesting a test (n=2920~~58~~ responses).

It wa~~i~~s permissible to leave home during the self-isolation period to get tested or if a covid-19 test result wa~~i~~s negative.[25] For self-isolation analyses, we excluded those who reported a negative test result in one of several closed questions or in free text since their symptoms developed or in the past week (see supplementary file).

In analyses of factors associated with self-reported self-isolation and requesting a test, we recoded ethnicity into three categories owing to small numbers of cases: white British (reference category);~~,~~ white other;~~,~~ and black, Asian, mixed, or other (people who preferred not to say were excluded). For analyses of factors associated with self-reported self-isolation accounting for duration of isolation, we also merged participants in the north east and north west and participants in Scotland, Wales, and Northern Ireland into single groups.

Many analyses were conducted on each outcome variable (about 47). Uncorrected P values are given shown in the table; we only report narratively on results that remained statistically significant after applying a conservative Bonferroni correction (P<0.001).

Before the analyses reported here, we analysed survey results at multiple time points. Results were reported regularly to the Department of Health and Social Care and the UK Scientific Advisory Group for Emergencies (SAGE).

Sensitivity analysis

Socioeconomic grade can be derived from the question asking the occupation of the highest earner in the household (see supplementary file). We conducted a sensitivity analysis for adjusted generalised estimating equations, including socioeconomic grade as an explanatory variable and removing highest earner working in a manual occupation.

Patient and public involvement

Lay members served on the advisory group for the project that developed our prototype survey material; this included three rounds of qualitative testing.[26] Owing to the rapid nature of this research during the covid-19 pandemic, the public was not involved in further developments of the materials.

Results

Identification of covid-19 symptoms

When data from 26 May 2020 to 27 January 2021 (waves 18-42) were combined, 51.5% of participants (95% confidence interval 51.0% to 51.9%, n= 26 030/50 570) identified cough, high temperature or fever, and loss of sense of smell or taste as symptoms of covid-19. Recognition initially increased at the start of data collection and when loss of sense of smell or taste was introduced into government guidance,[20] after which it remained relatively stable (fig 1). In the latest available wave of data collection (wave 42, 25-27 January 2021), 50.8% (48.6% to 53.0%, n=1019/2007) of participants identified the symptoms of cough, high temperature or fever, and loss of sense of smell or taste. When analysis was restricted to recognition of cough and high temperature or fever alone, the results were similar. The supplementary file presents rates of recognition for individual symptoms.

Fig 1 Percentage of people who correctly identified the most common symptoms of covid-19. Error bars are 95% confidence intervals

Correct identification of covid-19 symptoms was associated with being female, older (see supplementary file), identifying as white British, a belief of not having had covid-19, lesser financial hardship, highest earner not working in a manual occupationsocioeconomic grade, living in less deprived areas, no dependent child in the household, not living alone, and not working in key sectors (table 1). Those who lived in London were less likely to identify symptoms of covid-19 (adjusted odds ratio 0.76, 95% confidence interval 0.69 to 0.843, compared with the baseline region, East Midlands; see supplementary file). Variation by survey wave was significant, although no individual wave reached our significance level.

Table 1 Associations between personal and clinical characteristics and correctly identifying high temperature or fever, cough, and loss of sense of smell or taste as main symptoms of covid-19

**Characteristics**	**Identification of covid-19 symptoms**		**Odds ratio (95% CI) for correct identification**	**P value**	**Adjusted odds ratio (95% CI) for correct identification***	**P value**	**Adjusted odds ratio (95% CI) for correct identification†**	**P value**
	**Not correctly identified (n=23 440638)**	**Correctly identified (n=24 72889)**						
Survey wave overall	-	-	χ^2(234)=104.015.6	<0.001	χ^2(23)=79.61	<0.001	χ^2(23)=80.5	<.001
Region overall	-	-	χ^2(11)=2613.89	<0.001	χ^2(11)=658.1	<0.001	χ^2(11)=61.8	<.001
Male	12 099 (55.0)12 229 (55.1)	9897 (45.0)9963 (44.9)	Reference	-	Reference	-	Reference	-
Female	11 267 (43.3)11 333 (43.3)	14 766 (56.7)14 861 (56.7)	1.6254 (1.5648 to 1.609)	<0.001	1.768 (1.6971 to 1.846)	<0.001	1.77 (1.69 to 1.84)	<0.001
Raw age (per decade) (years)	Mean 45.4 (SD 17.7)	Mean 50.9 (SD 17.0)	1.20 (1.198 to 1.21)	<0.001	1.17 (1.15 to 1.19)	<0.001	1.17 (1.15 to 1.18)	<0.001
Age: quadratic (age−mean)^2	-	-	-	-	0.9997 (0.9996 to 0.99987)	<0.001	0.99965 (0.99958 to 0.99973)	<0.001
Dependent child in household:								
No	15 299 (46.3)15 161 (46.0)	17 732 (53.7)17 800 (54.0)	Reference	-	Reference	-	Reference	-
Yes	8141 (53.8)8477 (54.5)	6996 (46.2)7089 (45.5)	0.726 (0.6972 to 0.759)	<0.001	0.90 (0.86 to 0.95)0.87 (0.83 to 0.91)	<0.001	0.90 (0.86 to 0.95)	<0.001

Clinically vulnerable to covid-19:								
No	17 941 (48.6)18 613 (49.0)	18 983 (51.4)19 356 (51.0)	Reference	-	Reference	-	Reference	-
Yes	4325 (46.2)4373 (46.3)	5043 (53.8)5072 (53.7)	1.0811 (1.053 to 1.164)	<0.001	0.96 (0.92 to 1.01)0.98 (0.93 to 1.03)	0.1536	0.98 (0.93 to 1.03)	0.42
Household member has chronic illness:								
No	19 024 (48.6)19 193 (48.6)	20 148 (51.4)20 276 (51.4)	Reference	-	Reference	-	Reference	-
Yes	3767 (47.7)3793 (47.7)	4123 (52.3)4152 (52.3)	1.03 (0.98 to 1.089)	0.226	0.99 (0.93 to 1.04)1.00 (0.94 to 1.05)	0.690	1.00 (0.95 to 1.06)	1.00
Employed:								
No	9988 (46.3)10 039 (46.2)	11 600 (53.7)11 669 (53.8)	Reference	-	Reference	-	Reference	-
Yes	13 048 (50.3)13 186 (50.4)	12 890 (49.7)12 979 (49.6)	0.881 (0.785 to 0.9284)	<0.001	1.078 (1.032 to 1.13)	0.0032	1.00 (0.96 to 1.05)	0.91
Socioeconomic gradeHighest earner works in a manual occupation‡‡:								
ABC1 (high)No	15 541 (46.4)15 699 (46.5)	17 967 (53.6)18 087 (53.5)	Reference	-	Reference	-	-	-
C2DEYes	7389 (54.1)7428 (54.1)	6273 (45.9)6309 (45.9)	0.73 (0.70 to 0.776)	<0.001	0.82 (0.78 to 0.86)	<0.001	-	-
Socioeconomic grade:								
ABC1 (high)	11 995 (45.4)	14 400 (54.6)	Reference	-	-	-	Reference	-

C2DE	10 935 (52.6)	9840 (47.4)	0.74 (0.72 to 0.77)	<0.001	-	-	0.76 (0.73 to 0.80)	<0.001
Index of multiple deprivation fourth:								
1st (least deprived)	4518 (43.4)4544 (43.4)	5883 (56.6)5919 (56.6)	1.579 (1.4950 to 1.686)	<0.001	1.267 (1.198 to 1.354)	<0.001	1.22 (1.14 to 1.29)	<0.001
2nd	5256 (45.4)5319 (45.5)	6313 (54.6)6361 (54.5)	1.44 (1.367 to 1.52)	<0.001	1.19 (1.132 to 1.267)	<0.001	1.16 (1.10 to 1.24)	<0.001
3rd	6373 (49.7)6422 (49.8)	6445 (50.3)6484 (50.2)	1.212 (1.15 to 1.28)	<0.001	1.110 (1.045 to 1.167)	<0.001	1.09 (1.03 to 1.15)	0.005
4th (most deprived)	7293 (54.5)7353 (54.6)	6087 (45.5)6125 (45.4)	Reference	-	Reference	-	Reference	-
Overall	-	-	$\chi^2(3)$=302.518.7	<0.001	$\chi^2(3)$=59.063.7	<0.001	$\chi^2(3)$=43.0	<.001
Highest educational or professional qualification:								
GCSE, vocational, A level, or no formal qualifications	15 395 (48.5)15 489 (48.5)	16 337 (51.5)16 438 (51.5)	Reference	-	Reference	-	Reference	-
Degree or higher (bachelors, masters, or PhD)	8045 (48.9)8149 (49.1)	8391 (51.1)8451 (50.9)	0.98 (0.945 to 1.032)	0.425	1.06 (1.02 to 1.11)	0.009	1.03 (0.99 to 1.08)	0.19
Ethnicity:								
White British	18 616 (46.2)18 780 (46.2)	21 722 (53.8)21 857 (53.8)	Reference	-	Reference	-	Reference	-
White other	1949 (59.8)1961 (59.8)	1310 (40.2)1316 (40.2)	0.56 (0.52 to 0.61)	<0.001	0.6970 (0.643 to 0.765)	<0.001	0.69 (0.63 to 0.75)	<0.001
Mixed	656 (63.1)661 (63.1)	384 (36.9)387 (36.9)	0.501 (0.445 to 0.58)	<0.001	0.63 (0.54 to 0.72)	<0.001	0.63 (0.55 to 0.73)	<0.001
Asian or Asian British	1327 (61.4)1340 (61.2)	836 (38.6)851 (38.8)	0.55 (0.50 to 0.601)	<0.001	0.74 (0.667 to 0.82)	<0.001	0.73 (0.66 to 0.81)	<0.001
Black or black British	618 (68.3)620 (68.3)	287 (31.7)288 (31.7)	0.40 (0.34 to 0.476)	<0.001	0.54 (0.46 to 0.64)	<0.001	0.54 (0.45 to 0.64)	<0.001
Arab or other	104 (55.9)104 (55.9)	82 (44.1)82 (44.1)	0.67 (0.50 to 0.91)	0.01	0.78 (0.576 to 1.09)	0.15	0.77 (0.56 to 1.07)	0.12
Don't know or prefer not to say	170 (61.4)172 (61.4)	107 (38.6)108 (38.6)	0.57 (0.44 to 0.743)	<0.001	0.845 (0.60 to 1.1820)	0.362	0.86 (0.61 to 1.20)	0.37

Overall	-	-	$\chi^2(6)$=534.0~~50.4~~	<0.001	$\chi^2(6)$=161~~2~~.~~4~~0	<0.001	$\chi^2(6)$=165.6	<.001
Live alone:								
No	18 830 (48.8)~~19 002 (48.9)~~	19 740 (51.2)~~19 879 (51.1)~~	Reference	-	Reference	-	Reference	-
Yes	4610 (48.0)~~4636 (48.1)~~	4988 (52.0)~~5010 (51.9)~~	1.01~~0~~ (0.96 to 1.0~~5~~6)	0.~~8~~69	0.8~~5~~7 (0.8~~1~~2 to 0.9~~0~~2)	<0.001	0.89 (0.85 to 0.95)	<0.001
Work in key sector:								
No	6224 (49.7)~~6283 (49.7)~~	6312 (50.3)~~6364 (50.3)~~	Reference	-	Reference	-	Reference	-
Yes	8424 (52.4)~~8515 (52.5)~~	7657 (47.6)~~7705 (47.5)~~	0.89 (0.85 to 0.93)	<0.001	0.9~~0~~1 (0.8~~5~~6 to 0.9~~5~~6)	<0.001	0.91 (0.86 to 0.96)	<0.001
Self-employed§‡:								
No	12 147 (50.3)~~12 283 (50.4)~~	11 981 (49.7)~~12 065 (49.6)~~	Reference	-	Reference	-	Reference	-
Yes	901 (49.8)~~903 (49.7)~~	909 (50.2)~~914 (50.3)~~	0.99 (0.~~8~~90 to 1.10~~9~~)	0.~~7~~85	0.92~~3~~ (0.83 to 1.03)	0.15~~7~~	0.93 (0.83 to 1.04)	0.20
Marital status:								
Single, separated, divorced, or widowed	9757 (51.3)~~9804 (51.3)~~	9271 (48.7)~~9323 (48.7)~~	Reference	-	Reference	-	Reference	-
Married or partnered	13 286 (46.4)~~13 430 (46.5)~~	15 318 (53.6)~~15 427 (53.5)~~	1.23 (1.18 to 1.28)	<0.001	1.07 (1.02 to 1.13)	0.01	1.06 (1.01 to 1.12)	0.03
Ever had covid-19:								
Think not	19 242 (46.6)~~19 357 (46.6)~~	22 019 (53.4)~~22 168 (53.4)~~	Reference	-	Reference	-	Reference	-
Think so, or confirmed	4198 (60.8)~~4281 (61.1)~~	2709 (39.2)~~2721 (38.9)~~	0.60~~58~~ (0.57~~5~~ to 0.6~~1~~3)	<0.001	0.71~~69~~ (0.65~~7~~ to 0.7~~3~~5)	<0.001	0.71 (0.67 to 0.75)	<0.001
Hardship¶~~§~~	n=22 323~~510~~; mean 8.4 (SD 3.0)	n=234 ~~089~~32; mean 7.4 (SD 2.8)	0.89~~3~~6 (0.8~~87~~90 to 0.~~899~~02)	<0.001	0.92~~4~~7 (0.920~~17~~ to 0.934~~1~~)	<0.001	0.93 (0.92 to 0.94)	<0.001

SD=standard deviation; CI=confidence interval. For continuous variables, odds ratios represent a one unit increase in the explanatory variable, apart from age, when odds ratios represent a 10 year increase in age.

*Adjusted for survey wave, region, sex, age (raw and quadratic term), dependent child in household, clinically vulnerable to covid-19, household member has chronic illness, employment status, highest earner works in a manual occupationsocioeconomic grade, index of multiple deprivation, highest educational or professional qualification, ethnicity, and living alone.
†Adjusting for survey wave, region, sex, age (raw and quadratic term), dependent child in the household, clinically vulnerable to covid-19, household member has chronic illness, employment status, socioeconomic grade, index of multiple deprivation, highest educational or professional qualification, ethnicity, and living alone.
‡†For most analyses, an exchangeable correlation structure was used—this failed to converge for the univariable analysis for region, so an unstructured correlation structure was used.
§‡Not adjusted for employment status as by definition all people who were asked whether they were self-employed were working.
¶§From 3 (least hardship) to 15 (most hardship).

Results did not differ in a sensitivity analysis adjusting for socioeconomic grade rather than the highest earner being a manual worker (table 1 and supplementary file).

Fully self-isolating when symptomatic

Combining data from 14 April 2020 to 27 January 2021 (waves 12 to 42), of those who reported having experienced symptoms of covid-19 in the past seven days (excluding those who reported receiving a negative covid-19 test result since having developed symptoms), only 20.2% (95% confidence interval 18.8% to 21.5%, n=720/3567) said they had not left home since developing symptoms. The percentage of people who reported full self-isolation was largely stable until October 2020 and then increased (fig 2). In the latest wave of data collection (wave 42, 25-27 January 2021), the percentage of people who reported not leaving home after symptoms developed was 31.3% (21.1% to 41.5%, n=26/83). From 26 October 2020 to 27 January 2021 (waves 31 to 42), duration adjusted adherence to full self-isolation was 42.5% (39.7% to 45.2%, n=515/1213). In the latest wave of data collection (wave 42, 25-27 January 2021), duration adjusted adherence was 51.8% (40.8% to 62.8%, n=43/83). Intention to fully self-isolate if symptoms of covid-19 were to develop was much higher, at around 70%, and was 71.0% (68.9% to 73.0%, n=1341/1890) in the latest wave of data collection (wave 42).

Fig 2 Percentage of people who reported not leaving home since developing symptoms of covid-19 (in those who had experienced covid-19 symptoms in the past seven days, excluding those who had received a negative covid-19 test result since developing symptoms) and who reported no intention to leave home if they were to develop covid-19 symptoms (in people who had not had covid-19 symptoms in the past seven days). Prevalence estimates, using the Office for National Statistics (ONS) covid-19 survey are also included. Error bars are 95% confidence intervals

No associations between duration adjusted self-isolation and any personal or clinical characteristic were significant after applying a conservative Bonferroni correction (table 2).

Table 2 Associations between personal and clinical characteristics and full self-isolation adjusted for duration of isolation after developing symptoms of covid-19

**Characteristics**	**Self-isolating status**		**Odds ratio (95% CI) for fully self-isolating**	**P value**	**Adjusted odds ratio (95% CI) for fully self-isolating***	**P value**	**Adjusted odds ratio (95% CI) for fully self-isolating†**	**P value**
	**Not fully self-isolating (n=648)**	**Fully self-isolating (n=4554)**						
Survey wave overall	-	-	χ^2(101)=14.09.2	0.1706	χ^2(10)=167.58	0.086	χ^2(10)=16.8	0.08
Region overall	-	-	χ^2(8)=2.53.0	0.964	χ^2(8)=2.97	0.945	χ^2(8)=2.9	0.94
Male	376 (62.9)381 (63.1)	222 (37.1)223 (36.9)	Reference	-	Reference	-	Reference	-
Female	269 (54.3)264 (53.9)	226 (45.7)226 (46.1)	1.416 (1.150 to 1.807)	0.0062	1.502 (1.147 to 1.979)	0.0042	1.49 (1.13 to 1.97)	0.004
Raw age (per decade) (years)	Mean 36.67 (SD 14.4)	Mean 39.76 (SD 16.1)	1.143 (1.045 to 1.242)	0.0023	1.163 (1.014 to 1.3026)	0.0073	1.16 (1.04 to 1.30)	0.007
Age: quadratic (age−mean)^2	-	-	-	-	0.9999 (0.9994 to 1.0004)	0.678	0.9999 (0.9994 to 1.0004)	0.66
Dependent child in household:								
No	281 (54.4)257 (53.0)	236 (45.6)228 (47.0)	Reference	-	Reference	-	Reference	-
Yes	367 (62.7)391 (63.3)	218 (37.3)227 (36.7)	0.7166 (0.526 to 0.9184)	0.0071	0.883 (0.624 to 1.121)	0.4322	0.88 (0.64 to 1.21)	0.43
Clinically vulnerable to covid-19:								
No	428 (59.0)464 (58.8)	297 (41.0)325 (41.2)	Reference	-	Reference	-	Reference	-
Yes	167 (58.2)167 (58.4)	120 (41.8)119 (41.6)	1.013 (0.778 to 1.337)	0.8194	0.932 (0.689 to 1.25)	0.5962	0.92 (0.67 to 1.25)	0.58
Household member has chronic illness:								
No	513 (58.3)513 (58.3)	367 (41.7)367 (41.7)	Reference	-	Reference	-	Reference	-
Yes	118 (60.8)118 (60.5)	76 (39.2)77 (39.5)	0.8890 (0.664 to 1.241)	0.452	0.7984 (0.548 to 1.1520)	0.2133	0.79 (0.54 to 1.14)	0.21
Employed:								
No	196 (54.9)198 (55.0)	161 (45.1)162 (45.0)	Reference	-	Reference	-	Reference	-

Yes	446 (61.3)444 (61.3)	281 (38.7)280 (38.7)	0.798 (0.610 to 1.002)	0.075	0.885 (0.641 to 1.1720)	0.341	0.84 (0.61 to 1.17)	0.31
Highest earner works in a manual occupationSocioeconomic grade:								
ABC1 (high)No	343 (57.4)342 (57.3)	255 (42.6)255 (42.7)	Reference	-	Reference	-	-	-
C2DEYes	295 (61.0)296 (60.9)	189 (39.0)190 (39.1)	0.86 (0.678 to 1.10)	0.22	0.957 (0.723 to 1.294)	0.8569	-	-
Socioeconomic grade:								
ABC1 (high)	286 (59.8)	192 (40.2)	Reference	-	-	-	Reference	-
C2DE	352 (58.3)	252 (41.7)	1.06 (0.83 to 1.35)	0.65	-	-	1.00 (0.76 to 1.33)	0.99
Index of multiple deprivation fourth:								
1st (least deprived)	89 (59.3)88 (59.1)	61 (40.7)61 (40.9)	1.07 (0.73 to 1.567)	0.752	0.892 (0.596 to 1.415)	0.610.73	0.89 (0.56 to 1.41)	0.61
2nd	111 (52.1)111 (52.1)	102 (47.9)102 (47.9)	1.435 (1.042 to 2.013)	0.043	1.3942 (0.937 to 2.068)	0.110.07	1.39 (0.93 to 2.06)	0.11
3rd	189 (60.4)189 (60.4)	124 (39.6)124 (39.6)	1.023 (0.76 to 1.38)	0.886	0.895 (0.683 to 1.2734)	0.530.78	0.89 (0.63 to 1.27)	0.53
4th (most deprived)	259 (60.8)260 (60.7)	167 (39.2)168 (39.3)	Reference	-	Reference	-	Reference	-
Overall	-	-	$\chi^2(3)$=4.95.3	0.185	$\chi^2(3)$=5.75	0.134	$\chi^2(3)$=5.6	0.13
Highest educational or professional qualification:								
GCSE, vocational, A level, or no formal qualifications	357 (57.4)358 (57.4)	265 (42.6)266 (42.6)	Reference	-	Reference	-	Reference	-
Degree or higher (bachelors, masters, or PhD)	291 (60.6)290 (60.5)	189 (39.4)189 (39.5)	0.887 (0.69 to 1.12)	0.28	0.97 (0.734 to 1.289)	0.843	0.97 (0.73 to 1.29)	0.85
Ethnicity:								
White British	455 (59.2)454 (59.1)	314 (40.8)314 (40.9)	Reference	-	Reference	-	Reference	-
White other	90 (65.2)90 (65.2)	48 (34.8)48 (34.8)	0.78 (0.534 to 1.142)	0.2018	0.85 (0.55 to 1.31)0.89 (0.59 to 1.36)	0.450.60	0.84 (0.55 to 1.30)	0.44
Black and minority ethnicity	101 (53.7)102 (53.7)	87 (46.3)88 (46.3)	1.264 (0.901 to 1.741)	0.169	1.66 (1.14 to 2.41)1.54 (1.07 to 2.2)	0.0080.02	1.66 (1.14 to 2.41)	0.008

Overall	-	-	$\chi^2(2)$=4.3	0.12	$\chi^2(2)$=8.8~~6.6~~	0.01~~4~~	$\chi^2(2)$=8.8	0.01
Live alone:								
No	549 (59.3)~~550 (59.2)~~	377 (40.7)~~379 (40.8)~~	Reference	-	Reference	-	Reference	-
Yes	99 (56.3)~~98 (56.3)~~	77 (43.8)~~76 (43.7)~~	1.12 (0.81 to 1.56)	0.48~~9~~	0.95~~6~~ (0.6~~6~~3 to 1.44~~1~~)	0.82~~4~~	0.95 (0.63 to 1.45)	0.82
Work in key sector:								
No	121 (54.5)~~119 (54.3)~~	101 (45.5)~~100 (45.7)~~	Reference	-	Reference	-	Reference	-
Yes	382 (63.2)~~383 (63.3)~~	222 (36.8)~~222 (36.7)~~	0.70~~68~~ (0.5~~0~~1 to 0.95~~4~~)	0.02	0.83~~7~~ (0.58~~61~~ to 1.2~~5~~0)	0.33~~46~~	0.83 (0.58 to 1.20)	0.32
Self-employed‡~~‡~~:								
No	410 (61.5)~~408 (61.4)~~	257 (38.5)~~257 (38.6)~~	Reference	-	Reference	-	Reference	-
Yes	36 (60.0)~~36 (61.0)~~	24 (40.0)~~23 (39.0)~~	1.0~~3~~8 (0.6~~0~~3 to 1.~~7~~85)	0.78~~91~~	1.31 (0.66 to 2.61)~~1.08 (0.56 to 2.11)~~	0.44~~0.81~~	1.32 (0.66 to 2.62)	0.43
Marital status:								
Single, separated, divorced, or widowed	261 (56.6)~~263 (56.8)~~	200 (43.4)~~200 (43.2)~~	Reference	-	Reference	-	Reference	-
Married or partnered	365 (60.1)~~363 (59.9)~~	242 (39.9)~~243 (40.1)~~	0.86~~9~~ (0.68~~9~~ to 1.11~~3~~)	0.25~~34~~	0.87 (0.64 to 1.19)~~0.94 (0.69 to 1.27)~~	0.38~~0.69~~	0.87 (0.64 to 1.19)	0.39
Ever had covid-19:								
Think not	371 (57.8)~~368 (57.5)~~	271 (42.2)~~272 (42.5)~~	Reference	-	Reference	-	Reference	-
Think so, or confirmed	277 (60.2)~~280 (60.5)~~	183 (39.8)~~183 (39.5)~~	0.~~8~~91 (0.71~~0~~ to 1.16~~4~~)	0.44~~37~~	1.0~~3~~2 (0.7~~8~~7 to 1.34)	0.90~~85~~	1.02 (0.77 to 1.35)	0.90
Attribute current symptoms to covid-19:								
No	494 (60.7)~~493 (60.6)~~	320 (39.3)~~321 (39.4)~~	Reference	-	Reference	-	Reference	-
Yes	154 (53.5)~~155 (53.6)~~	134 (46.5)~~134 (46.4)~~	1.33 (1.02 to 1.7~~5~~4)	0.04	1.49 (1.1~~2~~0 to 2.02~~0~~)	0.01~~07~~	1.49 (1.10 to 2.02)	0.01
Hardship§~~‡~~	n=622; mean 10.2~~3~~ (SD 2.6)	n=42~~8~~7; mean 10.0 (SD 2.9)	0.97 (0.93 to 1.01)	0.1~~8~~7	0.99 (0.9~~4~~3 to 1.04)	0.60~~8~~	0.99 (0.93 to 1.04)	0.60

SD=standard deviation; CI=confidence interval. For continuous variables, odds ratios represent a one unit increase in the explanatory variable, apart from age, when odds ratios represent a 10 year increase in age.

*Adjusted for survey wave, region, sex, age (raw and quadratic term), dependent child in household, clinically vulnerable to covid-19, household member has chronic illness, employment status, highest earner works in a manual occupation~~socioeconomic grade~~, index of multiple deprivation, highest educational or professional qualification, ethnicity, and living alone.
†Adjusting for survey wave, region, sex, age (raw and quadratic term), dependent child in the household, clinically vulnerable to covid-19, household member has chronic illness, employment status, socioeconomic grade, index of multiple deprivation, highest educational or professional qualification, ethnicity, and living alone.
‡~~†~~Not adjusted for employment status as by definition all people who were asked whether they were self-employed were working.
~~‡~~§From 3 (least hardship) to 15 (most hardship).

Results did not differ in a sensitivity analysis adjusting for socioeconomic grade rather than the highest earner being a manual worker (table 2 and supplementary file).

Adherence to full self-isolation was associated with not working in a key sector (working in a key sector: adjusted odds ratio 0.5~~0~~1, 95% confidence interval 0.39 to 0.6~~6~~8), thinking you had not experienced covid-19 (thinking you had experienced covid-19 or covid-19 had been confirmed: 0.59~~8~~, 0.4~~7~~8 to 0.7~~1~~3), ~~not having a dependent child in the household (having a dependent child in the household: 0.61, 0.49 to 0.75),~~ female sex (1.87~~92~~, 1.5~~8~~3 to 2.29~~33~~), older age (1.2~~7~~8, 1.1~~8~~9 to 1.3~~6~~8), low~~poor~~er education (degree or higher: 0.61~~3~~, 0.5~~2~~0 to 0.74~~7~~), highest earner not working in a manual occupation ~~higher socioeconomic grade~~ (highest earner works in a manual occupation: 0.6~~6~~9, 0.5~~4~~6 to 0.8~~1~~4), and lesser financial hardship (0.91, 0.87 to 0.95~~4~~; see supplementary file).

When including socioeconomic grade as an explanatory variable, rather than highest earner working in a manual occupation, the association between full self-isolation and lower socioeconomic grade (C2DE) did not reach the threshold for significance (supplementary file). However, adjusted odds ratios were similar for highest earner working in a manual occupation and lower socioeconomic grade.

When data from 26 October 2020 to 27 January 2021 (waves 31 to 42) were combined, the most frequently reported reasons for not fully self-isolating were to go to the shops for groceries or to a pharmacy (21.5%), to go to work (15.8%), to go to the shops for things other than groceries or pharmacy goods (15.6%), because symptoms did not persist or were temporary (15.2%), to go out for a medical need other than covid-19 (15.0%), to go for a walk or for some other exercise (14.8%), believing symptoms were only mild (14.5%), because symptoms got better (13.9%), thinking it was not necessary to stay at home (13.2%), being too bored (12.2%), to help or provide care for a vulnerable person (11.9%), to meet up with friends or family, or both (11.3%), and being too depressed or anxious (11.2%; see supplementary file).

Requesting a test when symptomatic

When data from 26 May 2020 to 27 January 2021 (waves 18 to 42) were combined, of those who reported experiencing covid-19 symptoms in the past seven days, only 18.0% (95% confidence interval 16.6% to 19.3%, n=552/3068) reported requesting a test. In the latest wave of data collection (wave 42, 25-27 January 2021), the percentage of people requesting a test after symptoms developed was 22.2% (14.6% to 29.9%, n=26/117). Self-reported behaviour and intention to request a test when symptomatic increased over time. In the latest wave of data collection (wave 42, 25-27 January 2021), intention to request a test when symptomatic was 62.3% (60.1% to 64.5%, n=1178/1890) (fig 3).

Fig 3 Percentage of people who reported requesting a test after developing covid-19 symptoms (in those who had experienced covid-19 symptoms in the past seven days), and who reported intending to request a test if they were to develop covid-19 symptoms (in people who had not had covid-19 symptoms in the past seven days). Pillar 2 testing capacity is also included.[27] Error bars are 95% confidence intervals

Survey waves varied considerably. Participants in later waves were more likely to report requesting a test when symptomatic compared with those in wave 18 (see supplementary file). Requesting a test for covid-19 was associated with people thinking that their current symptoms could be due to covid-19 (adjusted odds ratio 1.81~~73~~, 95% confidence interval 1.42~~37~~ to 2.31~~9~~) and ~~being a woman~~lesser financial hardship (0.93~~1.47~~, 0.89~~1.20~~ to 0.97~~1.81~~; see supplementary file).

Results did not differ in sensitivity analyses adjusting for socioeconomic grade (see supplementary file).

Self-reported reasons for not requesting a test were included from 8 June 2020 (wave 20). When data from 8 June 2020 to 27 January 2021 (wave 42) were combined, the most common reasons for not requesting a test were thinking the symptoms were not due to covid-19 (20.9%), symptoms had improved (16.9%), symptoms were only mild (16.3%), having no contact with anyone who had covid-19 recently (13.0%), thinking that only self-isolation was needed (11.5%), not wanting to use a test that someone needed more (11.1%), not thinking you were eligible to get a test (11.0%), and being worried about how colleagues or employers would react if a test result was positive (10.0%; see supplementary file).

Sharing details of close contacts

When data from 1 June 2020 to 27 January 2021 (wave 19 to 42) were combined, of those who had not experienced covid-19 symptoms in the past seven days, 79.1% (78.8% to 79.5%, n=36 145/45 680) reported that they probably or definitely would share details of close contacts with the NHS contact tracing service if they tested positive for covid-19 and were prompted by the NHS contact tracing service (fig 4). Intention to share details of close contacts increased slightly over time. In the latest wave of data collection (wave 42, 25-27 January 2021), 81.9% (80.1% to 83.6%, n=1547/1890) intended to share details of close contacts.

Fig 4 Percentage of people who reported that they probably or definitely would share details of close contacts if contacted by the NHS contact tracing service (in people who had not had covid-19 symptoms in the past seven days). Error bars are 95% confidence intervals

Intending to share details of close contacts was associated with being female, older, living in less deprived areas, higher education, highest earner not working in a manual occupationhigher socioeconomic grade, being clinically vulnerable to covid-19, being married or partnered, working, thinking that you had previously had covid-19, not living alone, and lesser financial hardship (table 3). Not intending to share details of close contacts was associated with preferring not to disclose ethnicity. Survey waves varied considerably, with participants showing greater intention to share details of close contacts in later waves (see supplementary file).

Table 3 Associations between personal and clinical characteristics and intending to share details of close contacts with the NHS contact tracing service

**Characteristics**	**Intention to share details of close contacts**		**Odds ratio (95% CI) for sharing details**	**P value**	**Adjusted odds ratio (95% CI) for sharing details***	**P value**	**Adjusted odds ratio (95% CI) for sharing details†**	**P value**
	**Probably or definitely would not or not sure (n=913895)**	**Probably or definitely would (n=34 55299)**						
Survey wave overall	-	-	χ^2(232)=132.705.0	<0.001	χ^2(22)=884.34	<0.001	χ^2(22)=89.4	<0.001
Region overall	-	-	χ^2(11)=85.93.2	<0.001	χ^2(11)=23.57.3	0.0204	χ^2(11)=24.2	.01
Male	4603 (23.5)4628 (23.4)	15 013 (76.5)15 146 (76.6)	Reference	-	Reference	-	Reference	-
Female	4491 (19.0)4522 (19.0)	19 207 (81.0)19 327 (81.0)	1.287 (1.221 to 1.354)	<0.001	1.398 (1.312 to 1.476)	<0.001	1.40 (1.32 to 1.48)	<0.001

Raw age (per decade) (years)	Mean 44.5 (SD 16.~~1~~2)	Mean 50.3 (SD 17.5)	1.21 (1.19 to 1.23~~2~~)	<0.001	1.23 (1.21 to 1.26)	<0.001	1.23 (1.20 to 1.25)	<0.001
Age: quadratic (age−mean)^2	-	-	-	-	1.0006 (1.0005 to 1.0007)	<0.001	1.0006 (1.0005 to 1.0007)	<0.001
Dependent child in household:								
No	6084 (20.0)~~6076 (19.9)~~	24 410 (80.0)~~24 432 (80.1)~~	Reference	-	Reference	-	Reference	-
Yes	3054 (23.6)~~3119 (23.6)~~	9889 (76.4)~~10 120 (76.4)~~	0.80 (0.76 to 0.84~~5~~)	<0.001	1.00~~3~~ (0.9~~6~~4 to 1.07~~9~~)	1.0~~.4~~0	1.00 (0.94 to 1.07)	0.97
Clinically vulnerable to covid-19:								
No	7238 (21.5)~~7429 (21.6)~~	26 363 (78.5)~~27 004 (78.4)~~	Reference	-	Reference	-	Reference	-
Yes	1354 (16.4)~~1358 (16.4)~~	6884 (83.6)~~6942 (83.6)~~	1.39~~40~~ (1.3~~1~~0 to 1.49~~50~~)	<0.001	1.24~~5~~ (1.1~~7~~6 to 1.3~~5~~4)	<0.001	1.26 (1.17 to 1.36)	<0.001
Household member has chronic illness:								
No	7413 (20.9)~~7461 (20.9)~~	28 032 (79.1)~~28 238 (79.1)~~	Reference	-	Reference	-	Reference	-
Yes	1321 (18.9)~~1326 (18.9)~~	5663 (81.1)~~5708 (81.1)~~	1.~~1~~09 (1.02~~3~~ to 1.17~~8~~)	0.01~~04~~	1.02 (0.95 to 1.10)	0.58~~1~~	1.03 (0.96 to 1.11)	0.37
Employed:								
No	3741 (18.9)~~3757 (18.9)~~	16 049 (81.1)~~16 142 (81.1)~~	Reference	-	Reference	-	Reference	-
Yes	5157 (22.4)~~5192 (22.3)~~	17 904 (77.6)~~18 061 (77.7)~~	0.85 (0.81 to 0.89)	<0.001	1.20 (1.13 to 1.2~~7~~8)	<0.001	1.14 (1.07 to 1.21)	<0.001
Highest earner works in a manual occupation~~Socioeconomic grade~~:								
~~ABC1~~No	6048 (19.7)~~6090 (19.7)~~	24 633 (80.3)~~24 830 (80.3)~~	Reference	-	Reference	-	-	-
~~C2DE~~Yes	2876 (24.3)~~2890 (24.3)~~	8959 (75.7)~~9010 (75.7)~~	0.7~~8~~9 (0.74 to 0.83)	<0.001	0.8~~9~~8 (0.83~~4~~ to 0.94)	<0.001	-	-
Socioeconomic grade								
ABC1 (high)	4515 (18.7)	19 572 (81.3)	Reference	-	-	-	Reference	-
C2DE	4409 (23.9)	14 020 (76.1)	0.75 (0.71 to 0.79)	<0.001	-	-	0.79 (0.74 to 0.83)	<0.001
Index of multiple deprivation fourth:								
1st (least deprived)	1580 (16.5)~~1587 (16.5)~~	8004 (83.5)~~8053 (83.5)~~	1.69~~75~~ (1.57~~62~~ to 1.83~~9~~)	<0.001	1.~~41~~39 (1.28~~30~~ to 1.52~~3~~)	<0.001	1.35 (1.23 to 1.47)	<0.001
2nd	2042 (19.3)~~2055 (19.2)~~	8544 (80.7)~~8623 (80.8)~~	1.37~~40~~ (1.28~~31~~ to 1.47~~51~~)	<0.001	1.15~~6~~ (1.0~~7~~6 to 1.24~~5~~)	<0.001	1.12 (1.04 to 1.21)	0.004

3rd	2600 (22.6)~~2618 (22.6)~~	8926 (77.4)~~8984 (77.4)~~	1.13~~6~~ (1.0~~8~~5 to 1.21~~3~~)	<0.001	1.03~~4~~ (0.9~~7~~6 to 1.11~~2~~)	0.39~~28~~	1.02 (0.95 to 1.10)	0.61
4th (most deprived)	2916 (24.8)~~2935 (24.8)~~	8825 (75.2)~~8892 (75.2)~~	Reference	-	Reference	-	Reference	-
Overall	-	-	χ^2(3)=206.~~1~~9~~.4~~	<0.001	χ^2(3)=~~73.~~66.9	<0.001	χ^2(3)=54.3	<0.001
Highest educational or professional qualification:								
GCSE, vocational, A level, or no formal qualifications	6313 (21.8)~~6350 (21.8)~~	22 670 (78.2)~~22 804 (78.2)~~	Reference	-	Reference	-	Reference	-
Degree or higher (bachelors, masters, or PhD)	2825 (19.5)~~2845 (19.5)~~	11 629 (80.5)~~11 748 (80.5)~~	1.15~~8~~ (1.09~~11~~ to 1.21~~4~~)	<0.001	1.20~~3~~ (1.1~~5~~3 to 1.28~~31~~)	<0.001	1.16 (1.09 to 1.23)	<0.001
Ethnicity:								
White British	7360 (20.0)~~7406 (20.0)~~	29 472 (80.0)~~29 686 (80.0)~~	Reference	-	Reference	-	Reference	-
White other	663 (24.4)~~665 (24.4)~~	2053 (75.6)~~2066 (75.6)~~	0.78~~9~~ (0.7~~2~~1 to 0.87~~8~~)	<0.001	0.97~~6~~ (0.86 to 1.08)	0.54~~2~~	0.96 (0.86 to 1.08)	0.53
Mixed	227 (26.7)~~228 (26.6)~~	623 (73.3)~~629 (73.4)~~	0.66 (0.56~~7~~ to 0.77~~8~~)	<0.001	0.81 (0.68 to 0.97)	0.02	0.81 (0.68 to 0.97)	0.02
Asian or Asian British	483 (26.2)~~486 (26.0)~~	1364 (73.8)~~1383 (74.0)~~	0.75~~8~~ (0.66~~9~~ to 0.84~~8~~)	<0.001	0.98~~1.02~~ (0.86~~90~~ to 1.12~~7~~)	0.75~~2~~	0.97 (0.85 to 1.11)	0.64
Black or black British	232 (29.8)~~234 (30.0)~~	546 (70.2)~~547 (70.0)~~	0.55 (0.46 to 0.65)	<0.001	0.75 (0.62 to 0.91)	0.00~~2~~3	0.74 (0.61 to 0.90)	0.002
Arab or other	47 (28.8)~~47 (28.8)~~	116 (71.2)~~116 (71.2)~~	0.60~~2~~ (0.4~~2~~1 to 0.87~~90~~)	0.01	0.67~~71~~ (0.4~~7~~3 to 1.02~~8~~)	0.06~~11~~	0.65 (0.43 to 1.00)	0.05
Don’t know or prefer not to say	126 (50.2)~~129 (50.8)~~	125 (49.8)~~125 (49.2)~~	0.33~~4~~ (0.2~~6~~5 to 0.43~~5~~)	<0.001	0.31~~2~~ (0.22 to 0.4~~6~~4)	<0.001	0.31 (0.22 to 0.44)	<0.001
Overall	-	-	χ^2(6)=1~~5~~66.0~~1~~	<0.001	χ^2(6)=5~~4~~6.~~0~~1	<0.001	χ^2(6)=56.7	<0.001
Live alone:								
No	7209 (20.8)~~7255 (20.8)~~	27 425 (79.2)~~27 647 (79.2)~~	Reference	-	Reference	-	Reference	-
Yes	1929 (21.9)~~1940 (21.9)~~	6874 (78.1)~~6905 (78.1)~~	0.93 (0.88 to 1.00~~.99~~)	0.04~~3~~	0.81~~2~~ (0.76 to 0.88)	<0.001	0.83 (0.77 to 0.90)	<0.001
Work in key sector:								
No	2759 (24.0)~~2781 (24.0)~~	8737 (76.0)~~8812 (76.0)~~	Reference	-	Reference	-	Reference	-
Yes	2981 (21.6)~~2996 (21.5)~~	10 846 (78.4)~~10 946 (78.5)~~	1.12~~3~~ (1.06 to 1.20)	<0.001	1.11~~2~~ (1.04 to 1.19)	0.002~~1~~	1.11 (1.04 to 1.19)	0.003
Self-employed‡~~‡~~								

No	4778 (22.3)~~4811 (22.2)~~	16 689 (77.7)~~16 842 (77.8)~~	Reference	-	Reference	-	Reference	-
Yes	379 (23.8)~~381 (23.8)~~	1215 (76.2)~~1219 (76.2)~~	0.94 (0.83 to 1.07~~6~~)	0.34~~3~~	0.85~~3~~ (0.7~~3~~4 to 0.97~~5~~)	0.01~~08~~	0.85 (0.74 to 0.97)	0.02
Marital status:								
Single, separated, divorced, or widowed	4067 (23.8)~~4082 (23.8)~~	12 990 (76.2)~~13 061 (76.2)~~	Reference	-	Reference	-	Reference	-
Married or partnered	4921 (18.9)~~4958 (18.9)~~	21 053 (81.1)~~21 234 (81.1)~~	1.31~~3~~ (1.25~~6~~ to 1.38~~40~~)	<0.001	1.~~2~~19 (1.11~~3~~ to 1.28~~9~~)	<0.001	1.18 (1.10 to 1.26)	<0.001
Ever had covid-19:								
Think not	7958 (20.9)~~8009 (20.9)~~	30 056 (79.1)~~30 250 (79.1)~~	Reference	-	Reference	-	Reference	-
Think so, or confirmed	1180 (21.8)~~1186 (21.6)~~	4243 (78.2)~~4302 (78.4)~~	0.97~~8~~ (0.91 to 1.05)	0.47~~60~~	1.11~~5~~ (1.02~~6~~ to 1.20~~4~~)	~~<~~0.~~0~~01	1.11 (1.02 to 1.20)	0.01
Hardship~~‡~~§	n=8660~~712~~; mean 8.2 (SD 2.7)	n=33 ~~32~~075; mean 7.6 (SD 2.9)	0.936~~5~~ (0.92~~7~~8 to 0.944~~3~~)	<0.001	0.97 (0.96 to 0.98)	<0.001	0.97 (0.96 to 0.98)	<0.001

SD=standard deviation; CI=confidence interval. For continuous variables, odds ratios represent a one unit increase in the explanatory variable, apart from for age, when odds ratios represent a 10 year increase in age.

*Adjusted for survey wave, region, sex, age (raw and quadratic term), dependent child in household, clinically vulnerable to covid-19, household member with a chronic illness, employment status, highest earner works in a manual occupation~~socioeconomic grade~~, index of multiple deprivation, highest educational or professional qualification, ethnicity, and living alone.

†Adjusted for survey wave, region, sex, age (raw and quadratic term), dependent child in household, clinically vulnerable to covid-19, household member with a chronic illness, employment status, socioeconomic grade, index of multiple deprivation, highest educational or professional qualification, ethnicity, and living alone.

‡Not adjusted for employment status as by definition all people who were asked whether they were self-employed were working.

§~~‡~~From 3 (least hardship) to 15 (most hardship).

In sensitivity analyses adjusting for socioeconomic grade, although the difference by index of multiple deprivation was still significant, the association with the second fourth of deprivation no longer reached our threshold for significance (table 3 and supplementary file). No other differences were observed.

When data from 1 June 2020 to 27 January 2021 (wave 19 to 42) were combined, the most commonly reported reasons for not intending to share details of close contacts were not knowing if data would be secure and confidential (14.6%), thinking that the contact tracing system was not accurate and reliable (13.9%), and not knowing what would happen to the data (13.0%; see supplementary file).

Discussion

As in other countries, the test, trace, and isolate system should be a cornerstone of the UK's public health strategy for coping with the covid-19 pandemic.[1] Its success relies on adherence to multiple behaviours.[8] Our data suggest that self-reported rates of full adherence to isolating and testing are low, as are rates of recognition of the main symptoms of covid-19. Rates of intended isolation and testing are higher. The percentage of people who intend to report details of close contacts is also high. However, given that the gap between intended and actual behaviour is a general phenomenon,[28] the percentage of people who do share details of all close contacts after receiving a positive test result is likely to be lower. With such low rates for symptom recognition, testing, and full self-isolation, the effectiveness of the current form of the UK's test, trace, and isolate system is limited.

Comparison with other studies

These low rates of symptom recognition are comparable to those found in other UK research.[9 14 29] Recognising that symptoms might be indicative of covid-19 is a first step in the chain that leads to isolation when required. Greater work to understand why symptom recognition remains low and how to boost it further is important. Further emphasis on specific symptoms might be necessary. In the UK, mass testing of people without symptoms has been introduced in areas with high case prevalence. This removes the need for recognition of symptoms of covid-19, although concerns remain about the effectiveness of mass testing.[30]

Our data suggest that the percentage of people with covid-19 symptoms who request a test has increased over time. Although media attention on testing capacity in the UK was considerable, our data show that increases in capacity were not reflected in the percentage of people with symptoms who requested a test. Despite increasing rates of tests being requested, other research corroborates our finding of a shortfall between national estimates of covid-19 and uptake of antigen tests. Our estimates of the percentage of people requesting a test (eg, 24.5% in late October 2020) is lower than the estimate that can be derived by dividing the number of daily cases identified in the community by NHS Test and Trace[31] by the estimated daily incidence recorded by the ONS (32-52% for late October).[32] Estimates of prevalence of covid-19 in England from the REACT 1 study (a large scale national study investigating the prevalence of covid-19)[33] suggest a greater shortfall. This might be accounted for

by different sample biases, the probable inclusion of people in our sample with an obvious, non-covid-19 explanation for their symptoms, and the probable inclusion of people without symptoms in the NHS Test and Trace data.

When we accounted for duration of isolation, the rates of people adhering to self-isolation were about 20 percentage points higher than those when we did not account for duration of isolation. Few associations reached significance after a Bonferroni correction was applied owing to the inclusion of fewer survey waves and the resulting smaller sample sizes, although some variables showed similar effects to the first analysis. When accounting for duration of isolation, our estimates of adherence to self-isolation were higher than previous data found by our team from May 2020, which suggested that only 25% of people with covid-19 symptoms in their household had not left home in the previous 24 hours.[10] Our rates of self-reported adherence are similar to those referred to in a brief note about a study conducted by the Department of Health and Social Care, which found that 59% of people who were asked to isolate by NHS Test and Trace reported not leaving their home.[34]

In the latest available wave of data collection (25-27 January 2021), 82% of people intended to share details of close contacts if asked to by NHS Test and Trace. According to NHS Test and Trace, 25% of people who test positive for covid-19 do not provide details of any close contacts, suggesting a slight degree of underreporting.[35]

The UK's implementation of test, trace, and isolate differs from that of other countries.[36 37] Although our study focused on behaviour in the UK, the associations found might be generalisable to other countries. For example, the percentages of people with symptoms who self-isolated in our study were comparable to those reported by a similar study in the Netherlands.[38] However, higher rates of testing in the Netherlands means that more people with covid-19 are likely to be identified and therefore the contact tracing system might work more effectively than in the UK. Other factors that might improve the effectiveness of a test, trace, and isolate system include reducing delays between requesting a test and receiving the results.[39]

Implications of the findings

It has been proposed that better financial and practical support might improve rates of adherence to test, trace and isolate behaviours.[40] The importance of support is reflected in the associations we observed in the data, with financial hardship, index of multiple deprivation, lower

socioeconomic status, and having a dependent child in the household showing a pattern of associations with lower adherence to full self-isolation, not requesting a test, and poorer symptom recognition. Evidence from other countries also suggests an association between greater financial hardship and poorer self-isolation.[41] The disproportionate impact of the pandemic on people from lower socioeconomic backgrounds and with carer responsibilities has been well documented.[42 43] Behaviour reflects opportunities and capabilities as well as motivation: people need help to achieve their intentions. While intentions to engage in test, trace, and isolate behaviours are high, a greater focus on financial and practical support is likely to enable more people to translate their intentions into behaviour.[11]

Males and younger people were less likely to engage with testing, self-isolate, and intend to provide details of close contacts. This might reflect poorer health literacy in males, and, among younger people, a greater desire to be active and have contact with peer groups.[44] People who believed they had already experienced covid-19 were less likely to fully self-isolate when symptomatic. Reduced adherence to social distancing measures has also been reported in this group.[14] Other research has found an association between higher education and poorer adherence to UK government guidance.[45] Working in a key sector was also associated with not fully self-isolating. This might be because key workers have a greater financial need to work, feel a greater social pressure to attend work, or are less likely to be able to work from home.[46] Key workers and people from minority ethnic backgrounds were less likely to identify common symptoms of covid-19. Engagement and tailored communications with these groups is likely to improve knowledge of symptoms.

Strengths and limitations of this study

Strengths of this study include the large sample sizes, allowing us to investigate uncommon behaviours and to examine uptake of protective behaviours and knowledge over time. We used quota sampling to ensure that participant characteristics were representative of the UK adult population. Although we cannot be sure that survey respondents are representative of the general population,[47 48] online quota sampling is a pragmatic approach when a large, demographically representative sample needs to be obtained in a short time frame during a crisis.[15 49] Odds ratios should thus be interpreted with some caution. However, issues about representativeness of participants are unlikely to undermine the interpretation of the study. Data were self-reported and so could have been influenced by social desirability and recall gaps and bias. Social

desirability might have become particularly important after September 2020, when adherence to self-isolation became enforceable under law. The anonymity of our surveys should have mitigated this, however. As data are cross sectional, we cannot infer causality.

The nature of an online poll might raise questions as to the level of attention participants pay to their responses. While this is generally no different to any other questionnaire study, the possibility of “professional respondents” is a particular problem in online samples.[50] Assuming such respondents introduce random error into the data, the impact on most items is limited but could become problematic in small subsamples.

Our study was prone to other specific methodological limitations. For symptom identification, we asked participants about the common symptoms of covid-19 and classified responses as symptoms being correctly identified if they selected symptoms promoted to members of the UK public as the “main” symptoms of covid-19 in government guidance.[20] This decision was taken to enable measurement of adherence to policy. However, we recognise that other common symptoms of covid-19 exist (eg, fatigue, headache), which we did not include as being correct. For self-isolation, although we asked participants if they had left home at all since developing covid-19 symptoms, technically it is permissible to leave home under some circumstances, including to attend a medical appointment, to get a test, or when a test result is negative. In our sample, 15.0% of people reported leaving home for a medical need other than covid-19. Therefore, low rates of full self-isolation cannot be explained by permitted outings alone. People receiving a positive covid-19 test result might be more likely to adhere to self-isolation guidance,[51] especially following legal enforcement of self-isolation on 20 September 2020.[52] However, too few people in the sample reported that their test result indicated they had covid-19 to be able to conduct any meaningful analyses. For intention to share details of close contacts, the survey item did not differentiate between household and external contacts.

Although we had a large overall sample size, numbers of participants included in analyses of full self-isolation and requesting a test were smaller, resulting in small cell counts for some analyses. For these variables (region and ethnicity), we used different groupings. For region, we grouped together participants from Scotland, Wales, and Northern Ireland. Test, trace, and isolate systems in the four UK nations are managed locally and problems with the system in one nation might not be observed in other nations. For ethnicity, we grouped together black people, Asian people, and people of mixed ethnicity. This might have obscured differences between ethnic groups.[53]

Conclusions

The spread of covid-19 presents many challenges, not least asymptomatic spread.[54 55] Test, trace, and isolate will never be a complete solution and will be more effective when the reproduction rate of the virus is low.[56] However, it remains an important component of the UK's national response. For the test, trace, and isolate system in the UK to succeed, people must recognise the main symptoms of covid-19 and be able and motivated to self-isolate, request a test, and share details of their close contacts when required. Our results indicate that about half of people know the symptoms of covid-19, and that adherence to each stage of test, trace, and isolate is low but improving slowly. Policies that support people financially and practically, and improving communication about the testing system, will be key to increasing uptake both in the UK and internationally.

Box start

What is already known on this topic

Test, trace, and isolate systems are one of the cornerstones of a national covid-19 recovery strategy

The success of any test, trace, and isolate system relies on people adhering to isolation if they have symptoms, getting a test if symptoms are present, and passing on details of close contacts if infection is confirmed

What this study adds

Self-reported adherence to test, trace, and self-isolate behaviours in the UK population is low; intention to carry out these behaviours is higher

In the UK, identification of covid-19 symptoms is low

Continued improvements to support are likely to be crucial in encouraging more people to adhere to test, trace, and self-isolate behaviours

Box end

Web extra Extra material supplied by authors

Supplementary information: additional tables and figures

Supp-file: smil063382.ww1

We thank Peter Barnes for highlighting an inconsistency in the preprint of this paper.

Contributors: All authors conceived the study and contributed to survey materials. LS completed analyses with guidance from HWWP and GJR. LS and GJR wrote the first draft of the manuscript. All authors contributed to, and approved, the final manuscript. GJR is guarantor. The corresponding author attests that all listed authors meet authorship criteria and that no others meeting the criteria have been omitted.

Funding: LS, RA, and GJR are supported by the National Institute for Health Research Health Protection Research Unit (NIHR HPRU) in Emergency Preparedness and Response, a partnership between Public Health England (PHE), King's College London, and the University of East

Anglia. RA is also supported by the NIHR HPRU in Behavioural Science and Evaluation, a partnership between PHE and the University of Bristol. HWWP receives funding from PHE and NHS England. NTF is part funded by a grant from the UK Ministry of Defence. The views expressed are those of the authors and not necessarily those of the NIHR, PHE, the Department of Health and Social Care, or the Ministry of Defence. Surveys were commissioned and funded by Department of Health and Social Care (DHSC), with the authors providing advice on the question design and selection. DHSC had no role in analysis, decision to publish, or preparation of the manuscript. Preliminary results were made available to DHSC and the UK's Scientific Advisory Group for Emergencies. The funders had no role in considering the study design or in the collection, analysis, interpretation of data, writing of the report, or decision to submit the article for publication.

Competing interests: All authors have completed the ICMJE uniform disclosure form at www.icmje.org/coi_disclosure.pdf and declare: all authors had financial support from NIHR for the submitted work; RA is an employee of Public Health England (PHE); HWWP receives additional salary support from PHE and NHS England; support from the Department of Health and Social Care; no other financial relationships with any organisations that might have an interest in the submitted work in the previous three years; no other relationships or activities that could appear to have influenced the submitted work. NTF is a participant of an independent group advising NHS Digital on the release of patient data. All authors are participants of the UK's Scientific Advisory Group for Emergencies or its subgroups.

Ethical approval: Not required, as this work was conducted as part of service evaluation of the marketing and communications run by the Department of Health and Social Care.

Data sharing: No additional data available.

The authors affirm that the manuscript is an honest, accurate, and transparent account of the study being reported; that no important aspects of the study have been omitted; and that any discrepancies from the study as originally planned have been explained.

Dissemination to participants and related patient and public communities: Dissemination of survey results to participants is not possible owing to the anonymous nature of data collection.

Provenance and peer review: Not commissioned; externally peer reviewed.

<eref>1 UK Government. Our plan to rebuild: The UK Government's COVID-19 recovery strategy. 2020. www.gov.uk/government/publications/our-plan-to-rebuild-the-uk-governments-covid-19-recovery-strategy</eref>

<eref>2 Department of Health and Social Care. NHS Test and Trace: how it works [updated 11 January 2021]. www.gov.uk/guidance/nhs-test-and-trace-how-it-works</eref>

<eref>3 Welsh Government. Test Trace Protect [updated 4 June 2020]. https://gov.wales/test-trace-protect</eref>

<eref>4 Scottish Government. Coronavirus (COVID-19): Test and Protect [updated 14 December 2020]. www.gov.scot/publications/coronavirus-covid-19-test-and-protect/.</eref>

<eref>5 NI Direct Government Services. Coronavirus (COVID-19): testing and contact tracing [cited 19 January 2021]. www.nidirect.gov.uk/articles/coronavirus-covid-19-testing-and-contact-tracing</eref>

<jrn>6 Kucharski AJ, Klepac P, Conlan AJK, et al; CMMID COVID-19 working group. Effectiveness of isolation, testing, contact tracing, and physical distancing on reducing transmission of SARS-CoV-2 in different settings: a mathematical modelling study. *Lancet Infect Dis* 2020;20:1151-60. doi:10.1016/S1473-3099(20)30457-6. PubMed</jrn>

<eref>7 The DELVE Initiative. Test, Trace, Isolate [updated 27 May 2020]. https://rs-delve.github.io/reports/2020/05/27/test-trace-isolate.html</eref>

<jrn>8 Rubin GJ, Smith LE, Melendez-Torres GJ, Yardley L. Improving adherence to 'test, trace and isolate'. *J R Soc Med* 2020;113:335-8. doi:10.1177/0141076820956824. PubMed</jrn>

<eref>9 Allington D, Beaver K, Duffy B, et al. Coronavirus uncertainties: vaccines, symptoms and contested claims [updated 9 August 2020]. www.kcl.ac.uk/policy-institute/assets/coronavirus-uncertainties.pdf</eref>

<jrn>10 Smith LE, Amlôt R, Lambert H, et al. Factors associated with adherence to self-isolation and lockdown measures in the UK: a cross-sectional survey. *Public Health* 2020;187:41-52. doi:10.1016/j.puhe.2020.07.024. PubMed</jrn>

<jrn>11 Atchison C, Bowman LR, Vrinten C, et al. Early perceptions and behavioural responses during the COVID-19 pandemic: a cross-sectional survey of UK adults. *BMJ Open* 2021;11:e043577. doi:10.1136/bmjopen-2020-043577</jrn>

<prpt>12 Pancani L, Marinucci M, Aureli N, Riva P. Forced social isolation and mental health: A study on 1006 Italians under COVID-19 lockdown. PsyArXiv [preprint]. September 25, 2020 [cited 22 November 2020]. https://psyarxiv.com/uacfj/</prpt>

<eref>13 Scientific Advisory Group for Emergencies. SPI-B. SPI-B: Increasing adherence to COVID-19 preventative behaviours among young people. 6 November 2020. https://assets.publishing.service.gov.uk/government/uploads/system/uploads/attachment_data/file/933228/S0829_SPI-B_-_Increasing_adherence_to_Covid-19_preventative_behaviours_among_young_people.pdf</eref>

<jrn>14 Smith LE, Mottershaw AL, Egan M, Waller J, Marteau TM, Rubin GJ. The impact of believing you have had COVID-19 on self-reported behaviour: Cross-sectional survey. *PLoS One* 2020;15:e0240399. doi:10.1371/journal.pone.0240399. PubMed</jrn>

<jrn>15 Rubin GJ, Amlôt R, Page L, Wessely S. Methodological challenges in assessing general population reactions in the immediate aftermath of a terrorist attack. *Int J Methods Psychiatr Res* 2008;17(Suppl 2):S29-35. PubMed doi:10.1002/mpr.270</jrn>

<jrn>16 Kohler U. Possible Uses of Nonprobability Sampling for the Social Sciences. *Surv Methods Insights Field* 2019. doi:10.13094/SMIF-2019-00014.</jrn>

<eref>17 Savanta. Data Collection & Analysis Services [cited 24 November 2020]. https://savanta.com/data-collection-analysis/</eref>

<eref>18 Respondi. Access panel [cited 24 November 2020]. www.respondi.com/EN/access-panel</eref>

<eref>19 Office for National Statistics. Population estimates for the UK, England and Wales, Scotland and Northern Ireland: mid-2018. 2019 [updated 26 June 2019, cited 25 August 2020]. www.ons.gov.uk/peoplepopulationandcommunity/populationandmigration/populationestimates/bulletins/annualmidyearpopulationestimates/mid2018</eref>

<eref>20 Department of Health and Social Care. Statement from the UK Chief Medical Officers on an update to coronavirus symptoms: 18 May 2020 [press release] (18 May 2020). www.gov.uk/government/news/statement-from-the-uk-chief-medical-officers-on-an-update-to-coronavirus-symptoms-18-may-2020</eref>

<eref>21 Ministry of Housing Communities and Local Government. The English Indices of Deprivation 2019 (IoD2019). 26 September 2019. https://assets.publishing.service.gov.uk/government/uploads/system/uploads/attachment_data/file/835115/IoD2019_Statistical_Release.pdf</eref>

<eref>22 NHS. Who's at higher risk of coronavirus [updated 14 August 2020]. www.nhs.uk/conditions/coronavirus-covid-19/people-at-higher-risk/whos-at-higher-risk-from-coronavirus/</eref>

<eref>23 Cabinet Office, Department for Education. Critical workers who can access schools or educational settings [updated 16 June 2020]. www.gov.uk/government/publications/coronavirus-covid-19-maintaining-educational-provision/guidance-for-schools-colleges-and-local-authorities-on-maintaining-educational-provision#critical-workers</eref>

<eref>24 National Readership Survey. Social Grade. www.nrs.co.uk/nrs-print/lifestyle-and-classification-data/social-grade/</eref>

<eref>25 National Health Service. Your coronavirus test result [updated 7 January 2021]. www.nhs.uk/conditions/coronavirus-covid-19/testing-and-tracing/what-your-test-result-means/</eref>

<jrn>26 Rubin GJ, Bakhshi S, Amlôt R, Fear N, Potts HWW, Michie S. The design of a survey questionnaire to measure perceptions and behaviour during an influenza pandemic: the Flu TElephone Survey Template (FluTEST). *Health Services and Delivery Research* 2014;2. doi:10.3310/hsdr02410. PubMed</jrn>

<eref>27 GOV.UK. Testing in United Kingdom [updated 8 February 2021]. https://coronavirus.data.gov.uk/details/testing</eref>

<jrn>28 Sheeran P, Webb TL. The intention-behaviour gap. *Soc Personal Psychol Compass* 2016;10:503-18. doi:10.1111/spc3.12265.</jrn>

<eref>29 YouGov. What are the symptoms of COVID-19? Only 59% of Britons know all three 2020 [updated 25 June 2020]. https://yougov.co.uk/topics/health/articles-reports/2020/06/25/what-are-symptoms-covid-19-only-59-britons-know-al</eref>

<jrn>30 Wise J. Covid-19: Concerns persist about purpose, ethics, and effect of rapid testing in Liverpool. *BMJ* 2020;371:m4690. doi:10.1136/bmj.m4690. PubMed</jrn>

<eref>31 Department of Health and Social Care. NHS Test and Trace. Weekly statistics for NHS Test and Trace (England) and coronavirus testing (UK): 22 October to 28 October. 5 November 2020. www.gov.uk/government/publications/nhs-test-and-trace-england-and-coronavirus-testing-uk-statistics-22-october-to-28-october/weekly-statistics-for-nhs-test-and-trace-england-and-coronavirus-testing-uk-22-october-to-28-october</eref>

<eref>32 Office for National Statistics. Coronavirus (COVID-19) Infection Survey,UK: 6 November 2020 [updated 6 November 2020]. www.ons.gov.uk/peoplepopulationandcommunity/healthandsocialcare/conditionsanddiseases/bulletins/coronaviruscovid19infectionsurveypilot/6november2020</eref>

<prpt>33 Riley S, Ainslie KEC, Eales O, et al. REACT-1 round 6 updated report: high prevalence of SARS-CoV-2 swab positivity with reduced rate of growth in England at the start of November 2020. MedRxiv [preprint]. November 20, 2020 [cited 21 January 2021] www.medrxiv.org/content/medrxiv/early/2020/11/20/2020.11.18.20233932.full.pdf</prpt>

<eref>34 Environmental Modelling Group (EMG), Scientific Pandemic Insights Group on Behaviours (SPI-B), Scientific Pandemic Influenza Group on Modelling (SPI-M). EMG/SPI-B/SPI-M: Reducing within- and between-household transmission in light of new variant SARS-CoV-2, 15 January 2021. 15 January 2021. https://assets.publishing.service.gov.uk/government/uploads/system/uploads/attachment_data/file/952799/s1020-Reducing-within-between-household-transmission.pdf</eref>

<eref>35 Department of Health and Social Care. Weekly statistics for NHS Test and Trace (England) and coronavirus testing (UK): 21 January to 27 January 2021. 4 February 2021. www.gov.uk/government/publications/nhs-test-and-trace-england-statistics-21-january-to-27-january-2021/weekly-statistics-for-nhs-test-and-trace-england-21-january-to-27-january-2021#tracing-england</eref>

<jrn>36 Lewis D. Where COVID contact-tracing went wrong. *Nature* 2020;588:384-88.</jrn>

<eref>37 Royal Statistical Society. Royal Statistical Society (RSS) COVID-19 Task Force Statement on how efficient statistical methods can glean greater intelligence from Test, Trace and Isolate (TTI), officially known as Test & Trace 2020 [updated 23 July 2020]. https://rss.org.uk/RSS/media/File-library/Policy/RSS-COVID-19-Task-Force-Statement-on-TTI-final.pdf</eref>

<eref>38 Rijksinstituut voor Volksgezondheid en Milieu. Research on behavioural rules and well-being: round 9 [updated 19 January 2021]. www.rivm.nl/en/novel-coronavirus-covid-19/research/behaviour/-behavioural-rules-and-well-being-round-9</eref>

<jrn>39 Kretzschmar ME, Rozhnova G, Bootsma MCJ, van Boven M, van de Wijgert JHHM, Bonten MJM. Impact of delays on effectiveness of contact tracing strategies for COVID-19: a modelling study. *Lancet Public Health* 2020;5:e452-9. doi:10.1016/S2468-2667(20)30157-2. PubMed</jrn>

<eref>40 Scientific Advisory Group for Emergencies. SPI-B. The impact of financial and other targeted support on rates of self-isolation or quarantine [SPI-B: 16 September 2020]. 16 September 2020. https://assets.publishing.service.gov.uk/government/uploads/system/uploads/attachment_data/file/925133/S0759_SPI-B__The_impact_of_financial_and_other_targeted_support_on_rates_of_self-isolation_or_quarantine_.pdf</eref>

<jrn>41 Bodas M, Peleg K. Self-Isolation Compliance In The COVID-19 Era Influenced By Compensation: Findings From A Recent Survey In Israel. *Health Aff (Millwood)* 2020;39:936-41. doi:10.1377/hlthaff.2020.00382. PubMed</jrn>

<jrn>42 Blundell R, Costa Dias M, Joyce R, Xu X. COVID-19 and Inequalities. *Fisc Stud* 2020. Published online 14 July. doi:10.1111/1475-5890.12232. PubMed</jrn>

<eref>43 Andrew A, Cattan S, Costa Dias M, et al. How are mothers and fathers balancing work and family under lockdown? The Institute for Fiscal Studies. May 2020. www.ifs.org.uk/publications/14860</eref>

<prpt>44 Seng JJB, Yeam CT, Huang WC, Tan NC, Low LL. Pandemic related Health literacy - A Systematic Review of literature in COVID-19, SARS and MERS pandemics. medRxiv [prepint]. May 11, 2020 [cited 20 August 2020] www.medrxiv.org/content/10.1101/2020.05.07.20094227v1</prpt>

<eref>45 Fancourt D, Bu F, Mak HW, Steptoe A. Covid-19 Social Study; Results Release 25. 19 November 2020. https://b6bdcb03-332c-4ff9-8b9d-28f9c957493a.filesusr.com/ugd/3d9db5_10010a26414a4f6eafeea8b24fd89936.pdf</eref>

<eref>46 Office for National Statistics. Coronavirus and key workers in the UK 2020 [updated 15 May 2020]. www.ons.gov.uk/employmentandlabourmarket/peopleinwork/earningsandworkinghours/articles/coronavirusandkeyworkersintheuk/2020-05-15</eref>

<eref>47 Office for National Statistics. Internet users, UK: 2019. www.ons.gov.uk/businessindustryandtrade/itandinternetindustry/bulletins/internetusers/2019</eref>

<jrn>48 Wright KB. Researching Internet-Based Populations Advantages and Disadvantages of Online Survey Research, Online Questionnaire Authoring Software Packages, and Web Survey Services. *J Comput Mediat Commun* 2005;10. doi:10.1111/j.1083-6101.2005.tb00259.x.</jrn>

<jrn>49 Rubin GJ, Amlôt R, Page L, Wessely S. Public perceptions, anxiety, and behaviour change in relation to the swine flu outbreak: cross sectional telephone survey. *BMJ* 2009;339:b2651. doi:10.1136/bmj.b2651. PubMed</jrn>

<jrn>50 Matthijsse SM, de Leeuw ED, Hox JJ. Internet Panels, Professional Respondents, and Data Quality. *Methodology* 2015;11:81-8. doi:10.1027/1614-2241/a000094.</jrn>

<eref>51 Rijksinstituut voor Volksgezondheid en Milieu. Coronavirus measures: public support remains high, despite concerns [updated 22 October 2020]. www.rivm.nl/en/news/coronavirus-measures-public-support-remains-high-despite-concerns</eref>

<eref>52 Prime Minister’s Office. New package to support and enforce self-isolation [press release] (20 September 2020). www.gov.uk/government/news/new-package-to-support-and-enforce-self-isolation</eref>

<eref>53 Public Health England. Beyond the data: Understanding the impact of COVID-19 on BAME groups. June 2020. https://assets.publishing.service.gov.uk/government/uploads/system/uploads/attachment_data/file/892376/COVID_stakeholder_engagement_synthesis_beyond_the_data.pdf</eref>

<jrn>54 Johansson MA, Quandelacy TM, Kada S, et al. SARS-CoV-2 Transmission From People Without COVID-19 Symptoms. *JAMA Netw Open* 2021;4:e2035057. doi:10.1001/jamanetworkopen.2020.35057. PubMed</jrn>

<prpt>55 Ferretti L, Ledda A, Wymant C, Zhao L, Ledda V. Abeler- Dorner L, et al. The timing of COVID-19 transmission. MedRxiv[prepint]. September 16, 2020 [cited 19 January 2021] www.medrxiv.org/content/10.1101/2020.09.04.20188516v2.full.pdf</prpt>

<jrn>56 Hellewell J, Abbott S, Gimma A, et al; Centre for the Mathematical Modelling of Infectious Diseases COVID-19 Working Group. Feasibility of controlling COVID-19 outbreaks by isolation of cases and contacts. *Lancet Glob Health* 2020;8:e488-96. doi:10.1016/S2214-109X(20)30074-7. PubMed</jrn>

<eref>57 Department of Health and Social Care. Everyone in the United Kingdom with symptoms now eligible for coronavirus tests [press release] (18 May 2020). www.gov.uk/government/news/everyone-in-the-united-kingdom-with-symptoms-now-eligible-for-coronavirus-tests</eref>
