# Supplementary material for: Adherence to the test, trace, and isolate system in the UK: results from 37 nationally representative surveys
Source: BMJ. 2021 Mar 31;372:n608. doi: 10.1136/bmj.n608 (PMC8010268; doi:10.1136/bmj.n608)
Supplement: Supplementary file 1 [file smil063382.ww1.pdf]

Supplementary materials. Excluding people who tested negative for covid-19 from self-isolation analyses

We used data from different questions to identify participants who tested negative for covid-19. In data collected between 1 June and 5 August 2020 (waves 19 to 26), participants who reported they had requested a test after developing covid-19 symptoms were asked to indicate their test result or if they were still waiting for results. In data collected from 7 December 2020 (wave 36 onwards), all participants were asked if they had been tested for covid-19 in the last seven days, and if so, what the results of their test were. In data collected from 11 January 2021 (wave 41 onwards), having received a negative test was included in the list of reasons why you had left home since developing symptoms (only shown to participants who reported having covid-19 symptoms and who reported having requested a test). In all waves, participants with covid-19 symptoms could specify "other" reasons for having left home.

Supplementary materials. Endorsement of common symptoms of covid-19

Can you tell us what you think the most common symptoms of coronavirus are? Please select up to 5	26 May 2020 to 27 January 2021, n†	%	25 to 27 January 2021, n‡	%
Cough	37,336	73.8	1516	75.5
High temperature / fever	36,560	72.2	1383	68.9
Loss of sense of smell	33,280	65.7	1424	71.0
Loss of taste	32,211	63.6	1422	70.9
Shortness of breath / difficulty breathing	27,064	53.5	1142	56.9
Flu-like symptoms	14,351	28.4	483	24.1
Sore throat	8,126	16.1	275	13.7
Feeling tired or having low energy	7,986	15.8	325	16.2
Headaches	5,028	9.9	214	10.7
Loss of appetite	4,086	8.1	164	8.2
Aches and pains	2,855	5.6	123	6.1
Chest pain	2,516	5.0	86	4.3
Sneezing	2,382	4.7	53	2.6
Chills / shivering	2,225	4.4	98	4.9
Nausea / feeling sick	1,550	3.1	73	3.6
Diarrhoea	1,482	2.9	51	2.5
Runny nose	1,449	2.9	45	2.2
Pain in your arms, legs or joints	1,126	2.2	42	2.1
Blocked nose	870	1.7	39	1.9
Dizziness	879	1.7	38	1.9
Vomiting	787	1.6	36	1.8
Feeling your heart pound or race	598	1.2	23	1.1
Stomach ache	538	1.1	21	1.0
Trouble sleeping	516	1.0	16	0.8
Back pain	511	1.0	21	1.0
Fainting spells	440	0.9	23	1.1
Don’t know (single code)	1,189	2.3	33	1.6

† Baseline n=50,617.

‡ Baseline n=2,007.

Supplementary materials. Lowess plots showing associations between outcomes and age.

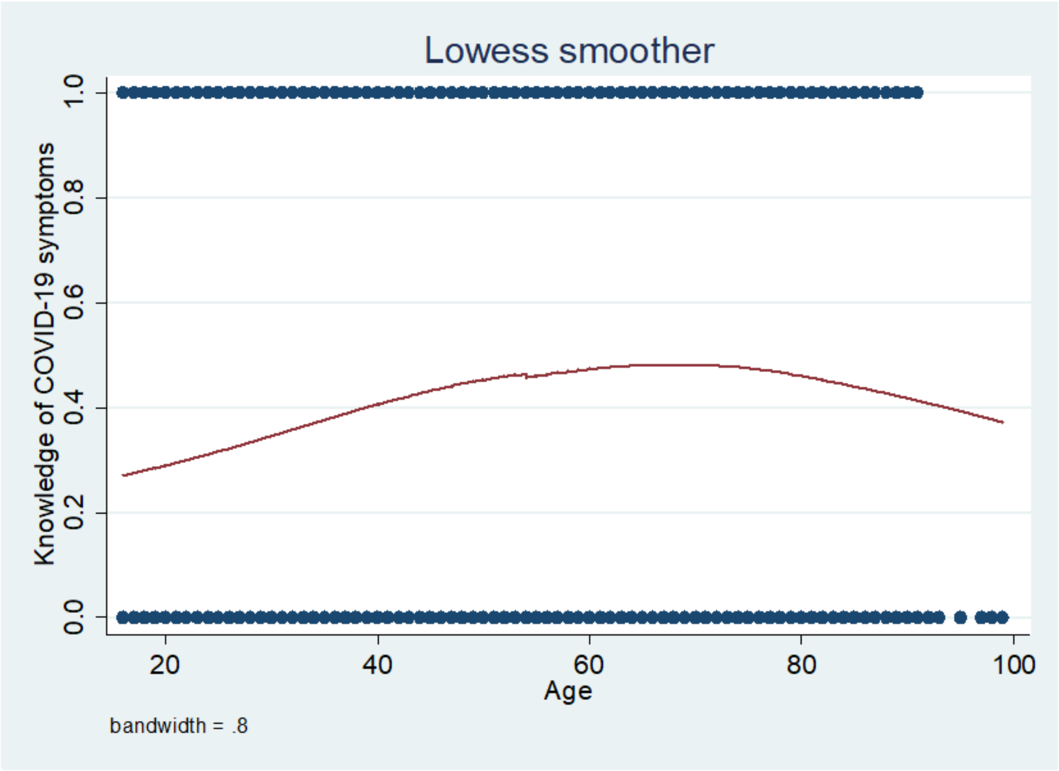

Figure 1. Symptom identification and age.

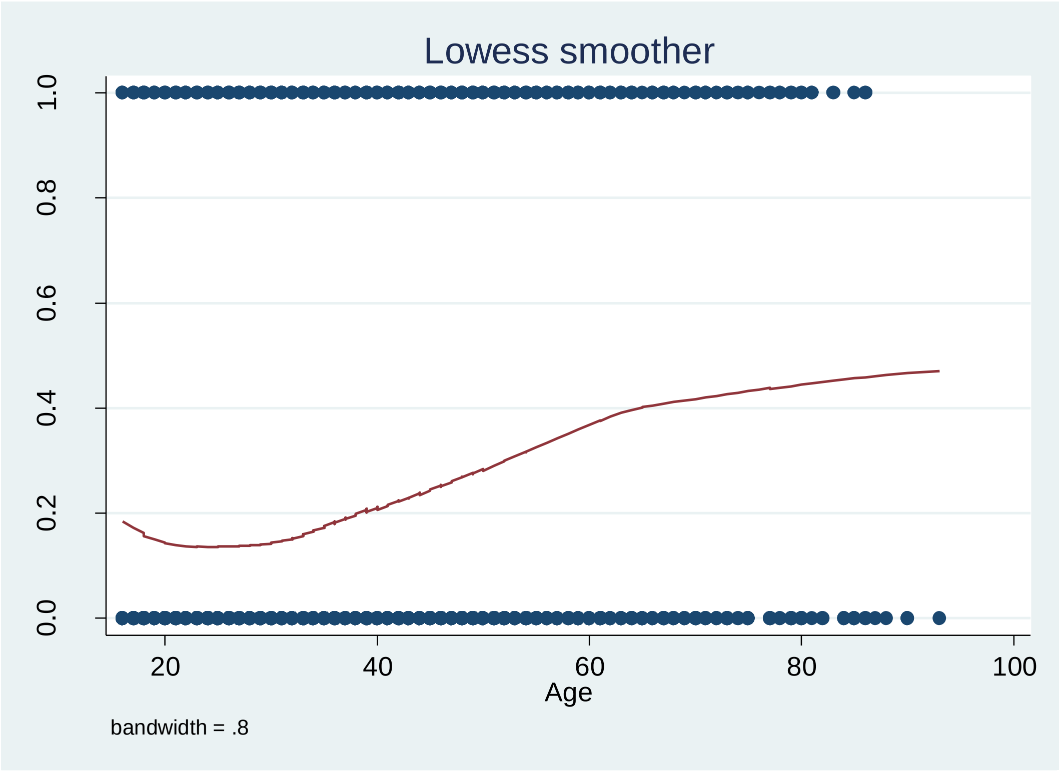

Figure 2. Full self-isolation and age.

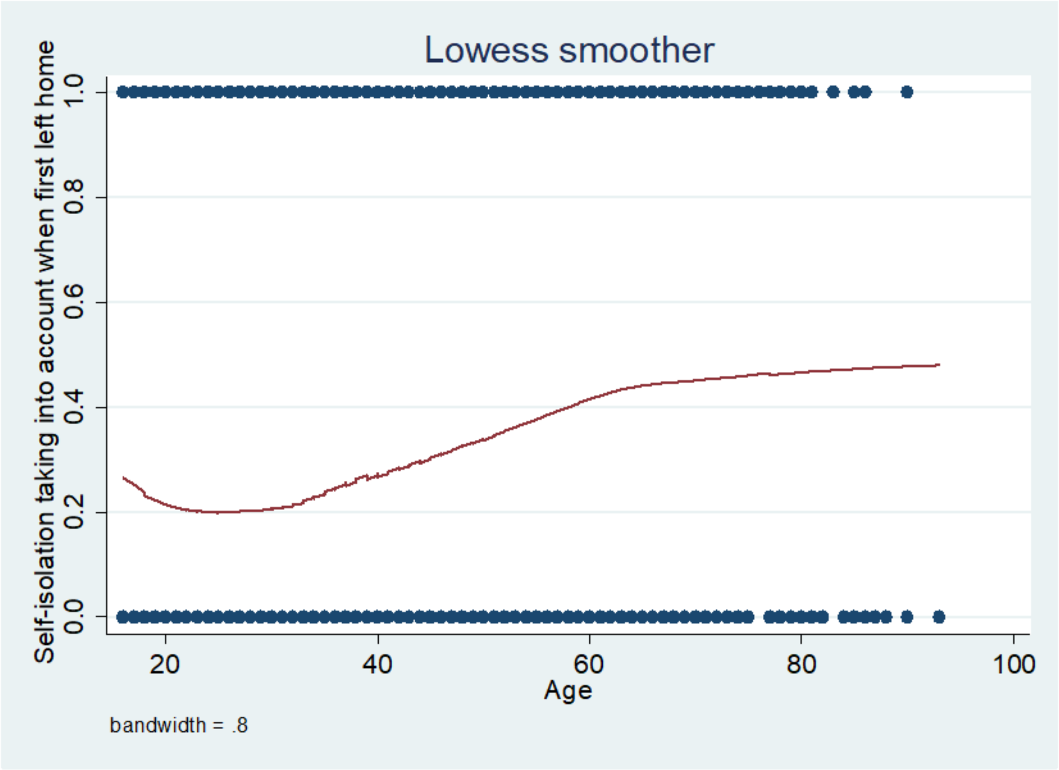

Figure 3. Full self-isolation accounting for duration of isolation and age.

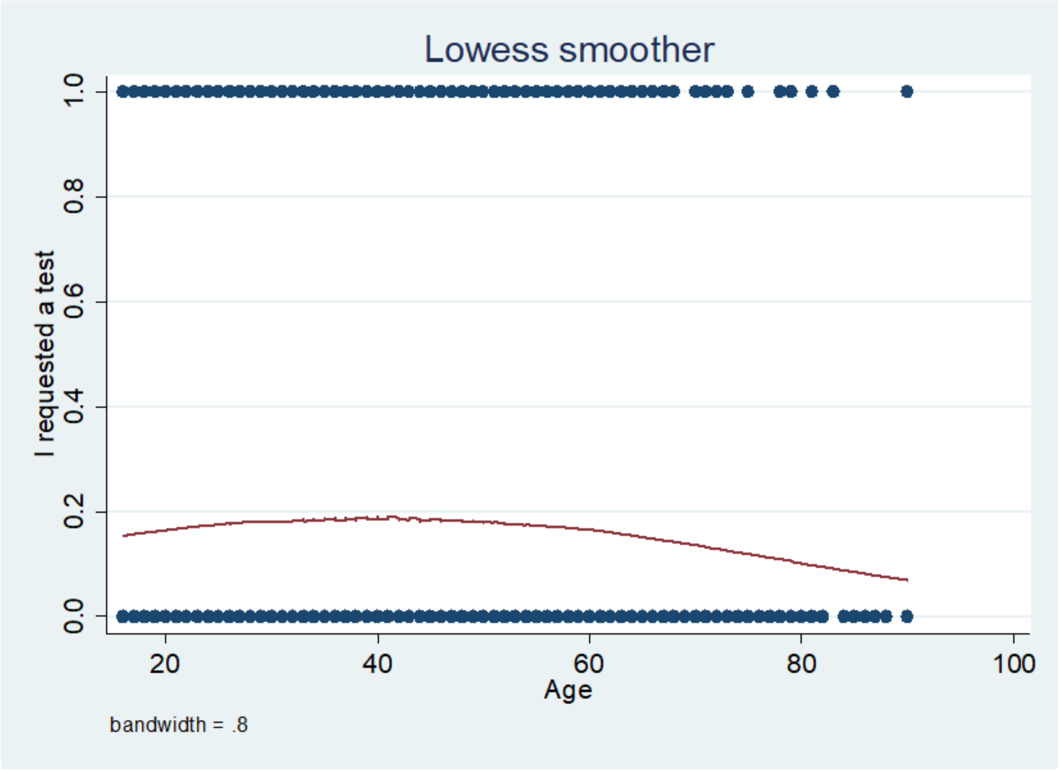

Figure 4. Requesting a test after developing symptoms of covid-19 and age.

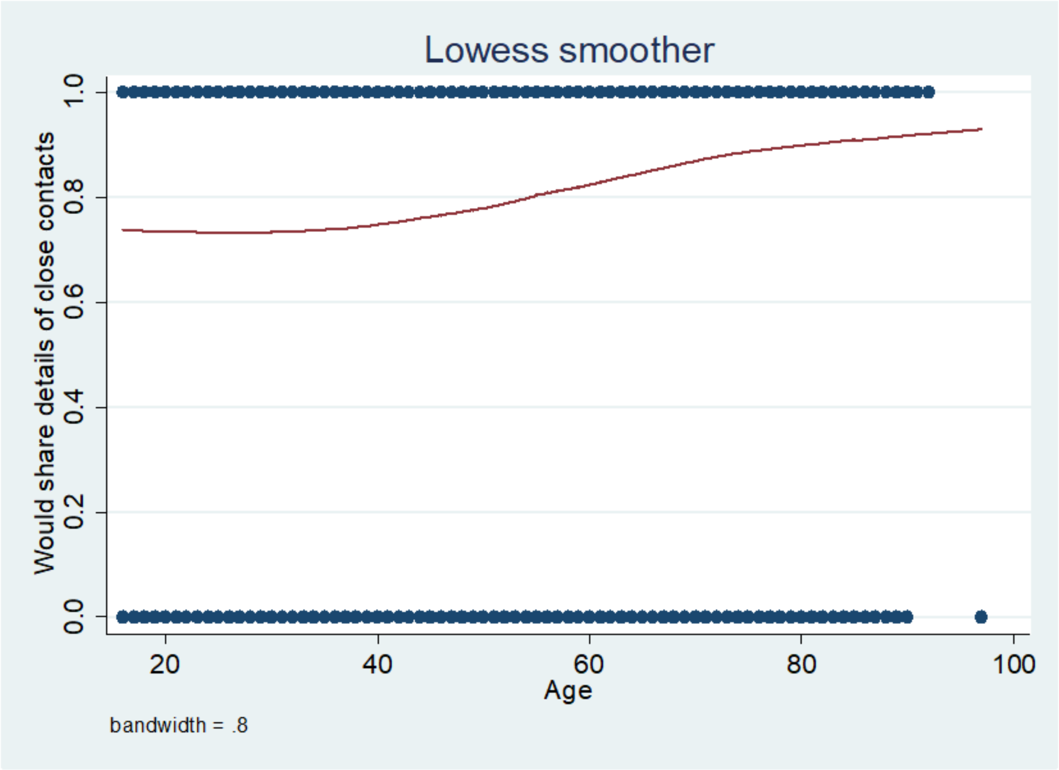

Figure 5. Intention to share details of your close contacts with the NHS contact tracing service and age.

Supplementary materials. Associations between outcomes and survey wave and region.

Table 1. Symptom identification and associations with survey wave and region. Bolding indicates findings significant at a $p<0.001$.

Participant characteristics	Level	Did not identify cough, high temperature / fever, and loss of sense of smell or taste n=23,638	Identified cough, high temperature / fever, and loss of sense of smell or taste n=24,889	Odds ratio (95% CI) for correctly identifying symptoms	p-value	Adjusted odds ratio (95% CI) for correctly identifying symptoms†	p-value
Survey wave	26 to 27 May 2020 (wave 18)	1,047 (52.4)	951 (47.6)	Reference	-	Reference	-
	1 to 3 June 2020 (wave 19)	980 (49.1)	1,014 (50.9)	1.17 (1.04 to 1.31)	0.008	1.05 (0.93 to 1.19)	0.39
	8 to 10 June 2020 (wave 20)	981 (49.1)	1,015 (50.9)	1.13 (1.01 to 1.27)	0.03	1.03 (0.91 to 1.17)	0.64
	15 to 17 June 2020 (wave 21)	1,020 (51.1)	978 (48.9)	1.02 (0.91 to 1.14)	0.77	0.94 (0.83 to 1.06)	0.33
	22 to 24 June 2020 (wave 22)	1,025 (51.2)	978 (48.8)	1.07 (0.95 to 1.20)	0.28	0.97 (0.86 to 1.10)	0.65
	29 June to 1 July 2020 (wave 23)	1,017 (50.9)	980 (49.1)	1.05 (0.93 to 1.17)	0.45	0.93 (0.82 to 1.05)	0.25
	6 to 8 July 2020 (wave 24)	1,068 (53.3)	935 (46.7)	0.94 (0.84 to 1.06)	0.33	0.86 (0.76 to 0.97)	0.01
	20 to 22 July 2020 (wave 25)	1,043 (51.8)	971 (48.2)	1.04 (0.93 to 1.17)	0.48	0.98 (0.86 to 1.11)	0.72
	3 to 5 August 2020 (wave 26)	1,008 (50.4)	991 (49.6)	1.03 (0.92 to 1.16)	0.60	0.97 (0.85 to 1.09)	0.59
	1 to 2 September 2020 (wave 27)	1,062 (52.6)	956 (47.4)	0.97 (0.86 to 1.10)	0.67	0.88 (0.78 to 1.00)	0.06
	14 to 16 September 2020 (wave 28)	1,002 (49.8)	1,011 (50.2)	1.07 (0.95 to 1.21)	0.26	0.98 (0.86 to 1.11)	0.77
	28 to 30 September 2020 (wave 29)	904 (44.4)	1,132 (55.6)	**1.31 (1.16 to 1.47)**	**<0.001**	1.20 (1.06 to 1.36)	0.004
	12 to 14 October 2020 (wave 30)	936 (45.2)	1,136 (54.8)	**1.27 (1.13 to 1.43)**	**<0.001**	1.13 (1.00 to 1.28)	0.06
	26 to 28 October 2020 (wave 31)*	902 (44.2)	1,141 (55.8)	**1.33 (1.18 to 1.50)**	**<0.001**	-	-
	9 to 11 November 2020 (wave 32)	927 (45.7)	1,102 (54.3)	**1.25 (1.11 to 1.41)**	**<0.001**	1.13 (0.99 to 1.28)	0.06
	16 to 18 November 2020 (wave 33)	977 (47.5)	1,082 (52.5)	1.19 (1.06 to 1.34)	0.004	1.08 (0.95 to 1.23)	0.22
	23 to 25 November 2020 (wave 34)	967 (47.8)	1,058 (52.2)	1.14 (1.01 to 1.28)	0.03	1.02 (0.90 to 1.16)	0.77
	30 November to 2 December 2020 (wave 35)	983 (48.0)	1,067 (52.0)	1.15 (1.03 to 1.30)	0.02	1.10 (0.97 to 1.24)	0.15
	7 to 9 December 2020 (wave 36)	966 (45.9)	1,139 (54.1)	**1.23 (1.10 to 1.38)**	**<0.001**	1.10 (0.97 to 1.25)	0.13
	14 to 16 December 2020 (wave 37)	947 (46.9)	1,074 (53.1)	1.18 (1.05 to 1.33)	0.005	1.07 (0.94 to 1.21)	0.30
	21 to 23 December 2020 (wave 38)	951 (47.2)	1,064 (52.8)	1.17 (1.04 to 1.31)	0.01	1.06 (0.93 to 1.20)	0.40
	28 to 30 December 2020 (wave 39)	950 (46.8)	1,078 (53.2)	1.20 (1.07 to 1.36)	0.002	1.06 (0.93 to 1.20)	0.38
	4 to 6 January 2021 (wave 40)	934 (45.7)	1,108 (54.3)	1.23 (1.09 to 1.39)	0.001	1.16 (1.03 to 1.32)	0.02
	11 to 13 January 2021 (wave 41)	955 (47.6)	1,050 (52.4)	1.17 (1.04 to 1.32)	0.008	1.06 (0.94 to 1.20)	0.36
	25 to 27 January 2021 (wave 42)	988 (49.2)	1,019 (50.8)	1.08 (0.96 to 1.22)	0.21	0.98 (0.86 to 1.11)	0.75
Region‡	East Midlands	1,785 (46.6)	2,044 (53.4)	Reference	-	Reference	-
	East of England	2,109 (45.1)	2,571 (54.9)	1.06 (0.97 to 1.17)	0.21	1.00 (0.91 to 1.11)	0.95
	London	3,484 (58.3)	2,495 (41.7)	**0.61 (0.56 to 0.67)**	**<0.001**	**0.76 (0.69 to 0.83)**	**<0.001**
	North East	1,035 (47.2)	1,156 (52.8)	0.97 (0.87 to 1.09)	0.65	0.97 (0.86 to 1.09)	0.62
	North West	2,773 (50.7)	2,693 (49.3)	**0.85 (0.77 to 0.93)**	**<0.001**	0.86 (0.78 to 0.94)	0.002
	Northern Ireland	396 (45.4)	477 (54.6)	1.08 (0.92 to 1.27)	0.37	1.06 (0.89 to 1.25)	0.51
	Scotland	1,770 (47)	1,999 (53)	0.98 (0.89 to 1.08)	0.71	0.95 (0.86 to 1.05)	0.34
	South East	2,944 (45.7)	3,498 (54.3)	1.04 (0.95 to 1.13)	0.41	0.98 (0.90 to 1.08)	0.70
	South West	1,936 (46.3)	2,241 (53.7)	1.00 (0.91 to 1.10)	0.98	0.95 (0.86 to 1.05)	0.30
	Wales	1,085 (45.6)	1,294 (54.4)	1.02 (0.91 to 1.14)	0.69	0.95 (0.84 to 1.06)	0.35
	West Midlands	2,164 (50.0)	2,161 (50.0)	0.87 (0.79 to 0.96)	0.004	0.94 (0.85 to 1.04)	0.22

	Yorkshire and the Humber	2,157 (48.8)	2,260 (51.2)	0.90 (0.82 to 1.00)	0.04	0.89 (0.81 to 0.99)	0.02

* Wave 31 data were excluded from analyses, therefore these data are not included in the totals in the column headers.

† Adjusting for survey wave, region, sex, age (raw and quadratic term), dependent child in the household, being clinically vulnerable to covid-19, having a household member with a chronic illness, employment status, socioeconomic grade, index of multiple deprivation, highest educational or professional qualification, ethnicity, and living alone.

‡ For most GEE analyses, we used an exchangeable correlation structure. This failed to converge for the univariable analysis for region, so we used an unstructured correlation structure.

Table 2. Full self-isolation after developing symptoms of covid-19 and associations with survey wave and region. Bolding indicates findings significant at a $p<0.001$.

Participant characteristics	Level	Did not fully self-isolate n=2,769	Fully self-isolated n=688	Odds ratio (95% CI) for fully self-isolating	p-value	Adjusted odds ratio (95% CI) for fully self-isolating †	p-value
Survey wave	14 to 15 April 2020 (wave 12)	107 (80.5)	26 (19.5)	Reference	-	Reference	-
	20 to 22 April 2020 (wave 13)	119 (83.8)	23 (16.2)	0.78 (0.44 to 1.41)	0.41	0.99 (0.53 to 1.86)	0.97
	27 to 29 April (wave 14)	88 (80.0)	22 (20.0)	0.98 (0.53 to 1.81)	0.96	1.30 (0.66 to 2.55)	0.45
	4 to 6 May 2020 (wave 15)	93 (80.9)	22 (19.1)	0.95 (0.52 to 1.74)	0.87	0.95 (0.49 to 1.84)	0.87
	11 to 13 May (wave 16)	104 (84.6)	19 (15.4)	0.82 (0.46 to 1.47)	0.51	0.94 (0.51 to 1.76)	0.86
	18 to 20 May 2020 (wave 17)	84 (73.7)	30 (26.3)	1.42 (0.81 to 2.48)	0.22	1.26 (0.67 to 2.38)	0.47
	26 to 27 May 2020 (wave 18)	139 (79.0)	37 (21.0)	1.08 (0.64 to 1.82)	0.78	0.99 (0.55 to 1.79)	0.97
	1 to 3 June 2020 (wave 19)	98 (74.2)	34 (25.8)	1.34 (0.78 to 2.32)	0.29	1.38 (0.75 to 2.52)	0.30
	8 to 10 June 2020 (wave 20)	106 (80.3)	26 (19.7)	0.94 (0.53 to 1.67)	0.84	0.70 (0.37 to 1.34)	0.29
	15 to 17 June 2020 (wave 21)	102 (86.4)	16 (13.6)	0.63 (0.34 to 1.18)	0.15	0.62 (0.30 to 1.27)	0.20
	22 to 24 June 2020 (wave 22)	112 (88.2)	15 (11.8)	0.57 (0.30 to 1.07)	0.08	0.52 (0.26 to 1.02)	0.06
	29 June to 1 July 2020 (wave 23)	91 (80.5)	22 (19.5)	0.99 (0.55 to 1.78)	0.96	1.01 (0.53 to 1.90)	0.99
	6 to 8 July 2020 (wave 24)	94 (83.9)	18 (16.1)	0.85 (0.46 to 1.56)	0.60	1.00 (0.51 to 1.94)	0.99
	20 to 22 July 2020 (wave 25)	107 (85.6)	18 (14.4)	0.68 (0.37 to 1.25)	0.21	0.66 (0.34 to 1.28)	0.21
	3 to 5 August 2020 (wave 26)	87 (84.5)	16 (15.5)	0.67 (0.34 to 1.34)	0.26	0.68 (0.31 to 1.47)	0.32
	1 to 2 September 2020 (wave 27)	109 (81.3)	25 (18.7)	0.91 (0.50 to 1.63)	0.74	0.78 (0.40 to 1.52)	0.46
	14 to 16 September 2020 (wave 28)	110 (86.6)	17 (13.4)	0.61 (0.32 to 1.17)	0.14	0.44 (0.22 to 0.88)	0.02
	28 to 30 September 2020 (wave 29)	98 (87.5)	14 (12.5)	0.56 (0.29 to 1.10)	0.09	0.51 (0.24 to 1.07)	0.08
	12 to 14 October 2020 (wave 30)	77 (72.6)	29 (27.4)	1.35 (0.75 to 2.43)	0.32	1.03 (0.54 to 1.97)	0.93
	26 to 28 October 2020 (wave 31)*	78 (70.9)	32 (29.1)	1.61 (0.91 to 2.85)	0.10	-	-
	9 to 11 November 2020 (wave 32)	104 (81.9)	23 (18.1)	0.86 (0.48 to 1.57)	0.63	0.70 (0.37 to 1.35)	0.29
	16 to 18 November 2020 (wave 33)	99 (77.3)	29 (22.7)	1.14 (0.65 to 2.02)	0.65	1.04 (0.54 to 1.99)	0.92
	23 to 25 November 2020 (wave 34)	88 (79.3)	23 (20.7)	1.03 (0.57 to 1.87)	0.92	0.75 (0.38 to 1.46)	0.40
	30 November to 2 December 2020 (wave 35)	103 (88.0)	14 (12.0)	0.56 (0.29 to 1.10)	0.09	0.39 (0.18 to 0.85)	0.02
	7 to 9 December 2020 (wave 36)	58 (65.9)	30 (34.1)	1.97 (1.10 to 3.53)	0.02	1.59 (0.81 to 3.10)	0.17
	14 to 16 December 2020 (wave 37)	71 (79.8)	18 (20.2)	1.06 (0.56 to 2.02)	0.85	0.79 (0.39 to 1.58)	0.50
	21 to 23 December 2020 (wave 38)	69 (77.5)	20 (22.5)	1.09 (0.57 to 2.09)	0.79	1.06 (0.52 to 2.17)	0.86
	28 to 30 December 2020 (wave 39)	66 (77.6)	19 (22.4)	1.11 (0.57 to 2.15)	0.76	0.82 (0.39 to 1.71)	0.60
	4 to 6 January 2021 (wave 40)	72 (67.9)	34 (32.1)	1.83 (1.04 to 3.23)	0.04	1.76 (0.93 to 3.34)	0.08
	11 to 13 January 2021 (wave 41)	57 (71.3)	23 (28.8)	1.56 (0.83 to 2.93)	0.17	1.14 (0.56 to 2.29)	0.72
	25 to 27 January 2021 (wave 42)	57 (68.7)	26 (31.3)	1.65 (0.89 to 3.09)	0.11	1.70 (0.85 to 3.40)	0.13
Region	East Midlands	187 (79.6)	48 (20.4)	Reference	-	Reference	-

	East of England	219 (80.2)	54 (19.8)	1.02 (0.66 to 1.60)	0.92	1.09 (0.66 to 1.81)	0.73
	London	621 (87.1)	92 (12.9)	0.60 (0.40 to 0.89)	0.01	0.93 (0.59 to 1.46)	0.74
	North East	109 (74.7)	37 (25.3)	1.45 (0.88 to 2.38)	0.15	1.48 (0.83 to 2.65)	0.19
	North West	335 (82.1)	73 (17.9)	0.89 (0.59 to 1.34)	0.57	1.04 (0.66 to 1.64)	0.86
	Northern Ireland	37 (90.2)	4 (9.8)	0.46 (0.16 to 1.37)	0.16	0.32 (0.09 to 1.17)	0.08
	Scotland	169 (74.4)	58 (25.6)	1.41 (0.90 to 2.20)	0.13	1.33 (0.79 to 2.22)	0.28
	South East	272 (73.3)	99 (26.7)	1.49 (1.00 to 2.21)	0.05	1.58 (1.00 to 2.51)	0.05
	South West	179 (76.8)	54 (23.2)	1.18 (0.75 to 1.85)	0.47	1.33 (0.80 to 2.23)	0.27
	Wales	96 (72.2)	37 (27.8)	1.64 (0.99 to 2.72)	0.06	1.87 (1.06 to 3.3)	0.03
	West Midlands	291 (80.8)	69 (19.2)	0.97 (0.64 to 1.47)	0.88	1.30 (0.81 to 2.09)	0.28
	Yorkshire and the Humber	254 (80.1)	63 (19.9)	0.94 (0.61 to 1.45)	0.78	0.99 (0.61 to 1.62)	0.98

* Wave 31 data were excluded from analyses, therefore these data are not included in the totals in the column headers.

† Adjusting for survey wave, region, sex, age (raw and quadratic term), dependent child in the household, being clinically vulnerable to covid-19, having a household member with a chronic illness, employment status, socioeconomic grade, index of multiple deprivation, highest educational or professional qualification, ethnicity, and living alone.

Table 3. Full self-isolation after developing symptoms of covid-19 accounting for duration of isolation and associations with survey wave and region. Bolding indicates findings significant at a $p<0.001$.

Participant characteristics	Level	Did not fully self-isolate n=648	Fully self-isolated n=455	Odds ratio (95% CI) for fully self-isolating	p-value	Adjusted odds ratio (95% CI) for fully self-isolating †	p-value
Survey wave	26 to 28 October 2020 (wave 31)*	50 (45.5)	60 (54.5)	Reference	-	-	-
	9 to 11 November 2020 (wave 32)	79 (62.2)	48 (37.8)	0.50 (0.30 to 0.84)	0.09	Reference	-
	16 to 18 November 2020 (wave 33)	71 (55.5)	57 (44.5)	0.67 (0.41 to 1.11)	0.12	1.31 (0.77 to 2.22)	0.32
	23 to 25 November 2020 (wave 34)	65 (58.6)	46 (41.4)	0.59 (0.35 to 1.00)	0.05	1.14 (0.66 to 2.00)	0.64
	30 November to 2 December 2020 (wave 35)	74 (63.2)	43 (36.8)	0.49 (0.29 to 0.83)	0.008	0.89 (0.50 to 1.57)	0.69
	7 to 9 December 2020 (wave 36)	45 (51.1)	43 (48.9)	0.80 (0.47 to 1.36)	0.40	1.47 (0.82 to 2.63)	0.20
	14 to 16 December 2020 (wave 37)	59 (66.3)	30 (33.7)	0.46 (0.25 to 0.83)	0.01	0.75 (0.42 to 1.35)	0.34
	21 to 23 December 2020 (wave 38)	60 (67.4)	29 (32.6)	0.41 (0.23 to 0.72)	0.002	0.68 (0.37 to 1.24)	0.21
	28 to 30 December 2020 (wave 39)	50 (58.8)	35 (41.2)	0.58 (0.33 to 1.02)	0.06	1.02 (0.56 to 1.87)	0.95
	4 to 6 January 2021 (wave 40)	56 (52.8)	50 (47.2)	0.73 (0.43 to 1.25)	0.25	1.44 (0.83 to 2.51)	0.20
	11 to 13 January 2021 (wave 41)	49 (61.3)	31 (38.8)	0.55 (0.31 to 0.99)	0.05	0.93 (0.52 to 1.68)	0.81
	25 to 27 January 2021 (wave 42)	40 (48.2)	43 (51.8)	0.84 (0.48 to 1.44)	0.52	1.80 (1.01 to 3.19)	0.05
Region	East Midlands	40 (52.6)	36 (47.4)	Reference	-	Reference	-
	East of England	52 (58.4)	37 (41.6)	0.81 (0.43 to 1.50)	0.50	0.85 (0.43 to 1.65)	0.62
	London	136 (62.4)	82 (37.6)	0.67 (0.39 to 1.15)	0.15	0.82 (0.46 to 1.48)	0.51
	North (East and West)	103 (57.2)	77 (42.8)	0.86 (0.50 to 1.48)	0.58	1.06 (0.59 to 1.90)	0.85
	Northern Ireland / Scotland / Wales	74 (57.8)	54 (42.2)	0.84 (0.47 to 1.50)	0.56	0.90 (0.48 to 1.68)	0.74
	South East	71 (58.2)	51 (41.8)	0.81 (0.45 to 1.45)	0.48	0.98 (0.52 to 1.87)	0.96
	South West	51 (59.3)	35 (40.7)	0.81 (0.43 to 1.53)	0.51	0.83 (0.42 to 1.63)	0.59
	West Midlands	59 (57.8)	43 (42.2)	0.83 (0.46 to 1.52)	0.55	0.87 (0.45 to 1.66)	0.67
	Yorkshire and the Humber	62 (60.8)	40 (39.2)	0.72 (0.40 to 1.33)	0.30	0.74 (0.39 to 1.43)	0.37

* Wave 31 data were excluded from analyses, therefore these data are not included in the totals in the column headers.

† Adjusting for survey wave, region, sex, age (raw and quadratic term), dependent child in the household, being clinically vulnerable to covid-19, having a household member with a chronic illness, employment status, socioeconomic grade, index of multiple deprivation, highest educational or professional qualification, ethnicity, and living alone.

Table 4. Requesting a test after developing symptoms of covid-19 and associations with survey wave and region. Bolding indicates findings significant at a p<0.001.

Participant characteristics	Level	Did not request a test n=2,433	Requested a test n=525	Odds ratio (95% CI) for requesting a test	p-value	Adjusted odds ratio (95% CI) for requesting a test †	p-value
Survey wave	26 to 27 May 2020 (wave 18)	162 (92.0)	14 (8.0)	Reference	-	Reference	-
	1 to 3 June 2020 (wave 19)	126 (86.9)	19 (13.1)	1.75 (0.85 to 3.60)	0.13	1.72 (0.83 to 3.56)	0.14
	8 to 10 June 2020 (wave 20)	120 (87.6)	17 (12.4)	1.64 (0.78 to 3.43)	0.19	1.36 (0.63 to 2.96)	0.44
	15 to 17 June 2020 (wave 21)	111 (86.7)	17 (13.3)	1.81 (0.86 to 3.77)	0.12	2.06 (0.98 to 4.36)	0.06
	22 to 24 June 2020 (wave 22)	116 (87.2)	17 (12.8)	1.75 (0.85 to 3.64)	0.13	1.78 (0.86 to 3.70)	0.12
	29 June to 1 July 2020 (wave 23)	107 (90.7)	11 (9.3)	1.23 (0.55 to 2.74)	0.62	1.21 (0.54 to 2.73)	0.64
	6 to 8 July 2020 (wave 24)	107 (89.9)	12 (10.1)	1.31 (0.59 to 2.92)	0.51	1.40 (0.62 to 3.17)	0.42
	20 to 22 July 2020 (wave 25)	106 (80.3)	26 (19.7)	2.83 (1.41 to 5.64)	0.003	2.55 (1.26 to 5.17)	0.009
	3 to 5 August 2020 (wave 26)	100 (90.9)	10 (9.1)	1.18 (0.51 to 2.72)	0.71	1.15 (0.48 to 2.74)	0.76
	1 to 2 September 2020 (wave 27)	117 (87.3)	17 (12.7)	1.66 (0.79 to 3.50)	0.18	1.61 (0.75 to 3.46)	0.23
	14 to 16 September 2020 (wave 28)	102 (79.1)	27 (20.9)	3.07 (1.54 to 6.10)	0.001	2.99 (1.50 to 5.98)	0.002
	28 to 30 September 2020 (wave 29)	92 (80.0)	23 (20.0)	2.86 (1.41 to 5.81)	0.004	2.83 (1.38 to 5.81)	0.005
	12 to 14 October 2020 (wave 30)	80 (74.1)	28 (25.9)	**4.11 (2.07 to 8.18)**	**<0.001**	**3.70 (1.83 to 7.50)**	**<0.001**
	26 to 28 October 2020 (wave 31)*	83 (75.5)	27 (24.5)	**3.78 (1.88 to 7.56)**	**<0.001**	-	-
	9 to 11 November 2020 (wave 32)	103 (81.1)	24 (18.9)	2.69 (1.33 to 5.42)	0.006	2.55 (1.26 to 5.19)	0.01
	16 to 18 November 2020 (wave 33)	103 (79.8)	26 (20.2)	2.92 (1.46 to 5.83)	0.002	2.95 (1.45 to 5.98)	0.003
	23 to 25 November 2020 (wave 34)	89 (79.5)	23 (20.5)	2.95 (1.45 to 6.00)	0.003	2.79 (1.35 to 5.79)	0.006
	30 November to 2 December 2020 (wave 35)	97 (82.2)	21 (17.8)	2.49 (1.21 to 5.10)	0.01	2.49 (1.20 to 5.14)	0.01
	7 to 9 December 2020 (wave 36)	88 (78.6)	24 (21.4)	3.17 (1.57 to 6.40)	0.001	3.07 (1.51 to 6.22)	0.002
	14 to 16 December 2020 (wave 37)	80 (72.7)	30 (27.3)	**4.36 (2.20 to 8.64)**	**<0.001**	**4.23 (2.12 to 8.44)**	**<0.001**
	21 to 23 December 2020 (wave 38)	90 (78.3)	25 (21.7)	3.18 (1.58 to 6.41)	0.001	3.06 (1.48 to 6.33)	0.003
	28 to 30 December 2020 (wave 39)	81 (77.9)	23 (22.1)	3.28 (1.61 to 6.70)	0.001	2.58 (1.20 to 5.52)	0.01
	4 to 6 January 2021 (wave 40)	79 (65.8)	41 (34.2)	**6.00 (3.10 to 11.62)**	**<0.001**	**5.71 (2.87 to 11.35)**	**<0.001**
	11 to 13 January 2021 (wave 41)	86 (78.2)	24 (21.8)	3.23 (1.60 to 6.54)	0.001	3.13 (1.54 to 6.39)	0.002
	25 to 27 January 2021 (wave 42)	91 (77.8)	26 (22.2)	3.33 (1.67 to 6.65)	0.001	3.17 (1.55 to 6.47)	0.002
Region	East Midlands	165 (79.7)	42 (20.3)	Reference	-	Reference	-
	East of England	194 (81.2)	45 (18.8)	0.94 (0.59 to 1.52)	0.81	0.92 (0.56 to 1.51)	0.75
	London	512 (83.8)	99 (16.2)	0.76 (0.51 to 1.14)	0.18	0.84 (0.54 to 1.29)	0.42
	North East	104 (80.0)	26 (20.0)	0.97 (0.56 to 1.69)	0.91	0.95 (0.54 to 1.68)	0.86
	North West	265 (79.8)	67 (20.2)	1.00 (0.64 to 1.54)	0.98	1.04 (0.65 to 1.67)	0.86
	Northern Ireland	33 (91.7)	3 (8.3)	0.36 (0.10 to 1.25)	0.11	0.39 (0.11 to 1.35)	0.14
	Scotland	171 (85.1)	30 (14.9)	0.67 (0.40 to 1.13)	0.13	0.71 (0.41 to 1.24)	0.23
	South East	259 (79.4)	67 (20.6)	1.01 (0.66 to 1.57)	0.95	1.07 (0.67 to 1.71)	0.78

	South West	173 (84.0)	33 (16.0)	0.75 (0.45 to 1.25)	0.27	0.80 (0.47 to 1.36)	0.41
	Wales	90 (79.6)	23 (20.4)	1.00 (0.56 to 1.76)	0.99	1.01 (0.55 to 1.84)	0.98
	West Midlands	240 (83.3)	48 (16.7)	0.76 (0.48 to 1.21)	0.25	0.86 (0.53 to 1.41)	0.56
	Yorkshire and the Humber	227 (84.4)	42 (15.6)	0.72 (0.45 to 1.17)	0.18	0.70 (0.42 to 1.16)	0.17

* Wave 31 data were excluded from analyses, therefore these data are not included in the totals in the column headers.

† Adjusting for survey wave, region, sex, age (raw and quadratic term), dependent child in the household, being clinically vulnerable to covid-19, having a household member with a chronic illness, employment status, socioeconomic grade, index of multiple deprivation, highest educational or professional qualification, ethnicity, and living alone.

Table 5. Intention to share details of your close contacts with the NHS contact tracing service and associations with survey wave and region. Bolding indicates findings significant at a $p<0.001$.

Participant characteristics	Level	Probably or definitely would not share details of close contacts or not sure n=9,195	Probably or definitely would share details of close contacts n=34,552	Odds ratio (95% CI) for sharing details of close contacts	p-value	Adjusted odds ratio (95% CI) for sharing details of close contacts †	p-value
Survey wave	1 to 3 June 2020 (wave 19)	453 (24.5)	1,396 (75.5)	Reference	-	Reference	-
	8 to 10 June 2020 (wave 20)	446 (24.0)	1,413 (76.0)	0.94 (0.82 to 1.08)	0.41	1.02 (0.87 to 1.18)	0.84
	15 to 17 June 2020 (wave 21)	471 (25.2)	1,399 (74.8)	0.90 (0.79 to 1.03)	0.12	0.97 (0.84 to 1.12)	0.64
	22 to 24 June 2020 (wave 22)	474 (25.3)	1,396 (74.7)	0.85 (0.74 to 0.97)	0.02	0.90 (0.78 to 1.04)	0.17
	29 June to 1 July 2020 (wave 23)	437 (23.3)	1,442 (76.7)	0.98 (0.86 to 1.13)	0.81	1.04 (0.90 to 1.21)	0.60
	6 to 8 July 2020 (wave 24)	453 (24.0)	1,431 (76.0)	0.91 (0.79 to 1.04)	0.17	0.97 (0.84 to 1.13)	0.70
	20 to 22 July 2020 (wave 25)	452 (24.0)	1,430 (76.0)	0.95 (0.83 to 1.09)	0.46	1.04 (0.90 to 1.20)	0.61
	3 to 5 August 2020 (wave 26)	392 (20.8)	1,497 (79.2)	1.08 (0.93 to 1.24)	0.31	1.18 (1.01 to 1.38)	0.04
	1 to 2 September 2020 (wave 27)	386 (20.5)	1,498 (79.5)	1.13 (0.97 to 1.31)	0.12	1.26 (1.08 to 1.48)	0.004
	14 to 16 September 2020 (wave 28)	363 (19.3)	1,521 (80.7)	1.17 (1.01 to 1.35)	0.04	1.30 (1.11 to 1.52)	0.001
	28 to 30 September 2020 (wave 29)	388 (20.2)	1,533 (79.8)	1.08 (0.94 to 1.25)	0.26	1.17 (1.01 to 1.36)	0.04
	12 to 14 October 2020 (wave 30)	366 (18.6)	1,598 (81.4)	1.23 (1.06 to 1.42)	0.007	1.31 (1.12 to 1.53)	0.001
	26 to 28 October 2020 (wave 31)*	340 (17.6)	1,593 (82.4)	1.25 (1.08 to 1.44)	0.003	-	-
	9 to 11 November 2020 (wave 32)	375 (19.7)	1,527 (80.3)	1.11 (0.97 to 1.29)	0.14	1.18 (1.02 to 1.37)	0.03
	16 to 18 November 2020 (wave 33)	411 (21.3)	1,519 (78.7)	1.05 (0.91 to 1.21)	0.54	1.14 (0.98 to 1.33)	0.08
	23 to 25 November 2020 (wave 34)	337 (17.6)	1,576 (82.4)	1.28 (1.11 to 1.49)	0.001	**1.37 (1.17 to 1.60)**	**<0.001**
	30 November to 2 December 2020 (wave 35)	380 (19.7)	1,552 (80.3)	1.14 (0.99 to 1.32)	0.08	1.28 (1.10 to 1.50)	0.002
	7 to 9 December 2020 (wave 36)	399 (20.0)	1,594 (80.0)	1.11 (0.96 to 1.27)	0.16	1.18 (1.01 to 1.37)	0.03
	14 to 16 December 2020 (wave 37)	405 (21.2)	1,506 (78.8)	1.03 (0.89 to 1.18)	0.70	1.12 (0.96 to 1.30)	0.15
	21 to 23 December 2020 (wave 38)	366 (19.3)	1,534 (80.7)	1.13 (0.98 to 1.30)	0.10	1.23 (1.06 to 1.44)	0.008
	28 to 30 December 2020 (wave 39)	375 (19.5)	1,549 (80.5)	1.13 (0.98 to 1.31)	0.09	1.24 (1.06 to 1.45)	0.007
	4 to 6 January 2021 (wave 40)	357 (18.6)	1,565 (81.4)	1.19 (1.03 to 1.37)	0.02	1.26 (1.08 to 1.47)	0.004
	11 to 13 January 2021 (wave 41)	366 (19.3)	1,529 (80.7)	1.14 (0.98 to 1.32)	0.08	1.23 (1.05 to 1.44)	0.009
	25 to 27 January 2021 (wave 42)	343 (18.1)	1,547 (81.9)	1.22 (1.05 to 1.41)	0.008	1.29 (1.10 to 1.51)	0.002
Region	East Midlands	719 (20.7)	2,760 (79.3)	Reference	-	Reference	-
	East of England	866 (20.3)	3,396 (79.7)	1.10 (0.97 to 1.24)	0.13	0.98 (0.86 to 1.11)	0.75
	London	1,281 (24.9)	3,854 (75.1)	0.86 (0.76 to 0.96)	0.008	0.95 (0.84 to 1.08)	0.42
	North East	382 (19.3)	1,594 (80.7)	1.18 (1.01 to 1.38)	0.03	1.14 (0.97 to 1.34)	0.11
	North West	1,090 (22.1)	3,838 (77.9)	1.00 (0.89 to 1.12)	0.95	0.96 (0.85 to 1.08)	0.50
	Northern Ireland	182 (22.5)	626 (77.5)	1.00 (0.81 to 1.23)	0.97	0.98 (0.79 to 1.21)	0.83
	Scotland	633 (18.4)	2,802 (81.6)	**1.27 (1.11 to 1.44)**	**<0.001**	1.18 (1.03 to 1.35)	0.02
	South East	1,171 (20.0)	4,685 (80.0)	1.13 (1.00 to 1.26)	0.04	0.99 (0.88 to 1.12)	0.90

	South West	728 (19.1)	3,080 (80.9)	1.17 (1.03 to 1.33)	0.01	1.04 (0.91 to 1.19)	0.53
	Wales	391 (18.0)	1,782 (82.0)	1.28 (1.10 to 1.48)	0.001	1.17 (1.00 to 1.37)	0.05
	West Midlands	906 (23.4)	2,969 (76.6)	0.92 (0.81 to 1.04)	0.17	0.92 (0.81 to 1.04)	0.19
	Yorkshire and the Humber	846 (21.1)	3,166 (78.9)	1.06 (0.94 to 1.20)	0.32	1.03 (0.9 to 1.17)	0.70

* Wave 31 data were excluded from analyses, therefore these data are not included in the totals in the column headers.

† Adjusting for survey wave, region, sex, age (raw and quadratic term), dependent child in the household, being clinically vulnerable to covid-19, having a household member with a chronic illness, employment status, socioeconomic grade, index of multiple deprivation, highest educational or professional qualification, ethnicity, and living alone.

Supplementary materials. Associations between personal and clinical characteristics and fully self-isolating after developing symptoms of covid-19. Bolding indicates findings significant at a *p*<0.001.

Participant characteristics	Level	Did not fully self-isolate n=2,769	Fully self-isolated n=688	Odds ratio (95% CI) for fully self-isolating	p-value	Adjusted odds ratio (95% CI) for fully self-isolating †	p-value
Survey wave	Overall	-	-	**χ^2(30)=66.8**	**<0.001**	χ^2(29)=56.7	0.002
Region	Overall	-	-	**χ^2(11)=48.5**	**<0.001**	χ^2(11)=21.4	0.03
Sex	Male	1,618 (83.9)	311 (16.1)	Reference	-	Reference	-
	Female	1,143 (75.5)	371 (24.5)	**1.74 (1.47 to 2.07)**	**<0.001**	**1.92 (1.58 to 2.33)**	**<0.001**
Age (per decade)	Raw age	Mean 34.8 (SD 13.6)	Mean 42.3 (SD 17.0)	**1.38 (1.30 to 1.46)**	**<0.001**	**1.27 (1.18 to 1.36)**	**<0.001**
Age: quadratic (age-mean)^2	-	-	-	-	-	0.9998 (0.9994 to 1.0001)	0.18
Dependent child in household	No	1,034 (72.4)	395 (27.6)	Reference	-	Reference	-
	Yes	1,735 (85.6)	293 (14.4)	**0.46 (0.39 to 0.55)**	**<0.001**	**0.61 (0.49 to 0.75)**	**<0.001**
Clinically vulnerable to covid-19	No	1,927 (80.2)	475 (19.8)	Reference	-	Reference	-
	Yes	785 (79.9)	197 (20.1)	1.01 (0.84 to 1.22)	0.90	0.84 (0.68 to 1.03)	0.10
Household member has chronic illness	No	2126 (79.2)	560 (20.8)	Reference	-	Reference	-
	Yes	586 (84.0)	112 (16.0)	0.69 (0.55 to 0.87)	0.001	0.72 (0.56 to 0.94)	0.01
Employed	No	814 (73.9)	288 (26.1)	Reference	-	Reference	-
	Yes	1,920 (83.3)	384 (16.7)	**0.59 (0.50 to 0.71)**	**<0.001**	0.76 (0.62 to 0.94)	0.01
Socioeconomic grade	ABC1 (high)	1,394 (76.1)	437 (23.9)	Reference	-	Reference	-
	C2DE	1,344 (85.1)	235 (14.9)	**0.57 (0.48 to 0.68)**	**<0.001**	**0.66 (0.54 to 0.81)**	**<0.001**
Index of multiple deprivation fourth	1st (least deprived)	371 (78.6)	101 (21.4)	1.22 (0.93 to 1.59)	0.15	0.95 (0.69 to 1.31)	0.76
	2nd	507 (76.6)	155 (23.4)	1.37 (1.08 to 1.73)	0.009	1.08 (0.83 to 1.42)	0.55
	3rd	802 (81.3)	185 (18.7)	1.06 (0.86 to 1.32)	0.57	0.97 (0.76 to 1.24)	0.83
	4th (most deprived)	1,089 (81.5)	247 (18.5)	Reference	-	Reference	-
	Overall	-	-	χ^2(3)=7.8	0.05	χ^2(3)=0.8	0.85
Highest educational or professional qualification	GCSE, vocational, A-level, or no formal qualifications	1,385 (75.6)	448 (24.4)	Reference	-	Reference	-
	Degree or higher (bachelors, masters, or PhD)	1,384 (85.2)	240 (14.8)	**0.54 (0.45 to 0.65)**	**<0.001**	**0.63 (0.52 to 0.77)**	**<0.001**
Ethnicity	White British	1,841 (78.0)	518 (22.0)	Reference	-	Reference	-
	White other	463 (88.2)	62 (11.8)	**0.45 (0.34 to 0.60)**	**<0.001**	0.64 (0.46 to 0.89)	0.007
	Black and minority ethnicity	455 (81.8)	101 (18.2)	0.78 (0.61 to 0.99)	0.04	1.06 (0.80 to 1.40)	0.68
	Overall	-	-	**χ^2(2)=32.0**	**<0.001**	χ^2(2)=8.1	0.02

Live alone	No	2,336 (80.7)	558 (19.3)	Reference	-	Reference	-
	Yes	433 (76.9)	130 (23.1)	1.22 (0.98 to 1.52)	0.07	0.89 (0.69 to 1.15)	0.38
Work in key sector	No	417 (71.0)	170 (29.0)	Reference	-	Reference	-
	Yes	1,755 (86.7)	270 (13.3)	**0.39 (0.31 to 0.48)**	**<0.001**	**0.50 (0.39 to 0.66)**	**<0.001**
Self-employed‡	No	1,729 (82.6)	363 (17.4)	Reference	-	Reference	-
	Yes	191 (90.1)	21 (9.9)	0.52 (0.33 to 0.82)	0.004	0.67 (0.39 to 1.16)	0.15
Marital status	Single, separated, divorced, or widowed	1,152 (79.2)	302 (20.8)	Reference	-	Reference	-
	Married or partnered	1,500 (80.0)	374 (20.0)	0.98 (0.83 to 1.16)	0.83	0.97 (0.79 to 1.20)	0.80
Ever had covid-19	Think not	1,428 (74.7)	483 (25.3)	Reference	-	Reference	-
	Think so, or confirmed	1,341 (86.7)	205 (13.3)	**0.48 (0.40 to 0.57)**	**<0.001**	**0.58 (0.47 to 0.71)**	**<0.001**
Attribute current symptoms to covid-19§	No	1,402 (80.5)	340 (19.5)	Reference	-	Reference	-
	Yes	429 (79.7)	109 (20.3)	1.10 (0.87 to 1.39)	0.42	1.35 (1.03 to 1.76)	0.03
Hardship‖	Range 3 (least hardship) to 15 (most hardship)	n=2,430; mean 10.4 (SD 2.6)	n=594; mean 9.4 (SD 2.9)	**0.87 (0.84 to 0.90)**	**<0.001**	**0.91 (0.87 to 0.94)**	**<0.001**

Abbreviations: N=number; M=mean; SD=standard deviation; CI=confidence intervals.

For continuous variables, odds ratios represent a one-unit increase in the explanatory variable, apart from for age, where odds ratios represent a 10 year increase in age.

† Adjusting for survey wave, region, sex, age (raw and quadratic term), dependent child in the household, being clinically vulnerable to covid-19, having a household member with a chronic illness, employment status, socioeconomic grade, index of multiple deprivation, highest educational or professional qualification, ethnicity, and living alone.

‡ Not adjusting for employment status as by definition all people asked whether they were self-employed were working. Due to a cell count of zero for self-employment by region (Northern Ireland), in the multivariable analysis we grouped together participants in Scotland, Wales and Northern Ireland and the North East and North West into single groups.

§ Item added to survey in wave 21 (15 June 2020)

‖ Items added to survey in wave 14 (27 April 2020)

Supplementary materials. Self-reported reasons for not adhering to test, trace and isolate behaviours.

Self-isolation in those not adherent to duration adjusted self-isolation, excluding those who tested negative (n=698)

Self-reported reasons for having left home since having developed symptoms (participants could select multiple response options)	n	%
To go to the shops, for groceries/pharmacy	150	21.5
To go out to work	110	15.8
To go to the shops, for things other than groceries/pharmacy	109	15.6
My symptoms did not persist / were temporary	106	15.2
For a medical need (other than coronavirus)	105	15.0
To go for a walk or some other exercise	103	14.8
My symptoms were only mild	101	14.5
My symptoms got better	97	13.9
I don't think it is necessary for me to stay at home	92	13.2
I was too bored	85	12.2
To help or provide care for a vulnerable person	83	11.9
To meet up with friends and/or family	79	11.3
I was too depressed or anxious	78	11.2
I didn’t think it was that risky*	49	9.8
I was too lonely	68	9.7
My symptoms got worse	59	8.5
To get or return a test for coronavirus†	32	7.6

* Item added on 23 November 2020 (wave 34); baseline n=498.
† Item added on 23 November 2020 (wave 34); baseline n=421. Different baseline n due to exclusion of those who tested negative.

Requesting a test (n=2,230).

Self-reported reasons for not requesting a test to confirm whether you had coronavirus (participants could select multiple response options)	n	%
I didn't think my symptoms were due to coronavirus	467	20.9
My symptoms improved*	196	16.9
My symptoms were only mild*	189	16.3
My symptoms were only mild or improved §	144	13.5
I hadn't been in contact with anyone who had coronavirus recently	291	13.0
I thought I only needed to self-isolate (not leaving the home at all)	256	11.5
I didn't want to use a test that could have gone to someone else who needed it more	247	11.1
I didn't think I was eligible to get a test	245	11.0
I was worried about how colleagues/my employer would react if I tested positive †	188	10.0
I didn't know how to request a test	208	9.3
I was sure I had coronavirus, so I didn't see a reason to take a test	206	9.2
I have already had coronavirus, so didn't see a reason to take a test	198	8.9
I was worried what friends or family would think about me if I tested positive†	158	8.4
I thought it would take too long to get a test result §	86	8.0
I thought the test would be uncomfortable or painful	171	7.7
I didn’t think tests were available near me ‡	96	7.7
I didn't think the test would be accurate	169	7.6
I didn't want to know the results of a test	168	7.5
I didn’t want to have a coronavirus test result on my medical record §	77	7.2
I didn’t want to have to ask the people who I live or who I have met with to self-isolate §	74	6.9
I didn't know what the test involved	151	6.8
I didn’t want to have to self-isolate §	70	6.5
I didn’t want to increase the case numbers in my local area because this leads to tighter restrictions §	70	6.5
I didn’t want the police or other public bodies to be notified §	64	6.0
I was worried that I might get fined §	59	5.5

* Items last included on 14 October 2020 (wave 30); baseline n=1,160.
† Items added on 29 June 2020 (wave 23); baseline n=1,882.
‡ Item added on 28 September (wave 29); baseline n=1,242.
§ Items added on 26 October 2020 (wave 31); baseline n=1,070.

Intention to share details of close contacts (n=45,720)

Self-reported reasons that would stop you passing details of your recent close contacts to the NHS (participants could select multiple response options)	n	%
I don't know if the data will be secure and confidential	6,674	14.6
I don't think the contact tracing system is accurate and reliable	6,355	13.9
I don't know what will happen to the data	5,929	13.0
It would cause stress to my contacts	3,409	7.5
It would cause inconvenience to my contacts	2,766	6.0
It would cause loss of income to my contacts	2,431	5.3
I don't want to be responsible of other people being told to self-isolate	2,163	4.7
My contacts would be angry	2,083	4.6
I am worried about being found out by my contacts	1,571	3.4
None of the above, I would share details of recent close contacts with the NHS if asked to do so (single code)	29,102	63.6

Supplementary materials. Associations between personal and clinical characteristics and requesting a test after developing symptoms of covid-19. Bolding indicates findings significant at a *p*<0.001.

Participant characteristics	Level	Did not request a test n=2,433	Requested a test n=525	Odds ratio (95% CI) for requesting a test	p-value	Adjusted odds ratio (95% CI) for requesting a test †	p-value
Survey wave	Overall	-	-	**$\chi^2(24)=79.9$**	**<0.001**	**$\chi^2(23)=66.6$**	**<0.001**
Region	Overall	-	-	$\chi^2(11)=11.3$	0.42	$\chi^2(11)=8.7$	0.65
Sex	Male	1,380 (85.0)	243 (15.0)	Reference	-	Reference	-
	Female	1,041 (78.8)	280 (21.2)	**1.54 (1.27 to 1.87)**	**<0.001**	**1.47 (1.20 to 1.81)**	**<0.001**
Age (per decade)	Raw age	Mean 36.8 (SD 14.9)	Mean 36.4 (SD 13.6)	0.99 (0.93 to 1.05)	0.67	0.92 (0.83 to 1.02)	0.11
Age: quadratic $(\text{age-mean})^2$	-	-	-	-	-	0.9995 (0.999 to 1.000)	0.05
Dependent child in household	No	1,023 (82.6)	216 (17.4)	Reference	-	Reference	-
	Yes	1,410 (82.0)	309 (18.0)	1.02 (0.84 to 1.23)	0.86	0.98 (0.78 to 1.22)	0.84
Clinically vulnerable to covid-19	No	1,706 (82.3)	367 (17.7)	Reference	-	Reference	-
	Yes	678 (82.1)	148 (17.9)	1.02 (0.83 to 1.26)	0.86	1.07 (0.85 to 1.34)	0.58
Household member has chronic illness	No	1,888 (82.2)	409 (17.8)	Reference	-	Reference	-
	Yes	496 (82.4)	106 (17.6)	0.99 (0.78 to 1.25)	0.93	1.14 (0.88 to 1.46)	0.32
Employed	No	832 (84.2)	156 (15.8)	Reference	-	Reference	-
	Yes	1,563 (81.3)	360 (18.7)	1.22 (0.99 to 1.50)	0.06	1.14 (0.89 to 1.46)	0.30
Socioeconomic grade	ABC1 (high)	1,298 (81.7)	291 (18.3)	Reference	-	Reference	-
	C2DE	1,098 (83.1)	223 (16.9)	0.90 (0.74 to 1.09)	0.28	0.90 (0.73 to 1.11)	0.32
Index of multiple deprivation fourth	1st (least deprived)	336 (81.8)	75 (18.2)	1.13 (0.84 to 1.52)	0.43	1.14 (0.82 to 1.59)	0.44
	2nd	443 (80.1)	110 (19.9)	1.25 (0.96 to 1.63)	0.10	1.21 (0.90 to 1.61)	0.21
	3rd	712 (82.5)	151 (17.5)	1.07 (0.84 to 1.35)	0.58	1.08 (0.84 to 1.40)	0.55
	4th (most deprived)	942 (83.3)	189 (16.7)	Reference	-	Reference	-
	Overall	-	-	$\chi^2(3)=2.8$	0.42	$\chi^2(3)=1.7$	0.64
Highest educational or professional qualification	GCSE, vocational, A-level, or no formal qualifications	1,318 (81.8)	294 (18.2)	Reference	-	Reference	-
	Degree or higher (bachelors, masters, or PhD)	1,115 (82.8)	231 (17.2)	0.93 (0.77 to 1.12)	0.43	1.02 (0.83 to 1.25)	0.88
Ethnicity	White British	1,663 (81.7)	373 (18.3)	Reference	-	Reference	-
	White other	370 (86.9)	56 (13.1)	0.67 (0.49 to 0.91)	0.01	0.72 (0.52 to 1.00)	0.05
	Black and minority ethnicity	385 (80.2)	95 (19.8)	1.09 (0.85 to 1.4)	0.51	1.04 (0.79 to 1.37)	0.78
	Overall	-	-	$\chi^2(2)=7.9$	0.02	$\chi^2(2)=4.2$	0.12
Live alone	No	2043 (82.1)	445 (17.9)	Reference	-	Reference	-

	Yes	390 (83.0)	80 (17.0)	0.95 (0.73 to 1.23)	0.69	1.02 (0.75 to 1.38)	0.90
Work in key sector	No	427 (82.8)	89 (17.2)	Reference	-	Reference	-
	Yes	1,365 (81.3)	315 (18.8)	1.12 (0.86 to 1.45)	0.40	1.37 (1.03 to 1.82)	0.03
Self-employed	No	1,425 (81.4)	325 (18.6)	Reference	-	Reference	-
	Yes	138 (79.8)	35 (20.2)	1.10 (0.74 to 1.63)	0.65	1.18 (0.76 to 1.84)	0.46
Marital status	Single, separated, divorced, or widowed	1,031 (84.0)	196 (16.0)	Reference	-	Reference	-
	Married or partnered	1,306 (80.8)	311 (19.2)	1.25 (1.03 to 1.53)	0.02	1.19 (0.93 to 1.51)	0.16
Ever had covid-19	Think not	1,410 (82.9)	291 (17.1)	Reference	-	Reference	-
	Think so, or confirmed	1,023 (81.4)	234 (18.6)	1.10 (0.91 to 1.33)	0.33	1.11 (0.90 to 1.37)	0.31
Attribute current symptoms to covid-19§	No	1,596 (83.3)	320 (16.7)	Reference	-	Reference	-
	Yes	429 (73.5)	155 (26.5)	**1.78 (1.43 to 2.22)**	**<0.001**	**1.73 (1.37 to 2.19)**	**<0.001**
Hardship	Range 3 (least hardship) to 15 (most hardship)	n=2,310; mean 10.3 (SD 2.7)	n=507; mean 9.9 (SD 2.8)	0.95 (0.91 to 0.98)	0.003	0.93 (0.90 to 0.97)	0.001

Abbreviations: N=number; M=mean; SD=standard deviation; CI=confidence intervals.

For continuous variables, odds ratios represent a one-unit increase in the explanatory variable, apart from for age, where odds ratios represent a 10 year increase in age.

† Adjusting for survey wave, region, sex, age (raw and quadratic term), dependent child in the household, being clinically vulnerable to covid-19, having a household member with a chronic illness, employment status, socioeconomic grade, index of multiple deprivation, highest educational or professional qualification, ethnicity, and living alone.

§This item was introduced to the questionnaire in wave 21 (15 June 2020).
